# Recyclable IPN Photocatalysts Supported by Polymer Matrices: From Soluble Copolymers to Core-Polymer Brush Shell Nanostructures

**DOI:** 10.1021/jacs.5c22227

**Published:** 2026-02-11

**Authors:** Elena Avanzini, Alessio Lo Bocchiaro, Agata Checcozzo, Luis Izquierdo-Aranda, Eric Ruzicka, Jorge Humbrías-Martín, Gianluca Gazzola, Francesca Lorandi, Luca Dell’Amico, Edmondo M. Benetti

**Affiliations:** † Laboratory for Macromolecular and Organic Chemistry, Department of Chemical Sciences, University of Padova, Via Marzolo 1, 35131 Padova, Italy; ‡ Sustainable Synthesis and Catalysis, Department of Chemical Sciences, University of Padova, Via Marzolo 1, 35131 Padova, Italy

## Abstract

Functional polymeric materials have recently emerged as promising supports for organic photocatalysts (PCs), yet the effects of PC design and polymer architecture on catalytic performance remain underexplored. In this study, we present a versatile strategy for small-molecule activation using carbazolyl-dicyanobenzene-based PCs featuring an isophthalonitrile (IPN) core. These PCs feature thermally activated delayed fluorescence (TADF) properties and long-lived excited states, although they also suffer from an intrinsic chemical fragility that hampers their recyclability. A library of IPN derivatives was synthesized, characterized, and integrated into either soluble copolymers (by exploiting controlled radical polymerization) or grafted from inert silica nanoparticles (NPs) to yield PC-loaded spherical polymer brushes. Although soluble copolymers showed catalytic activity comparable to that of free PCs while testing model Povarov-type cycloadditions, PC recovery was only modest and relied on precipitation steps. In contrast, PC-loaded brushes on NPs achieved high yields and enabled efficient catalyst recovery and reuse (≥90%) over multiple cycles. This comparative approach highlights how the nature of the polymeric support, soluble *vs.* nanostructured, critically influences both catalytic performance and recyclability. More generally, the incorporation of IPN PCs into advanced polymer scaffolds is demonstrated to enhance their applicability, cost-efficiency, and sustainability.

## Graphical abstract

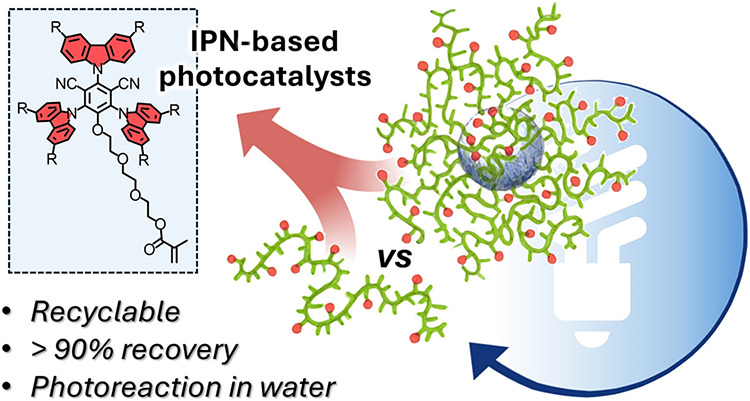

## Introduction

The immobilization of organic and organometallic catalysts on supporting materials has progressively emerged as an advantageous strategy to integrate catalytic activity with recyclability and customizable material properties.^1,2^ The characteristics of engineered supports can be tailored to meet specific requirements, such as improving thermal and chemical stability,^3^ addressing solubility issues,^4^ or promoting selective interactions.^5,6^ In this way, a more sustainable design for catalytic materials can be accomplished while simultaneously broadening the tuning capability for their functional properties, stability, and activity. This approach can be further strengthened by developing “greener” chemistries that exploit more efficient catalysts and/or rely on visible light under mild conditions.^7–9^ In particular, a critical challenge in achieving greater sustainability is ensuring catalyst reuse and recycling—features that are often limited by the reduced stability of the active molecules and the complex processes required for their purification.

To this aim, polymers represent advantageous and extremely versatile supports^10^ owing to their low cost, ease of synthesis, and tailorable properties.^11,12^ Especially in the case of organic photocatalysts (PCs), the application of functional polymeric supports has recently bloomed, involving different applications.^13,14^

Polymeric supports can be engineered to yield different architectures such as networks, brushes, gels, or nanoparticles, enabling control over catalyst distribution, accessibility, and photostability.^15–18^

For instance, the team of Pester developed fluorescein-functionalized polymer brushes grafted on glass beads, demonstrating efficient photocatalytic activity in both condensation and radical reactions, which was coupled with the possibility of recycling the supported PCs.^19^ Similarly, SiO_*x*_ beads were decorated with cross-linked polymer brushes including porphyrin functionalities, providing a polymer brush-supported photocatalytic system capable of disrupting bacterial membranes during water treatment through light-mediated singlet oxygen sensitization.^20^

Following an alternative approach, Sumerlin and co-workers synthesized a polymer containing eosin Y moieties, which showed effective catalytic performance in oxidative transformations and photoredox polymerizations, with the added benefit of being easily recoverable and reusable.^21^ The same PC was exploited by Cai and co-workers, who designed a heterogeneous system based on interpenetrating polymer networks incorporating eosin Y and tertiary amines. These PC-loaded materials enabled to achieve photoinduced electron transfer-reversible addition–fragmentation chain transfer (PET-RAFT) polymerization and showed excellent oxygen tolerance in both aqueous and nonaqueous environments.^22^

Although the above-mentioned examples demonstrated the potential of functional polymeric materials as versatile supports for organic PCs, little has been explored regarding the effects of PC design and how polymer characteristics can be exploited to broaden the applicability of specific catalysts. For instance, copolymerization of PC-bearing monomers with hydrophilic comonomers could allow one to perform diverse types of organic reactions within aqueous environments while using PCs insoluble in water.^19,20,23^

More generally, little attention has been paid to investigating the influence of the type of polymeric support employed for catalyzing organic transformations by comparing PC-containing copolymers dissolved in the reaction medium with more complex architectures such as polymeric core–shell nano/micro-objects.

Inspired by these intriguing challenges, we introduce here an efficient strategy for photoredox small-molecule transformations that exploits PC-functionalized polymers with a controlled architecture alternatively deployed in solution or in the form of spherical brushes grafted on inert silica nanoparticles (NPs).

We specifically focused on carbazolyl dicyanobenzenes presenting isophthalonitrile (IPN) cores as PCs.^24^ Since their introduction by Adachi and co-workers, these scaffolds rapidly became the most applied PCs in synthetic photocatalysis, thanks to (i) a well-balanced redox window, (ii) a long excited-state lifetime, and (iii) a thermally activated delayed fluorescence (TADF) behavior.^25^ TADF occurs when a molecule’s excited state undergoes reverse intersystem crossing (RISC) from a triplet state (T_1_) back to a singlet state (S_1_), facilitated by a small energy gap (Δ*E*_ST_ < 0.3 eV) between T_1_ and S_1_, and allowing thermal energy to activate the transition. Especially for PCs, a long S_1_ lifetime (≥10 ns) is crucial for achieving high efficiency, as it permits a sufficient time for diffusion to the substrate. TADF PCs exhibit exceptionally long excited-state lifetimes that can reach microseconds. Another significant advantage of IPN-based PCs is their structural flexibility, which enables them to span a wide range of redox potentials. Numerous PCs that cover a broad range of potentials already exist. These include acridinium salts,^26^ flavins,^27^ phenazines,^28^ phenothiazines,^29^ quinones^30^ and naphthocromenones^31^. In this context, IPN-based PCs stand out for their exceptional versatility,^32–41^ which is enabled by their accessible functionalization with various substituents, thus substantially enlarging the toolbox of available IPNs.^9,42^

The comprehensive physicochemical characterization of a diverse set of molecularly designed IPN-based platforms, and their subsequent application in model organic transformations allowed us to identify the best-performing PCs, which were later incorporated within different copolymer formulations.

When employed to catalyze organic reactions, copolymers applied in solution and including PCs as comonomers provided yields comparable to those obtained by employing “free” PCs. However, PC recovery following consecutive reaction cycles required copolymer precipitation in selective solvents, which limited the recovery efficiency. In contrast, NPs featuring PC-loaded copolymer brushes as shells guaranteed high reaction yields (up to 60%) while ensuring very efficient recovery (≥90%) and subsequent recycling. In addition, we show that, when incorporated within copolymers, IPN-based PCs can be efficiently used in aqueous media without the need for any additional surfactants, opening the way to their general use in solvents that are generally precluded from IPN photocatalysis.

Despite the clear advantages of IPN-based PCs, their preparation may require significant synthetic efforts, making them less readily accessible and potentially more costly than off-the-shelf organic PCs. Hence, this study highlights how integrating IPN-based PCs within polymeric supports not only broadens their applicability but also significantly facilitates their recycling and enables their use in aqueous media.

## Results and Discussion

The molecular design of PCs relied on an IPN core presenting three differently substituted carbazole (Cz) or diphenylamine (DPA) units and a methacrylate function that extends from the core through a triethylene glycol “spacer”. This molecular architecture enabled the generation of a series of PC-methacrylates (PCMAs) that can be copolymerized with other functional monomers by exploiting controlled radical polymerization processes (Figure 1a).

**Figure 1:**
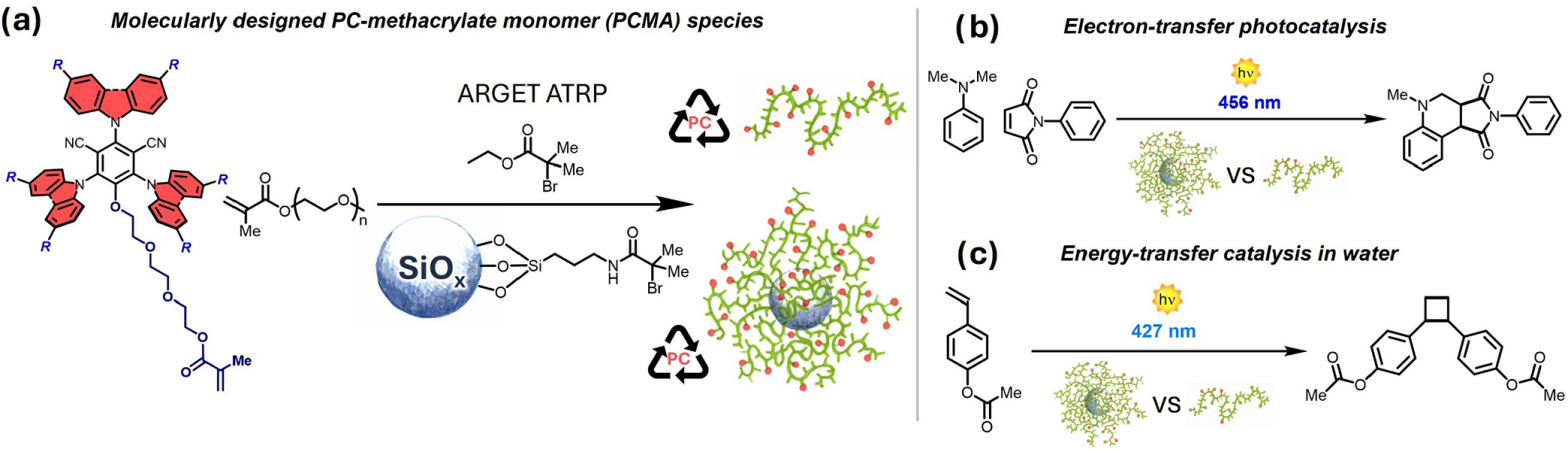
(a) Activator regenerated by electron transfer atom transfer radical polymerization (ARGET ATRP) of molecularly designed PC-methacrylate (PCMA) species and oligo­(ethylene glycol) methyl ether methacrylate (OEGMA, with *M_n_* ∼ 500 g mol^–1^) (OEGMA) provided P­(PCMA-*co*-OEGMA) statistical copolymers. Surface-initiated ARGET ATRP (SI-ARGET ATRP) from initiator-bearing SiO_*x*_ NPs generated PC-loaded core-polymer brush shell NPs. (b, c) Benchmark reactions performed for assessing photocatalytic activity, recyclability, and activity in water of PCs supported by different polymer matrices.

Our first objective was to identify the best-performing PC through photophysical and electrochemical characterizations. To this end, we initially synthesized a library of model PCs (Figure 2 and Scheme S1), whereby the unsaturated function of the methacrylate moiety was replaced with an alkyl analogue to rule out possible interferences of the reactive double bond during physicochemical characterizations.

**Figure 2:**
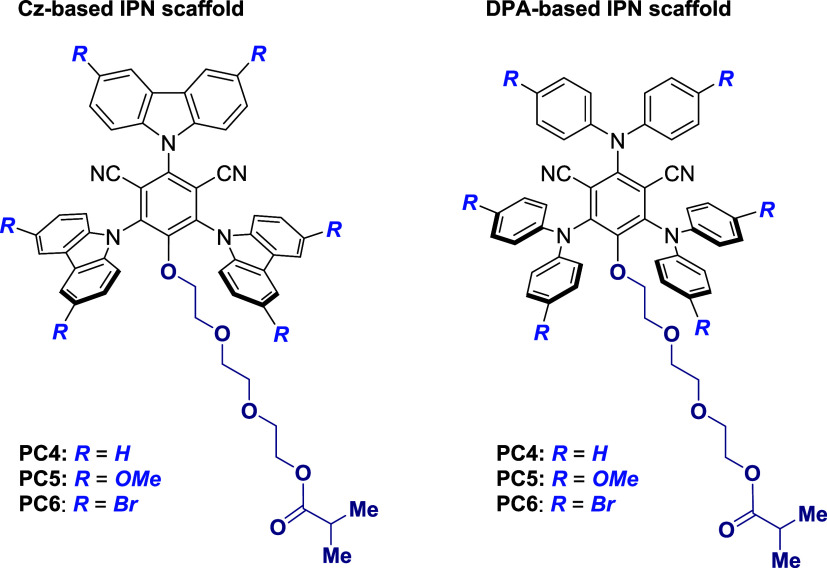
Structures of PCs featuring an IPN core, carbazole/diphenylamine substituents, a triethylene glycol spacer, and an ester moiety as the saturated analogue of a methacrylate function.

The introduction of different substituents on the IPN core influenced the photophysical properties of the PCs. In particular, strategic variation of the functional groups on the Cz and DPA moieties allowed us to finely tune the excited-state lifetimes (τ) and redox potentials of the PCs, thus optimizing their photoredox behavior. Absorption and emission spectra were recorded and analyzed for all of the model PCs (Table 1).^43^

**Table 1:**
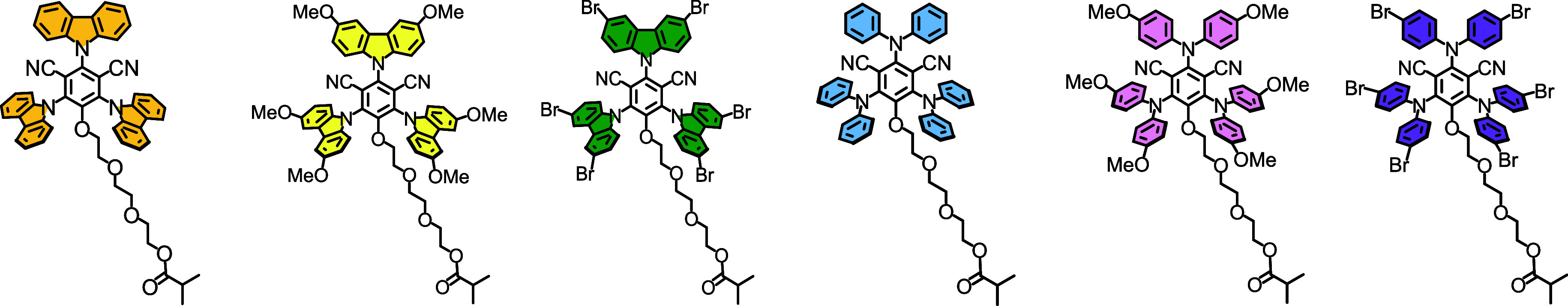
Photophysical and Electrochemical Characterizations of Molecularly Designed PCs^e^

	PC1	PC2	PC3	PC4	PC5	PC6	no PC
λ_int_ (nm)	458	502	450	455	505	464	
*E*_0,0_ (eV)°	2.70	2.47	2.75	2.72	2.45	2.67	
*E*_1/2_(PC/PC^•–^)^a^ (V)	–1.30	–1.36	–1.17	–1.63	–1.75	–1.48	
*E*_p,a_(PC^•+^/PC)^b^ (V)	+1.21	+1.22	+1.75	+1.26	+0.87	+1.39	
*E*_1/2_(PC*/PC^•‑^)^a^ (V)	+1.40	+1.11	+1.58	+1.09	+0.70	+1.19	
*E*_p,a_(PC^•+^/PC*)^b^ (V)	–1.49	–1.25	–1.00	–1.46	–1.58	–1.28	
τ_p_ (ns)^d^	14.4	3.2	2.3	7.2	1.5	2.3	
τ_d_ (ns)^d^	1.465	Nd^c^	817	86 × 10^3^	Nd^c^	66 × 10^3^	
^1^H NMR yield (%)^d^	70	40	52	84	45	74	15

a Half-wave potential.

b Anodic peak potential.

c Delayed fluorescence was not detected. ^d 1^H NMR yields of Povarov reaction (Figure 1b).^44^ Reaction conditions: 3 mol % PC was used for each reaction, 0.1 mmol of *N*-phenylmaleimide, 0.2 mmol of *N*,*N*-dimethylaniline in ACN at room temperature, irradiating for 4 h under blue light (λ_max_ = 456 nm, intensity 150 mW cm^–2^) and yields were calculated using 1,3,5-trimethoxybenzene as an internal standard.

d Prompt and delayed fluorescence lifetimes were measured in a 10^–3^ mM solution in ACN for each photocatalyst. Direct fluorescence (τ_p_) was measured using time-correlated single-photon counting (TCSPC) while long delayed fluorescence was recorded with multichannel scaling (MCS). All fluorescence decays were analyzed with the Fluoracle Software using the IRF convolution fitting procedure.

e Potentials are reported in V vs. saturated calomel electrode (SCE).

Additionally, fluorescence measurements provided the excited-state lifetimes (Tables 1 and S3) for each PC. These measurements were crucial to confirm that the synthesized PCs exhibit a sufficiently long excited-state lifetime to be active in photocatalysis (*i.e.*, > 1 ns). Particular attention was given not only the presence of prompt fluorescence (τ_p_) but also delayed fluorescence (τ_d_), the latter being indicative of the preservation of reverse intersystem crossing, and thus of TADF behavior.^45,46^

The fluorescence measurements revealed that OMe-bearing PCs did not exhibit TADF behavior, regardless of presenting Cz or DPA units. At the same time, the other PCs possessing DPA moieties showed a delayed fluorescence >60 μs (Table 1).

Finally, on those PCs presenting TADF, lifetime measurements were performed at 77 K to confirm that the second lifetime component corresponded to delayed fluorescence rather than phosphorescence (Table S4).^47^ In fact, by repeating the measurement at very low temperature, the thermal energy supplied to the system is no longer sufficient to enable reverse intersystem crossing, and as a result, the delayed fluorescence component (τ_d_) can no longer be detected.^48^ Overall, the emission analysis proved that four of the synthesized compounds exhibited TADF behavior, *i.e.*, their fluorescence profiles displayed a double-exponential decay pattern.

### Structure–Property Relationships for Model PCs

Aiming at determining how the structural changes in the chromophore unit determined the physicochemical properties of the model PCs, we performed a series of spectroscopic and optical characterizations. The absorption and emission spectra of both carbazole (Cz) and diphenylamino (DPA)-based IPN cores revealed a clear influence of the structural modifications on the photophysical behavior of the PCs. In both sets of measurements (Figure 3), the introduction of electro-donating groups (EDGs) enhanced the donor–acceptor characteristics, leading to a red-shifted absorption maxima and a broadening of the emission bands, which suggests a boosted intramolecular charge-transfer (CT) character. Specifically, the absorption profiles showed that an increase in conjugation or the replacement of heteroatoms with EDGs promoted bathochromic shifts—as can be observed for PC2 and PC5—which are consistent with the trend reported for phenoxazine/dihydroacridine and acridinium scaffolds.^42^ Correspondingly, the normalized emission spectra (Figure 3) exhibited larger Stokes shifts, reflecting stronger CT excited states and partial stabilization of the S_1_–CT or T_1_–CT states. This effect generally correlates with longer excited-state lifetimes, as it was observed for highly conjugated dihydrophenazine and acridinium derivatives.^42^ Overall, these data support a direct relationship in which enhanced conjugation and well-defined HOMO–LUMO (donor–acceptor) separation modulate the optical windows (λ_abs_/λ_em_).

**Figure 3:**
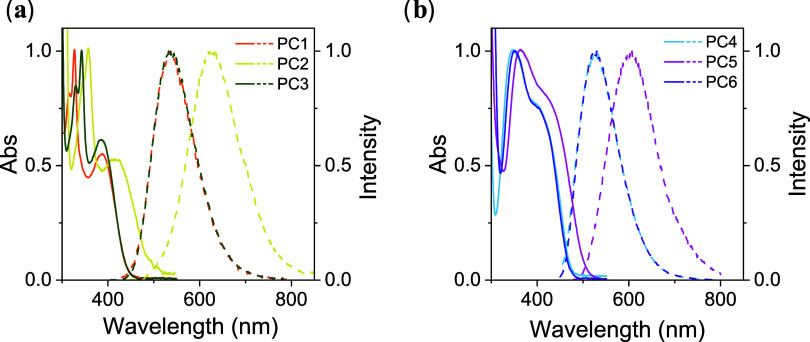
Absorption (solid lines) and emission (dashed lines) profiles recorded for (a) PC1–3 and (b) PC4–6. Experimental conditions for the measurements are provided in the Supporting Information.

Relevantly, the redox potential is finely modulated by the diverse substitution patterns with EDGs, such as -OMe for PC2 and PC5 (Figure 3), and EWGs, such as Br for PC3 and PC6 (Figure 3). In this context, it is important to emphasize that the presence of the triethylene glycol substituent does not significantly alter the properties of the model PCs, which are closely correlated to the characteristics of the original, fully substituted Cz or DPA counterparts.^49,50^

Additionally, the electrochemical data of the IPN-based photocatalysts PC1–PC6 (Table 1 and Figure 4) reveal a finely tunable redox window resulting from structural variations at the Cz or DPA scaffolds, in line with the structure–property principles.^42^ The excited state reduction potentials *E*_1/2_(PC*/PC^•–^) span from +1.58 V to +0.70 V vs SCE, while the excited state oxidation potentials *E*_p,a_(PC^•+^/PC*) range from −1.58 V to −1.00 V, defining broad oxidative and reductive capabilities.

**Figure 4:**
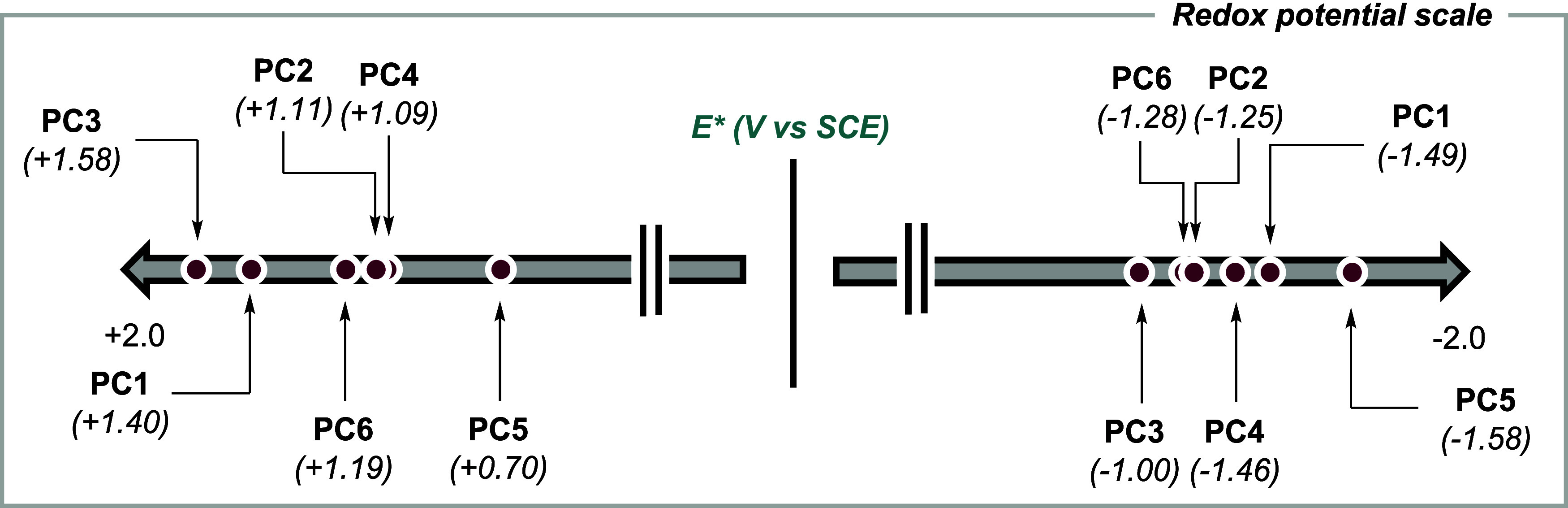
Excited-state electrochemical series of model photocatalysts PC1–6.

### Synthetic Applications

All model PCs were subsequently tested under the same conditions in a benchmark Povarov-type reaction (Figure 1b and Scheme S4)—a formal [4 + 2] cycloaddition that generates valuable heterocyclic structures starting from the formation of N-centered radical cations.^51^ *N,N*-Dimethylaniline (DMA) and *N*-phenylmaleimide were selected as model substrates for the reaction.^42^

Relevantly, the redox potential of *N,N*-dimethylaniline is *E* ^0^(DMA^•+^/DMA) = 0.80 V *vs.* SCE, whereas the reduction of O_2_ to superoxide anion, which serves to close the catalytic cycle in the Povarov-type reaction (Scheme S5), is *E* ^0^(O_2_/ O_2_^•–^) = −0.64 V *vs.* SCE. According to the redox potentials reported in Table 1 and Figure 4, the excited-state PCs have suitable potentials for oxidizing DMA, forming the PC radical anion, PC^•–^, which can then react with O_2_ to regenerate the ground-state PC. Thus, the photoreaction proceeds through a reductive quenching pathway (Scheme S5).^44^

We next investigated the potential of the diverse substitution patterns for different reactions. All of the PCs were tested under the same conditions, at a concentration of 3 mol% and by irradiating the reaction mixture with a Kessil lamp (λ_max_ = 456 nm, intensity 150 mW cm^–2^). As reported in Table 1, all PCs showed catalytic activity, as the control reaction performed without PC provided only a 15% yield (measured by ^1^H NMR) after 4 h of irradiation. In particular, PC1, PC4, and PC6 showed yields ≥70% after a comparable irradiation time (Table 1), reaching and in some cases surpassing the performance of other PCs applied on the same benchmark reaction.^31,44,52^ In fact, among the library of PCs reported in this work, PC1, PC4, and PC6 displayed the most balanced redox profiles, combining excited-state oxidative potentials above +1.1 V and reductive potentials below −1.2 V. These features, as well as their persistence of TADF-related delayed fluorescence lifetimes, support their superior performance in the benchmark reaction. On the other hand, we speculate that the lower effectiveness observed for PC2 and PC5 was due to a fast back electron transfer reaction,^49^ while the lower reducing ability of PC3 (Table 1) may be responsible for its more modest activity.^50^

Having established the best-performing PCs within a benchmark reaction, we synthesized their methacrylate-bearing derivatives PC1MA, PC4MA, and PC6MA, respectively. These were copolymerized with oligo­(ethylene glycol) methyl ether methacrylate (OEGMA, with *M_n_* ∼ 500 g mol^–1^) through activator regenerated by electron transfer atom transfer radical polymerization (ARGET ATRP)^53,54^ using a comonomer feed containing 5 mol % of PCMA, to afford P­(PCMA-*co*-OEGMA) statistical copolymers (Figure 1a and Scheme S2).

OEGMA was selected as a comonomer due to its amphiphilic nature, enabling the synthesis of copolymers that are soluble both in water and in organic solvents. This is especially relevant, since POEGMA-based polymeric supports can not only facilitate the recycling of PCs but also allow one to perform photocatalytic reactions in benign aqueous environments, thus further broadening the application of supported PCs toward more sustainable chemical processes.

ARGET ATRP of the different PCMAs and OEGMA was performed using ethyl α-bromoisobutyrate (EBiB) as initiator, Cu^II^Br_2_/L as catalyst, where the ligand L was tris­(2-pyridylmethyl)­amine (TPMA), and sodium ascorbate (NaAsc) as reducing agent for the (re)­generation of Cu^I^-based ATRP activators.

NMR and UV–vis characterization of the obtained copolymers confirmed the successful incorporation of PC1MA, PC4MA, and PC6MA, with relative concentrations ranging from 2 to 4.5 mol % (Table S1).

The photophysical properties of the different copolymers did not show significant differences with respect to the properties of the PCs prior to copolymerization. Absorbance, fluorescence, redox potentials, and lifetimes of free PCs were all comparable to those of the corresponding P­(PCMA-*c* *o*-OEGMA)­s (Tables S3 and S4). Relevantly, these results highlighted that the active cores of the different PCs are not interacting with the copolymer backbones and their properties are not affected by the incorporation within copolymer chains.

### Recyclability Tests

Statistical copolymers P­(PC1MA-*c* *o*-OEGMA), P­(PC4MA-*c* *o*-OEGMA), and P­(PC6MA-*c* *o*-OEGMA) were subsequently employed as supported PCs in the Povarov-type benchmark reaction (Figure 1b). The copolymer concentration was initially set to reach an overall PC content that was previously proven as the best performing within test reactions with “free” PC in solution.^31^

After 4 h of blue-light irradiation (Kessil lamp, λ_max_ = 456 nm, intensity 150 mW cm^–2^), a catalyst loading of 3.0 mol% resulted in nearly identical NMR yields compared to those obtained with the free PCs (Figure 3b and Table 2). When the PC concentration decreased to 1 mol%, the yield remained nearly constant (70–89%). In contrast, by using 0.5 mol% PC loading, the yield dropped to 40% after the same reaction time. Prolonging the irradiation to 14 h enabled an increase in the conversion to >70%. A control experiment conducted under identical conditions in the absence of PC gave only 30% yield after 14 h (entry 12 in Table 2).

**Table 2:**
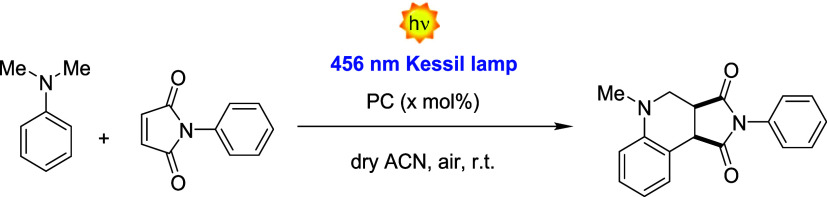
^1^H-NMR Yields in Povarov Cycloaddition between *N*,*N*-dimethylaniline (DMA) and *N*-Phenylmaleimide (Figure 1b) Using Different Supported PCs

entry^a^	PC	PC loading (mol %)^b^	reaction time (h)	^1^H NMR yield (%)^c^	weight recovery (%)^d^
1	P(PC1MA-*co*-OEGMA)	3	4	71	60
2	P(PC1MA-*co*-OEGMA)	1	4	72	
3	P(PC1MA-*co*-OEGMA)	0.5	14	70	
4	P(PC4MA-*co*-OEGMA)	3	4	84	63
5	P(PC4MA-*co*-OEGMA)	1	4	89	
6	P(PC4MA-*co*-OEGMA)	0.5	14	86	
7	P(PC6MA-*co*-OEGMA)	3	4	74	58
8	P(PC6MA-*co*-OEGMA)	1	4	75	
9	P(PC6MA-*co*-OEGMA)	0.5	14	74	
11	SiO_2_–P(PC1MA-*co*-OEGMA)	0.1	14	62	91
12			14	30	

a Reaction conditions: 0.1 mmol of *N*-phenylmaleimide, 0.2 mmol of DMA in ACN at room temperature, irradiated with a Kessil lamp (λ_max_ = 456 nm) at 50% intensity.

b Based on the estimated incorporation of the photocatalyst units within the copolymer determined by UV–vis spectroscopy.

c 1,3,5-Trimethoxybenzene was used as an internal standard.

d Recovered amount of supported PC after one reaction cycle.

Recovery of PCs-bearing copolymers was accomplished through the addition of an excess of EtO_2_ to the crude mixture, followed by copolymer precipitation and subsequent centrifugation. However, it is important to emphasize that the recovery was only modest (presumably due to a residual solubility of the copolymers in EtO_2_), with just 60 ± 3 wt % of copolymers isolated after one reaction cycle.

The recovered copolymers were then reused for the same Povarov-type reactions, and the recovery-reuse cycle was repeated multiple times. P­(PC4MA-*co*-OEGMA) and P­(PC6MA-*co*-OEGMA) showed a significantly lower reaction yield after some uses. In the case of P­(PC4MA-*co*-OEGMA), the yield decreased from 85 ± 4% to 74 ± 3% after four consecutive reactions (Figure 3b). For P­(PC6MA-*co*-OEGMA), the yield dropped from 74 ± 2% to 51 ± 5% after three reactions. In contrast, P­(PC1MA-*co*-OEGMA) maintained a consistent NMR yield of ∼70% over five consecutive reaction cycles.

The high yield and stability shown by P­(PC1MA-*co*-OEGMA) were attributed to the higher integrity of the photoactive moiety in PC1 compared to PC4 and PC6, as supported by UV–vis absorption spectra of the copolymers acquired after each cycle. While clear spectral changes were observed for P­(PC4MA-*co*-OEGMA) and P­(PC6MA-*co*-OEGMA)—indicating degradation of the photocatalytic units—no significant variations were observed in the spectra of P­(PC1MA-*co*-OEGMA). This indicated that the latter system showed increased photochemical stability under the applied conditions (Figure 5c).

**Figure 5:**
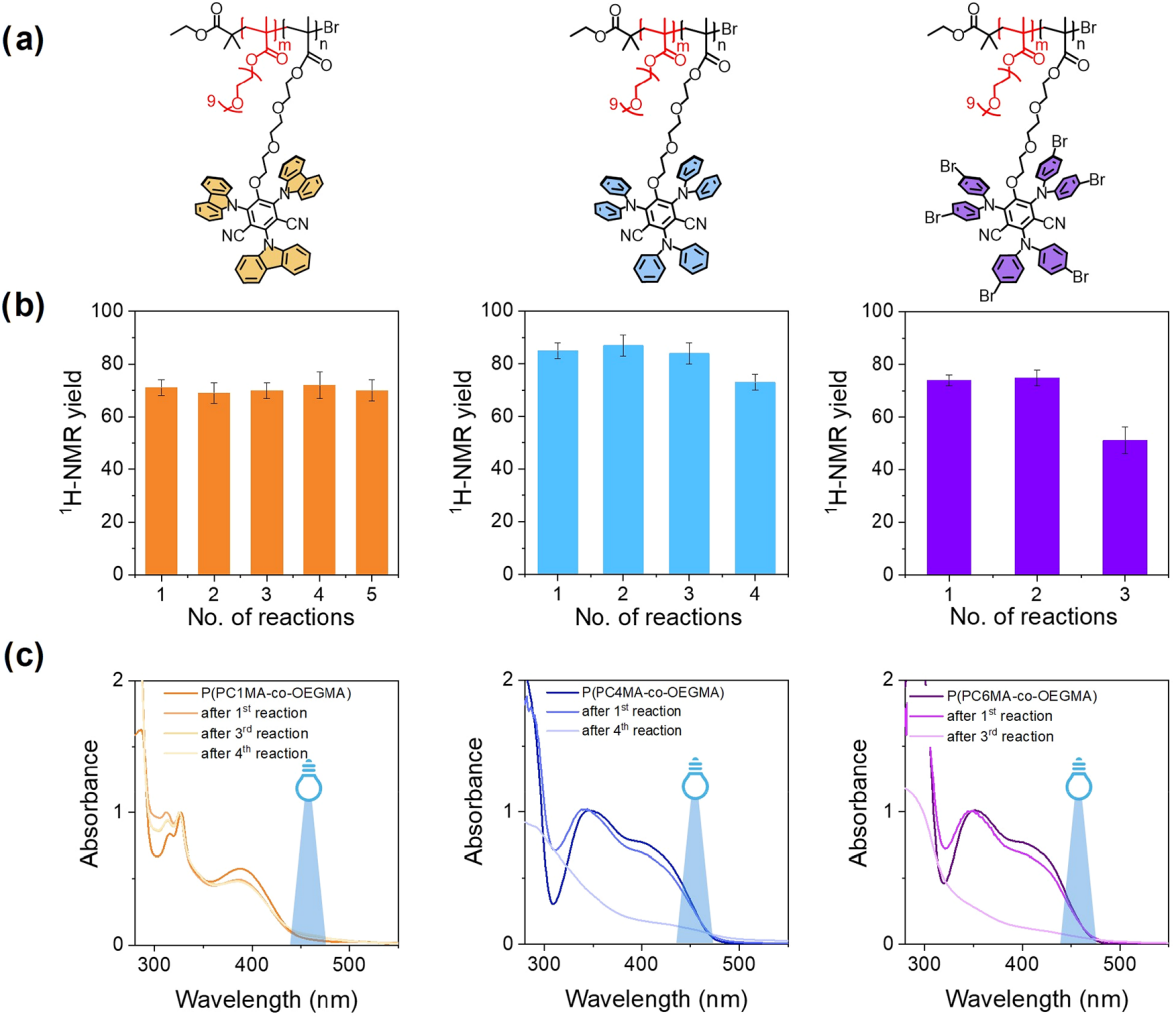
(a) Structure of P­(PC1MA-*co*-OEGMA) (left), P­(PC4MA-*co*-OEGMA) (center), and P­(PC6MA-*co*-OEGMA) (right) synthesized by ARGET ATRP. (b) ^1^H NMR yields of Povarov-type reaction between DMA and *N*-phenylmaleimide by employing P­(PC1MA-*co*-OEGMA) (left), P­(PC4MA-*co*-OEGMA) (center), and P­(PC6MA-*co*-OEGMA) (right) over several reaction cycles. (c) Absorption spectra of P­(PC1MA-*co*-OEGMA) (left), P­(PC4MA-*co*-OEGMA) (center), and P­(PC6MA-*co*-OEGMA) (right) recorded after each photoreaction.

### Polymer Brush-Supported PCs

The synthesis of NPs presenting PC-bearing copolymer brush shells was performed by surface-initiated ARGET ATRP (SI-ARGET ATRP) from ATRP initiator-functionalized SiO_*x*_ NPs (diameter ∼180 nm),^55^ while adapting the polymerization conditions previously applied for copolymerization in solution (Figure 1). We specifically focused on the generation of SiO_*x*_ NPs-P­(PC1MA-*co*-OEGMA) brushes (Scheme S3), as the corresponding copolymers in solution showed relatively high yields and the highest stability during multiple Povarov-type reactions.

The obtained core-polymer brush shell NPs were characterized by transmission electron microscopy (TEM), which revealed a uniform polymeric coating on the inorganic core (Figures 6a–c and S46), whereas dynamic light scattering (DLS) revealed that the hydrodynamic radius (*D*_h_) of core-brush shell NPs in ethanol was 380 ± 11 nm (Figure 6c).

**Figure 6:**
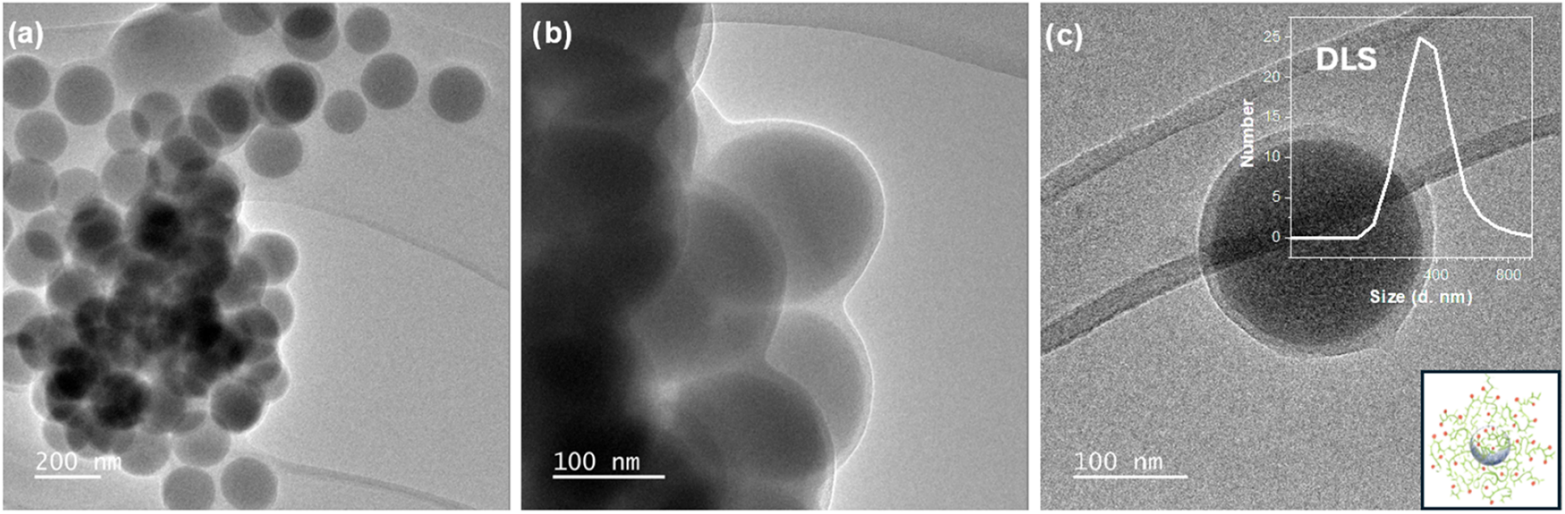
(a–c) TEM pictures displaying SiO_*x*_ NPs-P­(PC1MA-*co*-OEGMA) brushes. The inset in (c) shows a representative DLS profile recorded on dispersions of core-brush shell NPs in ethanol, highlighting their monomodal distribution in size.

The incorporation of the PC1 groups within copolymer brush shells was confirmed by emission spectroscopy—less affected than absorption spectroscopy by light scattering of NPs, which provided an estimate of 0.1 μmol of PC1 per mg of core-brush shell NPs (Figure S43).

Finally, the core–brush shell NPs were tested as supported PCs under the same reaction conditions used for the other photocatalytic systems, with a corresponding loading of 0.1 mol%. The application of SiO_*x*_ NPs-P­(PC1MA-*co*-OEGMA) brushes in the Povarov-type reaction provided a 61 ± 3% yield (Table 2, Entry 11), which is slightly lower than the yield recorded by using P­(PC1MA-*co*-OEGMA) copolymers in solution.

Core–brush shell NPs were easily recovered by centrifugation, reaching very high recovery (∼91%)—a substantial improvement compared to the recovery of the corresponding copolymers dissolved in the reaction medium. The recovered core–shell NPs were later recycled for three subsequent reactions. Each Povarov-type cycloaddition provided approximately the same yield of ∼60%, without showing any significant change in catalytic performance, whereas recovery of core–brush shell NPs remained almost quantitative at each cycle (Table 3).

**Table 3:**
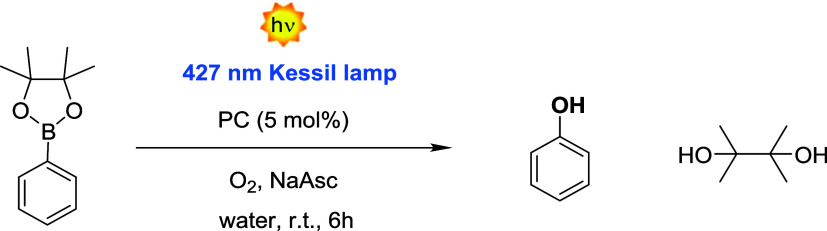
^1^H-NMR Yields in Photohydroxylation of Phenylboronic Acid Pinacol Ester^56^ in Water (Figure 1c) Using Polymer-Supported PCs

entry^a^	PC	^1^H NMR yield (%)^b^
1	P(PC1MA-*co*-OEGMA)	55
2	P(PC4MA-*co*-OEGMA)	55
3	P(PC6MA-*co*-OEGMA)	35
4	4CzIPN	9
5	4CzIPN + POEGMA	2

a Reaction conditions: phenylboronic acid pinacol ester 0.056 mmol and NaAsc (0.24 mmol) in DI water. The reaction mixture was supplied with oxygen before irradiating for 6 h with a Kessil lamp (λ_max_ = 427 nm) at 25% intensity.

b The reaction mixture was subjected to extraction with DCM, and the organic part was analyzed using 1,3,5-trimethoxybenzene as an internal standard.

### Application of PC-Bearing Copolymers in Aqueous Media

PCs included within copolymers were additionally tested in two different photoreactions in water. We initially started with a well-established reaction: the hydroxylation of phenylboronic acid pinacol ester (Figure 1c and Scheme S6). This reaction occurs via the oxidative quenching of the PC, which is mediated by the formation of the oxygen radical anion (O_2_^•–^) with the concomitant generation of the PC radical cation (Scheme S7).^56–59^ Relevantly, since the selected substrate is soluble in water, we attempted to perform this photocatalyzed reaction in an aqueous environment. When the commercially available IPN-based photocatalyst 1,2,3,5-tetrakis­(carbazol-9-yl)-4,6-dicyanobenzene (4CzIPN) was employed—one of the most commonly used IPN-based photocatalysts reported in the literature—, the yield was below 10% due to its insolubility in aqueous media (Entry 4 in Table 3). Conversely, a much higher yield of 55% was measured when employing P­(PC1MA-*co*-OEGMA) and P­(PC4MA-*co*-OEGMA) with a PC loading of 5 mol% (Table 3, Entries 1 and 2**)**. We presume that the yield could not exceed 55% due to the degradation of the starting phenylboronic acid pinacol ester. This was confirmed by ^1^H NMR of the reaction crude, which did not display the aromatic signals characteristic of the reactant (Figure S51).

Finally, we performed the same reaction within an aqueous dispersion of 4CzIPN in the presence of 20 mg of POEGMA homopolymer (entry 5 in Table 3), yielding just 2% of the product. Importantly, this result confirmed that the incorporation of the PC into the amphiphilic copolymer is essential to perform photocatalytic transformations in water. These results demonstrated the versatility of PC-bearing copolymers and highlighted how the macromolecular engineering of a copolymer support can offer the possibility for translating IPN-based photocatalysis within benign, aqueous media.

The effectiveness of P­(PCMA-*co*-OEGMA) in catalyzing photoreactions in water was additionally tested for a more challenging and less established transformation, such as the [2 + 2] photocycloaddition of 4-acetoxystyrene “in water”, which proceeds via an energy transfer (EnT) mechanism.^60^ The reaction was irradiated with blue light (λ_max_ = 427 nm, 150 mW cm^–2^) under vigorous stirring at room temperature with 3 mol% of catalyst loading. Remarkably, with P­(PC6MA-*co*-OEGMA), a 79% yield was obtained (Entry 3 in Table 4). 4-Acetoxybenzaldehyde was likewise identified as a side product, and the starting material was no longer detectable (Figure S54).

**Table 4:**
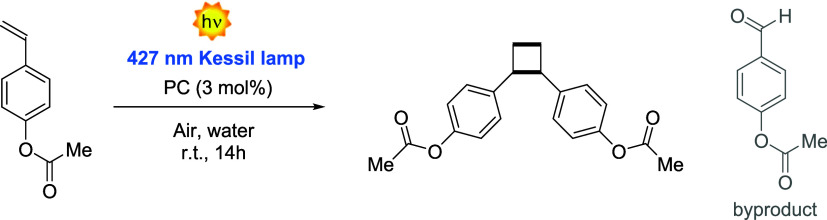
^1^H-NMR of the Photocatalyzed [2 + 2] Cycloaddition of 4-Acetoxystyrene in Water

entry^a^	PC	^1^H NMR yield (%)^b^	*trans*/*cis* ^c^	byproduct (%)^d^	selectivity (%)
1	P(PC1MA-*co*-OEGMA)	68	1:0.3	12	85
2	P(PC4MA-*co*-OEGMA)	45	1:0.4	3	93
3	P(PC6MA-*co*-OEGMA)	79	1:0.4	3	96
4	SiOx-P(PC1MA-*co*-OEGMA)^e^	65	1:0.3	12	84
5	4CzIPN	35	1:0.4	15	70
6	4CzIPN + POEGMA^f^	5	1:0.3	Nd^g^	

a Reaction conditions: 4-acetoxystyrene 0.12 mmol in DI water with P­(PCMA-*co*-OEGMA) corresponding to 3 mol % loading of PC. Irradiation for 14 h with a Kessil lamp (λ_max_ = 427 nm) at 50% intensity in an open-to-air vial.

b The polymer was selectively precipitated with Et_2_O. The crude without polymer was analyzed using 1,3,5-trimethoxybenzene as an internal standard.

c Determined by ^1^H NMR.

d The ^1^H NMR yield of the byproduct was calculated considering the integral of the diagnostic aldehyde proton at 10 ppm.

e Catalyst loading at 1 mol % corresponding to 30 mg of particles synthesized with a 15% PC1MA comonomer.

f 3 mol % of 4CzIPN loading and 20 mg of POEGMA homopolymer.

g Not detected.

Similarly to what was observed for the hydroxylation reaction, when 4CzIPN alone or when the POEGMA homopolymer was simply mixed with 4CzIPN, a significant drop in yield was recorded (entries 5–6, Table 4). These results further confirmed how copolymerization between OEGMA and PC1MA is essential for guaranteeing efficient photocatalysis.

It is also relevant to mention that, although the available physicochemical parameters do not reveal a straightforward trend comparing the different PCs, the highest efficiency observed for PC6 could point toward a heavy-atom effect. This reactivity profile would further support an EnT mechanism.^61–65^

## Conclusions

The molecular engineering of polymeric materials for supporting IPN-based PCs enables performing challenging photocatalyzed organic transformations while ensuring PCs’ recyclability and compatibility with benign reaction conditions.

This is achieved by first synthesizing a library of IPN-based PCs featuring carbazole- or diphenylamine-derived chromophores and by analyzing and rationally tuning their photophysical and redox properties through a systematic variation of the substituents on the IPN core. The PCs that were more efficient in catalyzing model reactions were modified with methacrylate functions to enable their incorporation within polymeric materials by exploiting ARGET ATRP.

While testing a Povarov-type cycloaddition, PC-bearing copolymers provided high yields across a range of loadings, ensuring high photocatalytic performance even at relatively low concentrations (up to 0.1 mol% loading). The amphiphilic nature of PC-copolymers further allowed us to circumvent the poor solubility of PC moieties in water, enabling the successful realization of diverse photoredox-catalyzed organic transformations as well as [2 + 2] energy transfer processes under benign aqueous conditions in the absence of surfactants.

At the same time, the intrinsic chemical fragility of donor–acceptor cyanoarene PCs under prolonged irradiation remains an important aspect to consider for future developments. Notably, comprehensively elucidating degradation pathways under catalytic turnover is inherently challenging as multiple competing photophysical and photochemical processes may operate simultaneously. In this context, the integration of IPN-based PCs within tailored polymer architectures provides an effective strategy to enhance their stability and practical applicability while enabling improved recovery and reuse.

Simultaneously, copolymer brushes featuring PC moieties grafted from SiO*_x_* NPs were demonstrated to maintain photocatalytic activity while guaranteeing nearly quantitative PC recovery over multiple reaction cycles, overcoming the well-known fragility of IPN PCs under photocatalytic conditions.

In summary, this study demonstrates that the rational design of polymer-supported photoredox catalysts represents a powerful strategy for generating photocatalytic systems that are fully recyclable and compatible with more sustainable aqueous conditions.

## Supplementary Material



## Notes

Materials and methods, SEC elugrams, NMR spectra, absorption and emission fluorescence spectra, cyclic voltammograms, fluorescence lifetime measurements (PDF)
