# Supplementary material for: Recyclable IPN Photocatalysts Supported by Polymer Matrices: From Soluble Copolymers to Core-Polymer Brush Shell Nanostructures
Source: J Am Chem Soc. 2026 Feb 11;148(7):7846–56. doi: 10.1021/jacs.5c22227 (PMC12951429; doi:10.1021/jacs.5c22227)
Supplement: Supplementary file 1 [file ja5c22227_si_001.pdf]

Supporting information

Recyclable IPN Photocatalysts Supported by Polymer Matrices: From Soluble Copolymers to Core-Polymer Brush Shell Nanostructures

Elena Avanzini,[1] Alessio Lo Bocchiaro,[1] Agata Checcozzo,[2] Luis Izquierdo Aranda,[2] Eric Ruzicka,[1] Jorge Humbrias Martin,[2] Gianluca Gazzola,[1] Francesca Lorandi,[1] Luca Dell'Amico,[2*] Edmondo M. Benetti[1*]*

[1] Laboratory for Macromolecular and Organic Chemistry, Department of Chemical Sciences, University of Padova, Via Marzolo 1, 35131 Padova, Italy.

[2] Sustainable Synthesis and Catalysis, Department of Chemical Sciences, University of Padova, Via Marzolo 1, 35131 Padova, Italy.

Table fo contents

MATERIALS AND METHODS 4
Photochemical reaction setup 5
PROCEDURES FOR THE SYNTHESIS OF PCs 6
Synthesis of PC1 6
Synthesis of PC2 7
Synthesis of PC3 9
Synthesis of PC4 10
Synthesis of PC5 12
Synthesis of PC6 14
PC-monomers synthesis 15
Synthesis of poly(PCMA-*co*-OEGMA) 17
P(PC1MA-*co*-OEGMA) 18
P(PC4MA-*co*-OEGMA) 19
P(PC6MA-*co*-OEGMA) 20
Synthesis of SiO_2-P(PC1MA-*co*-OEGMA) 21
Grafting density 22
Photoreactions 23
Povarov-type photoreaction 23
Reported mechanism of Povarov-type photoreaction 24
Photohydroxylation in water 24
Reported mechanism of the photohydroxylation 25
Photocatalyzed [2+2] cycloaddition in water 25
Photophysical studies 26
Absorption and emission measurement 26
Redox potentials measurement 26
PC1 (2,4,6, Tris(3,6-carbazol-9-yl) 5-triethylenglycol isophthalonitrile) 27
PC2 (2,4,6 Tris(3,6-dimethoxy-carbazol-9-yl)-5-tryethylenglicolisobutyrate isophthalonitrile) 28
PC3 (2,4,6, Tris(3,6-dibromo-carbazol-9-yl) 5-triethylenglycol isophthalonitrile) 30
PC4 (2,4,6 Tris(diphenylamino)-5-tryethylenglicolisobutyrate isophthalonitrile) 31
PC5 (2,4,6 Tris(4-4'dimethoxydiphenylamino)-5-tryethylenglicolisobutyrate isophthalonitrile) 33
PC6 (2,4,6 Tris(4-4'dibromodiphenylamino)-5-tryethylenglicolisobutyrate isophthalonitrile) 34
Polymers redox potential summary 36

P(PC1MA-*co*-OEGMA) 37
P(PC4MA-*co*-OEGMA) 39
P(PC6MA-*co*-OEGMA) 41
Lifetimes measurements 42
Lifetimes summary at room temperature in ACN 43
PC1 lifetimes in ACN 44
PC2 lifetimes in ACN 44
PC3 lifetimes in ACN 45
PC4 lifetimes in ACN 45
PC5 lifetimes in ACN 46
PC6 lifetimes in ACN 46
P(PC1MA-*co*-OEGMA) lifetimes in ACN 47
P(PC4MA-*co*-OEGMA) lifetimes in ACN 47
P(PC6MA-*co*-OEGMA) lifetimes in ACN 48
SiO_x-P(PC1MA-*co*-OEGMA) lifetimes in ACN 48
Lifetimes summary in MeTHF 49
PC1 lifetimes in MeTHF 49
PC2 lifetimes in MeTHF 50
PC3 lifetimes in MeTHF 50
PC4 lifetimes in MeTHF 50
PC5 lifetimes in MeTHF 51
PC6 lifetimes in MeTHF 52
Absorption and emission calibrations 53
PC1 absorption calibration 53
P(PC1MA-*co*-OEGMA) emission calibration 53
PC4 absorption calibration 54
PC6 absorption calibration 54
Transmission electron microscopy (TEM) pictures 55
NMR of photoreaction's crudes and purified products 56
Povarov-type photoreaction 56
Photo hydroxylation in water of phenyl boronic acid pinacol ester 57
[2+2] cycloaddition of 4-acetoxystyrene 59
Kinetic incorporation of PCMA 61

MATERIALS AND METHODS

Materials: Commercial grade reagents and solvents were used as received, unless otherwise stated. Specifically carbazole (≥ 95%), 3,6 dibromocarbazole (97%), diphenylamine (≥ 99%), bis(4-bromophenylamine) (97%), 4,4'-dimethoxydiphenylamine (99%), triethylen glycol (TEG, 99%), 2,2',6,6'-tetramethylpiperidine (TMP, ≥ 99%), sodium hydride (60%), triethylamine (NEt_3, ≥ 99.5%), methacryloyl chloride (97%), isobutyryl chloride (98%), oligo[(ethylene glycol) methyl ether methacrylate] (OEGMA) with M_n ~500 g mol^{-1}, 3-aminopropyltriethoxysilane (APTES, 99%), ethyl α-bromoisobutyrate (EBiB, 98%), α-bromoisobutyryl bromide (BiBB, 98%), $CuBr_2$ (99%), tris(2-pyridylmethyl)amine (TPMA, 98%), Sodium ascorbate (NaAsc, ≥ 98%), phenylboronic acid pinacol ester (97%), N-phenylmaleimide (97%) and N,N-dimethylaniline (99%) were purchased from Sigma Aldrich. While tetrafluoroisophtalonitrile (≥ 97%) and 3,6 dimethoxycarbazole (97%) were purchased from FluoroChem.

OEGMA with M_n ~500 g mol^{-1} was purified by passing through a basic aluminium oxide column to remove the inhibitor before use.

MilliQ water was purified with a Millipore Direct-Q 5 ultrapure water system or a Milli-Q® IQ 7003 purification system.

Non-porous, monodisperse silica particles of ~200 nm diameter were purchased from Sigma Aldrich.

NMR spectra were recorded on Bruker AVANCE Neo 400 Nanobay equipped with a BBFOATM-z grad probehead. The chemical shifts (δ) for ^{1}H and ^{13}C are given in ppm relative to residual signals of the solvents ($CHCl_3$ @ 7.26 ppm ^{1}H NMR, 77.16 ppm ^{13}C NMR). Coupling constants are given in Hz. The following abbreviations are used to indicate the multiplicity: s, singlet; d, doublet; t, triplet; q, quartet; m, multiplet; bs, broad signal. NMR yields were calculated by using 1,3,5-trimethoxybenzene as internal standard.

Chromatographic purification of products during the synthesis of the PCs was accomplished using flash chromatography on silica gel (SiO_2, 0.04-0.063 mm) purchased from Merck, with the indicated solvent system. Thin-layer chromatography (TLC) analysis was performed on pre-coated Merck TLC plates (silica gel 60 GF254). Visualization of the developed chromatography was performed by checking UV absorbance (at λ = 254 nm) as well as with aqueous potassium permanganate solutions. Organic solutions were concentrated under reduced pressure on a Büchi rotary evaporator.

Steady-state absorption spectroscopy studies were performed at room temperature on a Varian Cary 50 UV-vis double beam spectrophotometer; 10-mm and 1-mm path length Hellma Analytics 100 QS quartz cuvettes were

used. Steady-state fluorescence spectra for lifetime studies were recorded on a Varian Cary Eclipse Fluorescence spectrophotometer using 10-mm path length Hellma Analytics 117.100F QS quartz cuvettes.

Size exclusion chromatography (SEC) was performed using an Agilent Viscotek 302-TDA instrument equipped with a refractive index (RI) detector, a GRAM pre-colum (50x8 mm) and two GRAM analytical linear columns (300x8 mm) from Agilent PSS connected in series to determine the number average molecular weight (M_n) and dispersity ($Đ$) of polymers. The column compartment and RI detector were both at 60 °C, and the eluent used was DMF containing 10 mM LiBr at a flow rate of 1 mL/min. Every sample (polymer concentration ~2 mg/mL) was prepared by filtering through neutral alumina over a PTFE membrane with a porosity of 0.20 μm. The column system was calibrated with 12 linear poly(methyl methacrylate) standards (M_n = 540–2,210,000 Da).

Purification of the polymers was done using 3.5 kDa Spectra/Por® dialysis membranes.

The potentiostat used for the calculation of the redox potentials is VIONIC powered by INTELLO software from Metrohm Autolab. The measurements were done in a 3-electrode cell. Platinum wire (99.9%) ST 0.6/40 mm was used as auxiliary electrode. The working electrode was a glassy carbon, 70 mm long and 2 mm diameter (body diameter 6 mm), and an Ag/Ag^+ non-aqueous refillable reference electrode (6 mm diameter).

The morphology and microstructure of the samples were characterized by transmission electron microscopy (TEM) and high-angle annular dark-field (HAADF) scanning transmission electron microscopy (HAADF-STEM) using TEM JEOL F200.

Thermogravimetric analysis (TGA) was carried out using SDT 650 by TA Instrument that exploits a horizontal dual-beam thermobalance and it is equipped with TRIOS software. The temperature was scanned from 200 °C with a ramp 10.00 °C/min to 1000.00 °C.

Photochemical reaction setup

Figure S1 shows the general setup of a batch reaction performed under a 456-nm Kessil Lamp. The reaction mixture was placed at a fixed distance (about 3 cm) from the light source set at 50% power and stirred under illumination for the indicated time. Three fans were used to maintain constant the temperature (25±2 °C) which was controlled by a thermometer.

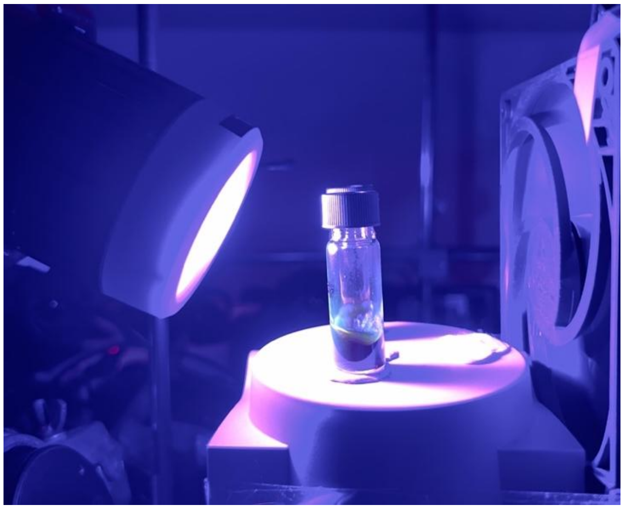

Figure S1. Photoreaction setup using a Kessil lamp with λ_{max}= 456 nm at 50% intensity, placed at 3-cm distance from the vial.

PROCEDURES FOR THE SYNTHESIS OF PCs

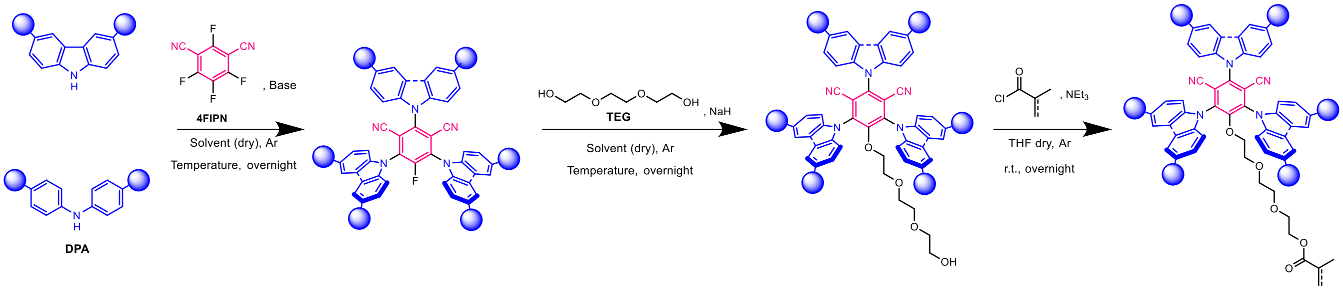

Scheme S1. general procedure for the synthesis of PCs and monomers.

Synthesis of PC1

Synthesis of 2,4,6, Tris(3,6-carbazol-9-yl) 5-fluoroisophthalonitrile (PC1F)

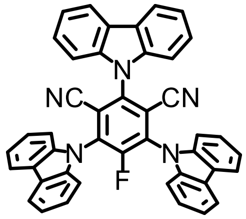

3CzFIPN was synthesized starting from tetrafluoroisophtalonitrile (1 eq, 5 mmol, 1g), tetramethyl piperidine (4.5 eq, 22.5 mmol, 3,8 mL) and carbazole (3 eq, 15 mmol, 2.5g). The reagents were added in 30 mL of acetonitrile and let 24h at 80 °C under reflux. The solvent of the crude product solution was removed by rotary evaporation. The product was isolated by flash chromatography (gradient DCM:EtOAc 4:1, 7:3, 1:1). The isolated product with a yield of 80% was characterized with ^{1}H-NMR, ^{13}C-NMR, ^{19}F-NMR.

^{1}H-NMR (400 MHz, $CDCl_3$): δ (ppm) 8.31-8.24 (m, 6H); 7.75 (d, *J*= 8.1 Hz, 2H); 7.70-7.61 (m, 10H), 7.50-7.42 (m, 6H). **^{13}C-NMR (100 MHz, $CDCl_3$):** δ (ppm) 154.4, 142.8, 140.2, 139.4, 135.5, 127.2, 126.98, 127.0, 124.3, 124.0, 122.3, 122.1, 121.0, 120.2, 117.4, 110.8, 110.3. **^{19}F-NMR (376 MHz, CD_3CN):** δ (ppm) -116.1 (s, 1F)

Synthesis of 2,4,6, Tris(3,6-carbazol-9-yl) 5-triethylenglycol isophthalonitrile (PC1TEG)

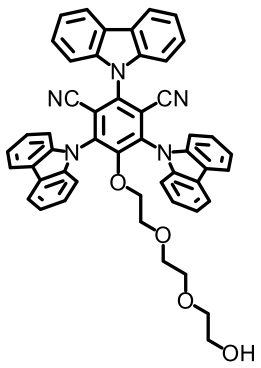

For the second step, NaH 60% (1.5 eq, 5.8 mmol, 232 effective mg) was added and 3 cycles Ar/vacuum performed. Under inert atmosphere, 130 mL of dry THF were put in the flask and then triethylene glycol (1.5 eq, 5.8 mmol, 0.78 mL) and PC1F (1 eq, 3.9 mmol, 2.5 g) were added. After 18h the solvent was evaporated and the product isolated by flash chromatography (gradient DCM, DCM:EtOAc 9:1 and DCM:EtOAc 8:2). It was then characterized with ^{1}H-NMR and ^{13}C-NMR.

^{1}H-NMR (400 MHz, $CDCl_3$): δ (ppm) 8.31-8.24 (m, 6H); 7.75 (d, *J*= 8.0 Hz, 2H); 7.70-7.61 (m, 10H), 7.50-7.42 (m, 6H), 3.41 (q, *J*= 4.1 Hz, 2 H), 3.17 (t, *J*= 4.0 Hz, 2H), 3.05 (t, *J*= 4.0 Hz, 2H), 2.81 (t, *J*= 4.0 Hz, 2H), 2.48 (t, *J*= 4.0 Hz, 2H), 2.44 (t, *J*= 4.0 Hz, 2H). **^{13}C NMR (100 MHz, $CDCl_3$):** δ (ppm) 155.1, 140.3, 139.7, 138.9, 126.8, 121.2, 121.9, 120.9, 116.5, 110.2, 109.3, 74.0, 72.0, 69.8, 69.7, 69.2, 61.6.

Synthesis of 2,4,6, Tris(3,6-carbazol-9-yl) 5-triethylenglycol isophthalonitrile (PC1)

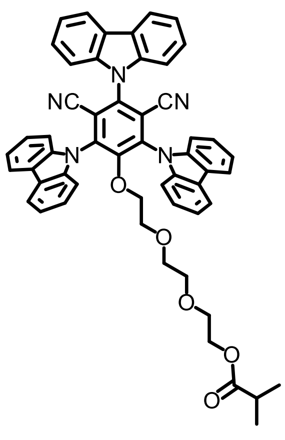

Under inert atmosphere PC1TEG (1 eq, 0.3 mmol, 234 mg) was dissolved in 2 mL of dry THF. Then, triethylamine (1.2 eq, 0.36 mmol, 50 µL) was added dropwise and finally isobutyryl chloride (1.2 equiv., 0.36 mmol, 38 µL). The reaction was stopped after 24h and extracted (adding DCM) with HCl 1M. After the extraction the solvent was evaporated and the crude purified with flash chromatography (gradient DCM, DCM:EtOAc 9:1 and DCM:EtOAc 8:2) giving an isolated yield of about 55%. The pure compound was characterized by ^{1}H-NMR, ^{13}C-NMR, lifetime measurements, absorption and emission spectra. This compound was specifically used for the calculation of the redox potentials with cyclic voltammetry.

^{1}H-NMR (400 MHz, $CDCl_3$): δ (ppm) 8.31-8.24 (m, 6H); 7.75 (d, *J*= 8.0 Hz, 2H); 7.70-7.61 (m, 10H), 7.50-7.42 (m, 6H), 4.04 (t, *J*= 4.0 Hz, 2H), 3.34 (t, *J*= 4.0 Hz, 2H), 2.98 (t, *J*= 4.0 Hz, 2H), 2.86 (t, *J*= 4.0 Hz, 2H), 2.49 (m, 5H), 1.1 (d, *J*=4.0 Hz, 6H). **^{13}C-NMR (100 MHz, $CDCl_3$):** δ (ppm) 166.1, 155.3, 140.2, 139.6, 139.0, 126.8, 124.5, 122.0, 121.2, 120.1, 116.4, 110.2, 109.3, 74.0, 70.0, 69.7, 69.1, 68.7, 63.3, 33.9, 18.9.

Synthesis of PC2

Synthesis of 2,4,6, Tris(3,6-dimethoxy-carbazol-9-yl) 5-fluoroisophthalonitrile (PC2F)

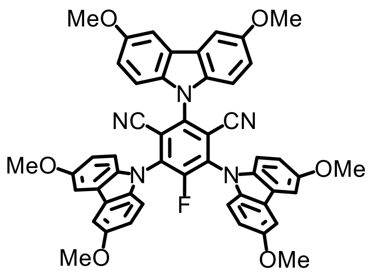

In an oven-dried Schlenk flask equipped with a magnetic stir bar, 3.0 equiv of 3,6-dimethoxy-9H-carbazole (3408.9 mg, 15 mmol) and 1.0 equiv of tetrafluoroisophthalonitrile (1000.5 mg, 5 mmol) were dissolved in dry MeCN (0.08 M, relating to the corresponding tetrafluoroisophthalonitrile) under an atmosphere of argon. Tetramethyl piperidine (4.5 equiv, 3.8 mL) was added slowly. The reaction mixture was heated at 80 °C and left at reflux overnight. Finally, the solution was recrystallized with H_2O/MeCN to have the product as precipitate. 2 g of product was obtained (50% yield), red solid.

^{1}H NMR (400 MHz, $CDCl_3$): δ (ppm) 7.57 (dd, J = 19.3, 2.4 Hz, 6H), 7.36-7.08 (m, 12H), 3.98 (d, J = 10.0 Hz, 18H). **^{13}C-NMR** (100 MHz, $CDCl_3$): δ (ppm) 155.8, 155.5, 134.5, 125.7, 125.4, 115.8, 115.5, 111.0, 110.2, 104.1, 103.9, 56.1.**^{19}F-NMR decoupled ^{1}H** (376 MHz, $CDCl_3$): δ (ppm) -112.6 (s, 1F).

Synthesis of 2,4,6, Tris(3,6-dimethoxy-carbazol-9-yl) 5-triethylenglycol isophthalonitrile (PC2TEG)

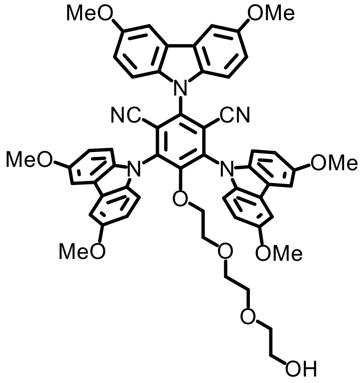

In an oven-dried Schlenk flask equipped with a magnetic stir bar, 1.0 equiv of monofluorinated intermediate PC2F (1400 mg, 1.7 mmol), with carbazole as donor moieties, and 1.2 equiv of NaH (60% in oil) were dissolved in dry THF (0.2 M, relating to PC2F) under an atmosphere of argon. Then, 3.5 equiv of triethylene glycol were added to the solution. The resulting reaction mixture was stirred overnight at room temperature. The suspension was quenched by the addition of water, washed with brine and extracted with DCM. The organic phase was dried over Na_2SO_4, and the solvent was removed under reduced pressure. The crude product was purified by column chromatography on silica gel with DCM/EtOAc, 10-70% EtOAc as gradient. 829 mg of product was obtained (51% yield), red solid.

^{1}H NMR (400 MHz, $CDCl_3$): δ (ppm) 7.57 (dd, J = 19.3, 2.4 Hz, 6H), 7.33-7.24 (m, 4H), 7.19-7.15 (m, 6H), 3.97 (s, 18H), 3.60-3.58 (m, 2H), 3.38-3.31 (m, 2H), 2.98-2.95 (m, 4H), 2.57-2.54 (m, 4H).**^{13}C-NMR** (100 MHz, $CDCl_3$): δ (ppm) 155.4, 155.3, 155.3, 154.0, 141.1, 139.3, 135.8, 134.9, 125.2, 125.1, 115.6, 115.4, 115.1, 111.7, 111.3, 110.3, 103.9, 103.6, 103.6, 73.9, 69.9, 69.7, 69.2, 68.9, 63.1, 56.1.

Syntesis of 2,4,6 Tris(3,6-dimethoxy-carbazol-9-yl)-5-tryethylenglicolisobutyrate isophthalonitrile (PC2)

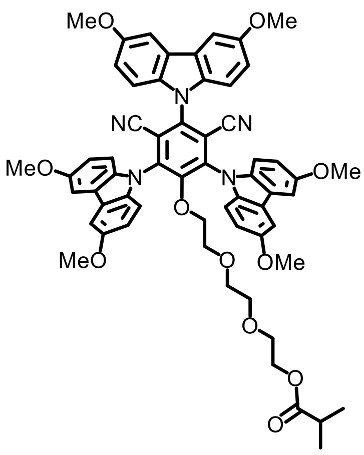

In an oven-dried Schlenk flask equipped with a magnetic stir bar, 1.0 equiv of PC2TEG (75.0 mg, 0.08 mmol), and 1.5 equiv of isobutyryl chloride (0.12 mmol, 9 μL) were dissolved in dry THF (0.15 M, 0.53 mL) under an atmosphere of argon. Then, 1.6 equiv (13 μL) of triethylamine were added slowly at the solution. The resulting reaction mixture was stirred overnight at room temperature. The suspension was quenched by the addition of water, washed with brine and extracted with DCM. The organic phase was dried over Na_2SO_4, and the solvent was removed under reduced pressure. The crude product was purified by column chromatography on silica gel using DCM/EtOAc, 10-50% EtOAc as gradient. 30 mg of product was obtained (37% yield), red solid.

^{1}H NMR (400 MHz, $CDCl_3$): δ (ppm) 7.57 (dd, *J* = 19.3, 2.4 Hz, 6H), 7.33-7.24 (m, 4H), 7.19-7.15 (m, 6H), 4.08 (t, *J*= 4.0 Hz, 2H), 3.97 (m, 18H), 3.39 (t, *J*= 4.0 Hz, 2H), 2.98-2.91 (m, 4H), 2.57 (m, 2H), 2.56 (m, 2H), 2.51 (hept, 1H), 1.12 (d, *J* = 7.0 Hz, 6H). **^{13}C-NMR** (100 MHz, $CDCl_3$): δ (ppm) 155.4, 154.1, 141.0, 139.3, 135.8, 134.9, 125.2, 115.7, 115.0, 111.7, 111.3, 110.3, 103.9, 103.6, 73.9, 69.9, 69.7, 69.2, 68.9, 63.1, 56.1, 33.8, 18.9.

Synthesis of PC3

Synthesis of 2,4,6, Tris(3,6-dibromo-carbazol-9-yl) 5-fluoroisophthalonitrile (PC3F)

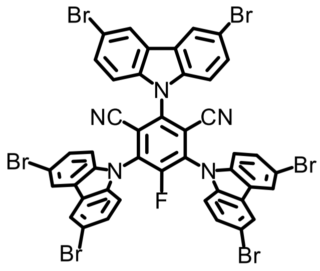

In an oven-dried Schlenk flask equipped with a magnetic stir bar, 3.0 equiv (4875.0 mg, 15 mmol) of 3,6-dibromo-9H-carbazole and 1.0 equiv of tetrafluoroisophthalonitrile (1000.5 mg, 5 mmol) were dissolved in dry MeCN (0.08 M, 62.5 mL) under an atmosphere of argon. Tetramethyl piperidine (4.5 equiv, 3.83 mL) was added slowly. The reaction mixture was heated at 80 °C and left at reflux overnight. Finally, the product was precipitated during the reaction. It was washed with cooled MeCN and dried with vacuum pump. 4659 mg of product was obtained (84% yield), yellow solid.

^{1}H NMR (400 MHz, $CDCl_3$): δ (ppm) 8.28 (dd, *J* = 17.2, 1.9 Hz, 6H), 7.72 (ddd, *J* = 17.2, 8.7, 1.9 Hz, 6H), 7.19 (dd, *J* = 17.2, 7.9 Hz, 6H). **^{13}C-NMR** (100 MHz, $CDCl_3$): δ (ppm) 138.9, 138.0, 130.7, 130.6, 125.6, 125.4, 124.6, 124.4, 116.3, 116.0, 111.3, 110.6. **^{19}F-NMR decoupled ^{1}H** (376 MHz, $CDCl_3$): δ (ppm) -110.3 (s, 1F).

Synthesis of 2,4,6, Tris(3,6-dibromo-carbazol-9-yl) 5-triethylenglycol isophthalonitrile (PC3TEG)

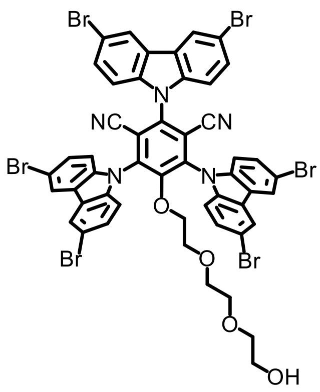

In an oven-dried Schlenk flask equipped with a magnetic stir bar, 1.0 equiv of PC3F (2000.0 mg, 1.8 mmol), with carbazole as donor moieties, and 1.2 equiv of NaH 60% in oil (86.0 mg) were dissolved in dry THF (0.2 M, 9 mL) under an atmosphere of argon. Then, 3.5 equiv of triethylene glycol (0.85 mL, 6.3 mmol) were added to the solution. The resulting reaction mixture was stirred overnight at room temperature. The suspension was quenched by the addition of water, washed with brine and extracted with DCM. The organic phase was dried over Na_2SO_4, and the solvent was removed under reduced pressure. The crude product was purified by column chromatography on silica gel with DCM/EtOAc, 10-50% EtOAc as gradient. 611 mg of product was obtained (30% yield), yellow solid.

^{1}H NMR (400 MHz, $CDCl_3$): δ (ppm) 8.26 (dd, *J* = 17.2, 1.9 Hz, 6H), 7.70 (ddd, *J* = 17.2, 8.7, 1.9 Hz, 6H), 7.29-7.24 (m, 6H), 3.65 (bs, 2H), 3.45 (t, *J* = 4.0 Hz, 2H), 3.07 (t, *J* = 4.0 Hz, 2H), 3.01 (t, *J* = 4.0 Hz, 2H), 2.69-2.59 (m, 4H). **^{13}C-NMR** (100 MHz, $CDCl_3$): δ (ppm) 154.9, 139.4, 138.3, 130.5, 125.2, 124.4, 116.2, 115.6, 112.0, 110.9, 74.2, 72.2, 70.2, 69.7, 61.6, 60.4.

Synthesis of 2,4,6 Tris(3,6-dibromo-carbazol-9-yl)-5-tryethylenglicolisobutyrate isophthalonitrile (PC3)

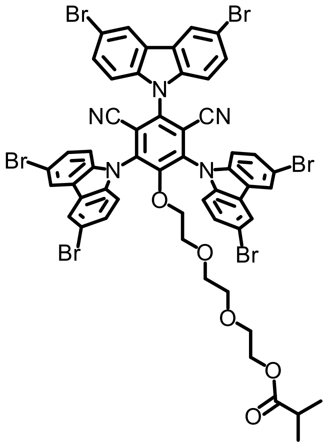

In an oven-dried Schlenk flask equipped with a magnetic stir bar, 1.0 equiv of PC3TEG (148.6 mg, 0.12 mmol) and 1.5 equiv of isobutyryl chloride (20 μL , 0.18 mmol) were dissolved in dry THF (0.15 M, 0.8 mL) under an atmosphere of argon. Then, 1.6 equiv of triethylamine (0.19 mmol, 14 μL) were added slowly to the solution. The resulting reaction mixture was stirred overnight at room temperature. The suspension was quenched by the addition of water, washed with brine and extracted with DCM. The organic phase was dried over Na_2SO_4, and the solvent was removed under reduced pressure. The crude product was purified by column chromatography on silica gel with DCM/EtOAc, 10-30% EtOAc as gradient. 60.6 mg of product was obtained (42% yield), yellow solid.

^{1}H NMR (400 MHz, $CDCl_3$): δ (ppm) 8.26 (dd, *J* = 10.6, 1.9 Hz, 6H), 7.70 (ddd, *J* = 8.6, 2.0, 0.7 Hz, 6H), 7.21 (dd, *J* = 8.7, 2.2 Hz, 6H). 4.13 (t, *J*= 4.0 Hz, 2H), 3.48 (t, *J*= 4.0 Hz, 2H), 3.01-2.98 (m, 4H), 2.67-2.61 (m, 4H), 2.50 (hept, *J* = 7.0 Hz, 1H), 1.10 (d, *J* = 7.0 Hz, 6H). **^{13}C-NMR** (100 MHz, $CDCl_3$): δ (ppm) 178.2, 155.0, 139.3, 139.1, 138.3, 138.2, 130.5, 130.4, 125.2, 124.4, 124.1, 116.1, 115.6, 111.8, 110.7, 74.1, 69.9, 69.4, 69.1, 62.9, 33.9, 19.0.

Synthesis of PC4

Synthesis of 2,4,6 Tris(diphenylamino)-5-fluoroisophthalonitrile (PC4F)

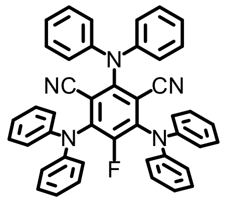

In an oven-dried Schlenk flask equipped with a magnetic stir bar, 1.25 equiv diphenylamine (634.6 mg, 1.25 mmol) was dissolved in dry THF (0.05 M, 20 mL) under an atmosphere of argon. Then, 1.88 equiv NaH 60% in oil (225.6 mg) was added and the suspension was stirred at 50 °C for 30 minutes. Finally, 1.0 equiv of tetrafluoroisophthalonitrile was added (1 mmol, 200 mg) and the resulting mixture was stirred at room temperature for 24h. The reaction mixture was quenched by the addition of water. After removal of THF, the residue was dissolved in DCM and washed with water. The organic phase was dried over Na_2SO_4, and the solvent was removed under reduced pressure. The crude product was purified by column chromatography on silica gel with hexanes/DCM, 12-85% DCM as gradient. 516.5 mg of product was obtained (79% yield), yellow solid.

^{1}H NMR (400 MHz, $CDCl_3$): δ (ppm) 7.27-7.24 (m, 12H), 7.08-7.03 (m, 6H), 7.01-6.97 (m, 12H). According to literature. **^{13}C-NMR** (100 MHz, $CDCl_3$): δ (ppm) 152.4 (d, $^1J_{C,F}$= 257.9 Hz, *C*F), 151.8, 145.5, 145.3, 143.1, 129.4, 129.3, 124.6, 124.0, 122.7, 122.68, 112.6, 109.0. According to literature. **^{19}F-NMR decoupled ^{1}H** (376 MHz, $CDCl_3$): δ (ppm) -121.3 (s, 1F).

Synthesis of 2,4,6 Tris(diphenylamino)-5-triethylenglycol isophthalonitrile (PC4TEG)

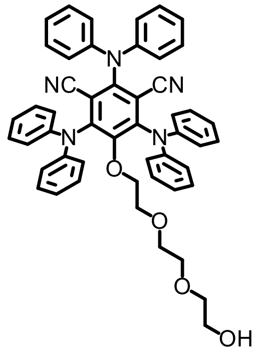

In an oven-dried Schlenk flask equipped with a magnetic stir bar, 1.0 equiv of PC3F (0.5 mmol, 323.9 mg) and 1.2 equiv of NaH 60% in oil (24.0 mg) were dissolved in dry DMAc (0.2 M, 5 mL) under an atmosphere of argon. Then, 3.5 equiv of triethylene glycol (1.75 mmol, 230 μL) were added at the solution. The resulting reaction mixture was heated at 100 °C and left at reflux overnight. The suspension was quenched by the addition of water, extracted with DCM and washed with LiCl (5% p/V). The organic phase was dried over Na_2SO_4, and the solvent was removed under reduced pressure. The crude product was purified by column chromatography on silica gel with DCM/EtOAc, 10-50% EtOAc as gradient. 232.0 mg of product was obtained (58% yield), yellow solid.

^{1}H NMR (400 MHz, $CDCl_3$): δ (ppm) 7.28-7.26 (m, 12H), 7.05-6.95 (m, 18H), 3.73 (t, *J*=4.0 Hz, 2H), 3.64 (bs, 2H), 3.46 (t, *J*=4.0 Hz, 2H), 3.32 (t, *J*=4.0 Hz, 2H), 3.01 (t, *J*=4.0 Hz, 2H), 2.87 (t, *J*=4.0 Hz, 2H). **^{13}C-NMR** (100 MHz, $CDCl_3$): δ (ppm) 151.5, 150.8, 148.5, 145.7, 145.5, 129.4, 124.8, 124.0, 123.9, 123.1, 122.7, 122.3, 113.1, 111.2, 72.5, 72.4, 70.5, 70.3, 68.8, 61.8

Synthesis of 2,4,6 Tris(diphenylamino)-5-tryethylenglicolisobutyrate isophthalonitrile (PC4)

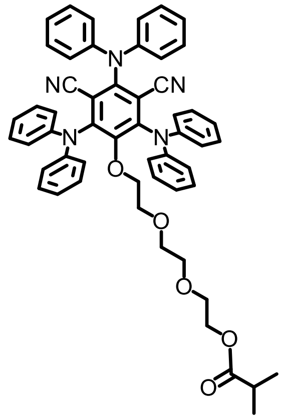

In an oven-dried Schlenk flask equipped with a magnetic stir bar, 1.0 equiv of PC3TEG (0.1 mmol, 86.5 mg) and 1.5 equiv of isobutyryl chloride (0.15 mmol, 12 µL) were dissolved in dry THF (0.15 M, 0.7 mL) under an atmosphere of argon. Then, 1.6 equiv of triethylamine (0.16 mmol, 16 µL) were added slowly to the solution. The resulting reaction mixture was stirred overnight at room temperature. The suspension was quenched by the addition of water, washed with brine and extracted with DCM. The organic phase was dried over Na_2SO_4,, and the solvent was removed under reduced pressure. The crude product was purified by column chromatography silica gel DCM/EtOAc, 10-30% EtOAc as gradient. 23.0 mg of product obtained (27% yield), yellow solid.

^{1}H NMR (400 MHz, $CDCl_3$): δ (ppm) 7.30-7.23 (m, 12H), 7.08-6.98 (m, 18H), 4.19-4.15 (m, 2H), 3.74 (t, J = 5.5 Hz, 2H), 3.56 (t, J = 4.1 Hz, 2H), 3.33 (t, J = 4.1 Hz, 2H), 3.06 – 3.01 (m, 2H), 2.88 (t, J = 5.5 Hz, 2H), 2.56 (hept, J = 7.0 Hz, 1H), 1.17 (d, J = 7.0 Hz, 6H). **^{13}C-NMR** (100 MHz, $CDCl_3$): δ (ppm) 177.2, 151.5, 150.9, 148.5, 145.7, 145.6, 129.4, 129.4, 124.1, 123.9, 122.7, 122.4, 113.1, 111.2, 72.4, 70.5, 70.4, 69.2, 68.9, 63.4, 34.0, 19.1

Synthesis of PC5

Synthesis of 2,4,6 Tris(4-4'dimethoxydiphenylamino)-5-fluoroisophthalonitrile (PC5F)

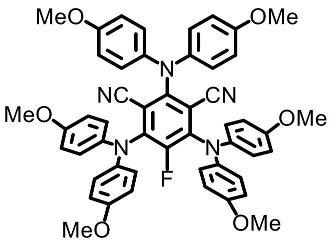

In an oven-dried Schlenk flask equipped with a magnetic stir bar, 4.2 equiv of 4-4'dimethoxydiphenylamine (21 mmol, 4814.7 mg) were dissolved in dry THF (0.3 M, 80 mL) under an atmosphere of argon. The reaction mixture was cooled at -78°C and then 4.2 equiv of LiHMDS (1 M in dry THF, 21 mmol, 21 mL) were added slowly. Finally, 1.0 equiv of tetrafluoroisophthalonitrile (0.1 M in dry THF, 1000.5 mg) was added and the resulting mixture was stirred at room temperature for 24h. The reaction mixture was quenched by the addition of water. After removal of THF, the residue was dissolved in DCM and the organic phase was extracted with DCM. Then, the organic phase was dried over Na_2SO_4 and recrystallized in CH_2Cl_2/pentane 1/2. 3020.0 mg of product was obtained (73% yield), orange solid.

^{1}H NMR (400 MHz, $CDCl_3$): δ (ppm) 6.94-6.88 (m, 12H), 6.81-6.78 (m, 12H), 3.78 (s, 18H). **^{13}C-NMR** (100 MHz, $CDCl_3$): δ (ppm) 156.4, 155.9, 152.4, 152.4, 152.1 (d, $^1J_{C,F}$=260 Hz), 149.6, 143.3, 143.1, 139.6, 139.3, 124.2, 124.1, 114.6, 114.6, 113.4, 113.4, 106.2, 106.1, 55.4, 55.4. **^{19}F-NMR decoupled ^{1}H** (376 MHz, $CDCl_3$): δ (ppm) -124.0 (s, 1F).

Synthesis of 2,4,6 Tris(4-4'dimethoxydiphenylamino)-5-triethylenglycol isophthalonitrile (PC5TEG)

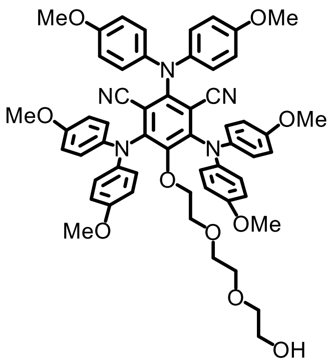

In an oven-dried Schlenk flask equipped with a magnetic stir bar, 1.0 equiv of PC5F (0.5 mmol, 413.9 mg) and 1.2 equiv of NaH 60% (24.0 mg) in oil were dissolved in dry DMAc (0.2 M, 2.5 mL) under an atmosphere of argon. Then, 3.5 equiv of triethylene glycol (1.75 mmol, 230 μL) were added to the solution. The resulting reaction mixture was heated at 100 °C and left at reflux overnight. The suspension was quenched by the addition of water, extracted with DCM and washed with LiCl (5% p/V). The organic phase was dried over Na_2SO_4, and the solvent was removed under reduced pressure. The crude product was purified by column chromatography on silica gel with DCM/EtOAc, 10-70% EtOAc as gradient. 344.8 mg of product obtained (70% yield), orange solid.

^{1}H NMR (400 MHz, $CDCl_3$): δ (ppm) 6.96-6.83 (m, 12H), 6.78-6.75 (m, 12H), 3.75 (s, 18H), 3.69-3.64 (m, 4H), 3.47 (t, *J*=4.0 Hz, 2H), 3.35 (t, *J*=4.0 Hz, 2H), 3.05 (t, *J*=4.0 Hz, 2H), 2.92 (m, 2H). **^{13}C-NMR** (100 MHz, $CDCl_3$): δ (ppm) 155.9, 152.1, 148.9, 148.5, 139.7, 139.5, 136.1, 125.8, 124.0, 123.5, 114.5, 113.7, 108.5, 71.9, 70.4, 69.1, 68.8, 64.3, 63.7, 55.4.

Synthesis of 2,4,6 Tris(4-4'dimethoxydiphenylamino)-5-tryethylenglicolisobutyrate isophthalonitrile (PC5)

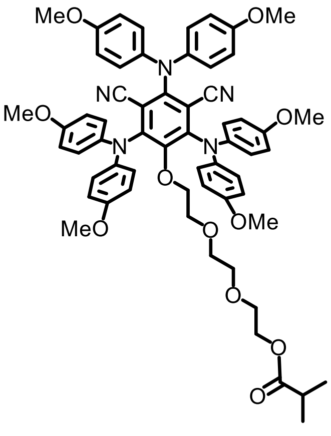

In an oven-dried Schlenk flask equipped with a magnetic stir bar, 1.0 equiv of PC5TEG (1.8 mmol, 1730.0 mg) and 1.5 equiv of isobutyryl chloride (2.7 mmol, 260 μL) were dissolved in dry THF (0.15 M, 12 mL) under an atmosphere of argon. Then, 1.6 equiv of triethylamine (2.9 mmol, 0.4 mL) were added slowly at the solution. The resulting reaction mixture was stirred overnight at room temperature. The suspension was quenched by the addition of water, washed with brine and extracted with DCM. The organic phase was dried over Na_2SO_4, and the solvent was removed under reduced pressure. The crude product was purified by column chromatography on silica gel with DCM/EtOAc, 10-20% EtOAc as gradient. 700.0 mg of product obtained (50% yield), red solid.

^{1}H NMR (400 MHz, $CDCl_3$): δ (ppm) 6.94-6.86 (m, 12H), 6.80-6.70 (m, 12H), 6.09 (s, 1H), 5.54 (s, 1H), 4.23 (t, *J* = 4.0 Hz, 2H), 3.75 (t, *J*=4.0 Hz, 18H), 3.66 (t, *J*=4.0 Hz, 2H), 3.60 (t, *J*=4.0 Hz, 2H), 3.35 (t, *J*=4.0 Hz, 2H), 3.10 (t, *J*=4.0 Hz, 2H), 2.91 (t, *J* = 4.0 Hz, 2H), 1.14 (d, *J* = 7.0 Hz, 3H). **^{13}C-NMR** (100 MHz, $CDCl_3$): δ (ppm) 178.3, 155.9, 152.1, 148.9, 148.5, 139.7, 139.5, 136.1, 125.8, 124.0, 123.5, 114.5, 113.7, 108.5, 71.9, 70.4, 69.1, 68.8, 64.3, 63.7, 55.4, 30.6, 18.3.

Synthesis of PC6

Syntehsis of 2,4,6 Tris(4-4'dibromodiphenylamino)-5-fluoroisophthalonitrile (PC6F)

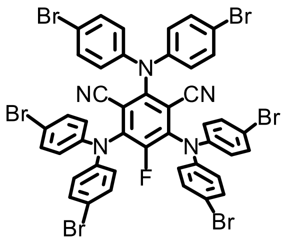

In an oven-dried Schlenk flask equipped with a magnetic stir bar, 4.2 equiv of 4-4'dibromodiphenylamine (21 mmol, 6867.0 mg) were dissolved in dry THF (0.3 M, 70 mL) under an atmosphere of argon. The reaction mixture was cooled at -78 °C and then 4.2 equiv of LiHMDS (21 mL, 1 M in dry THF) were added slowly. Finally, 1.0 equiv of tetrafluoroisophthalonitrile (5 mmol, 1000.5 mg, 0.1 M in dry THF) was added and the resulting mixture was stirred at room temperature for 24h at room temperature. The reaction mixture was quenched by the addition of water. After removal of THF, the residue was dissolved in DCM and the organic phase was extracted with DCM. Then, the organic phase was dried over Na_2SO_4 and recrystallized with CH_2Cl_2/pentane 1/2. 5000.0 mg of product obtained (90% yield), yellow solid.

^{1}H NMR (400 MHz, $CDCl_3$): δ (ppm) 7.41-7.39 (m, 12 H), 6.82-6.81 (m, 12 H). **^{13}C-NMR** (100 MHz, $CDCl_3$): δ (ppm) 144.0, 143.8, 142.4, 142.3, 132.9, 132.8, 124.3, 124.3, 118.5, 117.8, 112.2, 108.7. **^{19}F-NMR decoupled ^{1}H** (376 MHz, $CDCl_3$): δ (ppm) -120.2 (s, 1F).

Synthesis of 2,4,6 Tris(4-4'dibromodiphenylamino)-5-triethylenglycol isophthalonitrile (PC6TEG)

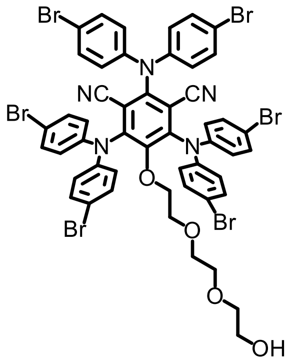

In an oven-dried Schlenk flask equipped with a magnetic stir bar, 1.0 equiv of PC6F (3 mmol, 3360 mg), with diphenylamine as donor moieties, and 1.2 equiv of NaH 60% in oil (144 mg) were dissolved in dry DMAc (0.2 M, 15 mL) under an atmosphere of argon. Then, 3.5 equiv of triethylene glycol (4.5 mmol, 1.4 mL) were added to the solution. The resulting reaction mixture was heated at 100 °C and left at reflux overnight. The suspension was quenched by the addition of water, extracted with DCM and washed with LiCl (5% p/V). The organic phase was dried over Na_2SO_4, and the solvent was removed under reduced pressure. The crude product was purified by column chromatography on silica gel with DCM/EtOAc, 10-50% EtOAc as gradient. 1400.0 mg of product was obtained (35% yield), yellow solid.

^{1}H NMR (400 MHz, $CDCl_3$): δ (ppm) 7.39-7.36 (m, 12H), 6.84-6.81 (m, 12H), 3.70 (dt, 4H), 3.52 (t, J=4.0 Hz, 2H), 3.41 (t, J=4.0 Hz, 2H), 3.17 (t, J=4.0 Hz, 2H), 3.02 (t, J=4.0 Hz, 2H). **^{13}C-NMR** (100 MHz, $CDCl_3$): δ (ppm) 150.6, 150.3, 147.4, 144.1, 144.0, 132.7, 132.7, 124.1, 123.8, 117.6, 117.4, 112.6, 110.9, 73.1, 72.4, 70.6, 70.2, 69.1, 61.7.

Synthesis of (2,4,6 Tris(4-4'dibromodiphenylamino)-5-tryethylenglicolisobutyrate isophthalonitrile (PC6)

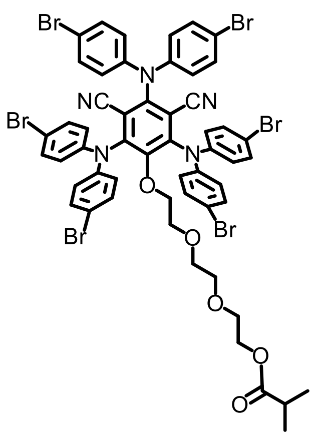

In an oven-dried Schlenk flask equipped with a magnetic stir bar, 1.0 equiv of PC6TEG (0.02 mmol, 25.0 mg) and 1.5 equiv of isobutyryl chloride (0.03 mmol, 2.5 μL) were dissolved in dry THF (0.075 M, 0.26 mL) under an atmosphere of argon. Then, 1.6 equiv of triethylamine (0.032 mmol, 3.5 μL) were added slowly to the solution. The resulting reaction mixture was stirred overnight at room temperature. The suspension was quenched by the addition of water, washed with brine and extracted with DCM. The organic phase was dried over Na_2SO_4, and the solvent was removed under reduced pressure. The crude product was purified by column chromatography on silica gel with DCM/EtOAc, 10-30% EtOAc as gradient. 13.2 mg of product obtained (50% yield), yellow solid.

^{1}H NMR (400 MHz, $CDCl_3$): δ (ppm) 7.38 (m, 12H), 6.81 (m, 12H), 4.18 (t, *J* = 5.0 Hz, 2H), 3.71 (t, *J*=4.0 Hz 2H), 3.59 (t, *J* = 4.0 Hz, 2H), 3.39 (t, *J*=4.0 Hz, 2H), 3.18 (t, *J*=4.0 Hz, 2H), 3.02 (t, *J*=4.0 Hz, 2H), 2.55 (hept, 1H), 1.15 (d, *J*=6.0 Hz, 6H). **^{13}C-NMR** (100 MHz, $CDCl_3$): δ (ppm) 178.3, 150.7, 150.3, 147.5, 144.1, 136.1, 132.7, 129.0, 128.5, 125.9, 124.1, 123.8, 117.6, 117.4, 112.6, 110.9, 73.1, 70.6, 70.3, 69.2, 63.6, 30.6, 18.3.

PC-monomers synthesis

General procedure

In an oven-dried Schlenk flask equipped with a magnetic stir bar, 1.0 equiv of the -OH terminated intermediate and 1.5 equiv of methacryloyl chloride were dissolved in dry THF (0.15 M, relating to the intermediate) under an atmosphere of argon. Then, 1.6 equiv of triethylamine (TEA) were added slowly to the solution. The resulting reaction mixture was stirred overnight at room temperature. The suspension was quenched by the addition of water, washed with brine and extracted with DCM. The organic phase was dried over Na_2SO_4, and the solvent was removed under reduced pressure. The crude product was purified by column chromatography on silica gel to obtain the product.

Synthesis of PC1MA

According to the general procedure, under inert atmosphere PC1TEG (1 eq, 0.3 mmol, 234 mg) was dissolved in 2 mL of dry THF. Then, triethylamine (1.2 eq, 0.36 mmol, 50 μL) was added dropwise and finally acryloyl chloride was also added (1.2 eq, 0.36 mmol, 30 μL). The reaction was stopped after 24h and extracted (adding DCM) with HCl 1M. After the extraction the solvent was evaporated and the crude purified by flash chromatography (gradient DCM, DCM:EtOAc 9:1 and DCM:EtOAc 8:2) giving an

isolated yield about 55%. The pure compound was characterized by ^{1}H-NMR, ^{13}C-NMR, lifetime measurements, absorption and emission spectra.

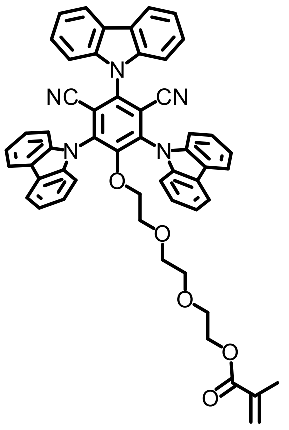

^{1}H NMR (400 MHz, $CDCl_3$): δ (ppm) 8.31-8.24 (m, 6H); 7.75 (d, J= 8.0 Hz, 2H); 7.70-7.61 (m, 10H), 7.50-7.42 (m, 6H), 5.96 (bs, 1H), 5.57 (bs, 1H), 4.11-4.03 (m, 6H), 3.32 (t, *J*= 4.0 Hz, 2H), 3.04 (t, *J*= 4.0 Hz, 2H), 2.82 (t, *J*=4.1 Hz, 2H), 2.47 (t, *J*= 4.0 Hz, 2H), 2.43 (t, *J*= 4.0 , 2H) 1.86 (bs, 3H). **^{13}C-NMR** (100 MHz, $CDCl_3$): δ (ppm) 167.1, 155.2, 140.3, 139.7, 138.9, 136.1, 126.8, 125.7, 124.4, 122.0, 121.2, 120.9, 116.4, 111.2, 110.2, 109.2, 74.1, 69.9, 69.6, 69.0, 68.7, 63.5, 18.3.

Synthesis of PC4MA

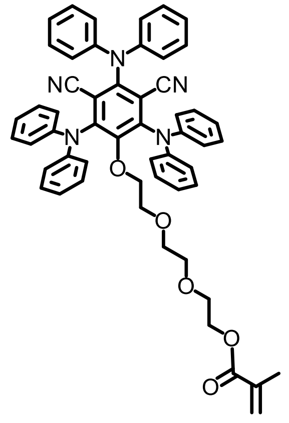

According to the general procedure using 207.0 mg of PC4TEG (0.25 mmol), 27 µL of methacryloyl chloride, 44 µL triethylamine (TEA) and 2.0 mL of dry THF. Column chromatography with DCM/EtOAc, 10-20% EtOAc as gradient. 139 mg of product obtained (58% yield), yellow solid.

^{1}H NMR (400 MHz, $CDCl_3$): δ (ppm) 7.28-7.26 (m, 12H), 7.08-7.00 (m, 18H), 6.12 (s, 1H), 5.57 (t, *J* = 4.8 Hz, 1H), 4.28-4.22 (m, 2H), 3.74 (t, *J* = 5.5 Hz, 2H), 3.61 (t, *J* = 4.8 Hz, 2H), 3.34 (t, *J* = 4.8 Hz, 2H), 3.04 (t, *J* = 4.8 Hz, 2H), 2.88 (t, *J* = 5.5 Hz, 2H), 1.95 (s, 3H) **^{13}C-NMR** (100 MHz, $CDCl_3$): δ (ppm) 166.6, 151.5, 150.9, 148.5, 145.7, 145.6, 136.3, 129.4, 129.4, 122.4, 113.1, 111.2, 72.5, 70.5, 70.4, 69.2, 68.9, 63.9, 31.1, 18.5.

Synthesis of PC6MA

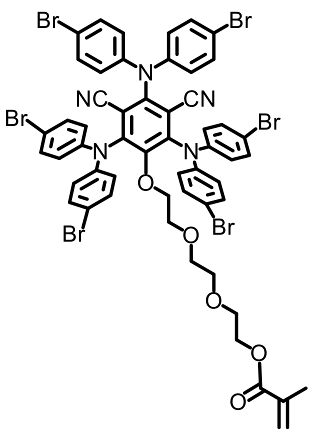

According to the general procedure, using 1350.0 mg of PC6TEG (1.0 mmol), 150 µL of methacryloyl chloride, 230 µL triethylamine (TEA) and 6.7 mL of dry THF. Column chromatography with DCM/EtOAc, 10-20% EtOAc as gradient. 659.5 mg of product obtained (50% yield), yellow solid.

^{1}H NMR (400 MHz, $CDCl_3$): δ (ppm) 7.49-7.35 (m, 12H), 6.84 (m, 12H), 6.12 (s, 1H), 5.58 (s, 1H), 4.28 (t, *J* = 4.8 Hz, 2H), 3.74 (t, *J*=4.0 Hz, 2H), 3.66 (t, *J*=4.0 Hz, 2H), 3.46-3.39 (m, 2H), 3.25-3.17 (m, 2H), 3.04 (t, *J*=4.0 Hz, 2H), 1.95 (s, 3H). **^{13}C-NMR** (100 MHz, $CDCl_3$): δ (ppm) 167.9, 150.7, 150.3, 147.5, 144.1, 136.1, 132.7,

129.0, 128.5, 125.9, 124.1, 123.8, 117.6, 117.4, 112.6, 110.9, 73.1, 70.6, 70.3, 69.2, 63.6, 18.4.

Synthesis of poly(PCMA-*co*-OEGMA)

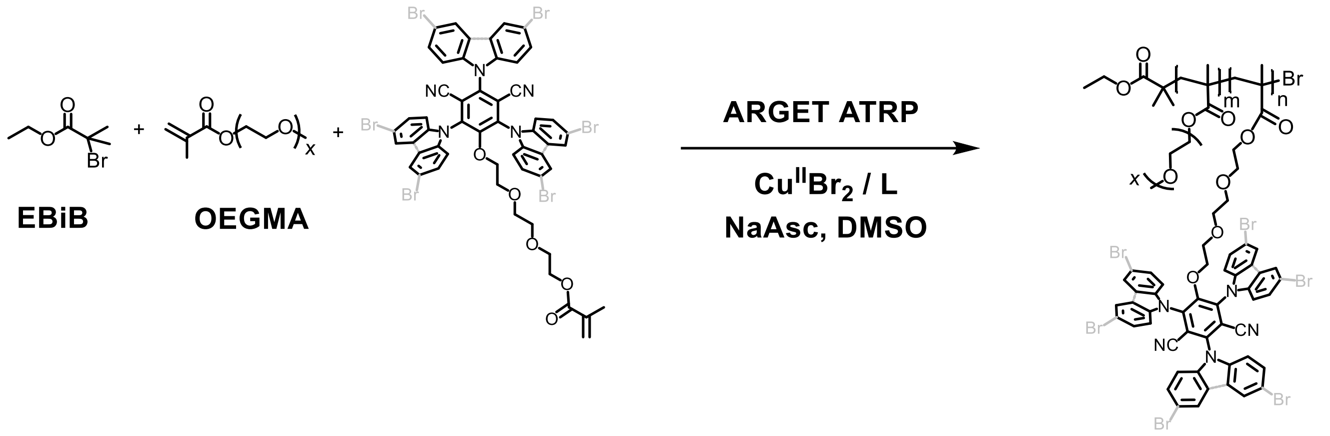

Scheme S2. General procedure for the copolymerization of OEGMA with the PC-bearing monomers through ARGET ATRP.

General procedure

Polymerizations were carried out in a 10 mL Schlenk flask at room temperature overnight (14h) using a total volume of solution of 5 mL, with DMSO as solvent, and keeping an atmosphere of argon. Targeting a 5 mol% incorporation of PC, 1.20 mL (2.70 mmol) of filtered monomer (OEGMA) was added to the solvent with a solvent/monomer ratio 1:3 (v/v). Then, 0.14 mmol of PC-MA were added to the solution. As catalyst, 1 mM $CuBr_2$ was used with TPMA as ligand ([$CuBr_2$]:[L], 1:3). The copper catalyst was added from a stock solution (0.02 M) prepared in DMSO and EBiB (0.014 mmol, 2 µL) was used as initiator with a target degree of polymerization (DP) of 200.

The Schlenk flask was sealed and the solution degassed with argon for 30 min. After that, 0.5 mM of NaAsc was added from a degassed aqueous stock solution, reaching a 2 vol % of water in the polymerization mixture. Finally the Schlenk flask was supplied with an argon balloon and the reaction was let at room temperature under stirring overnight.

Finally, the copolymers incorporating the photocatalysts were purified using 3.5 kDa dialysis membranes, for 2 days in 100% ACN and 1 day in 100% MilliQ water. Each copolymer was then let 1 day in the lyophilizer before use, yielding a bright yellow polymer. The final material was characterized with ^{1}H-NMR, GPC, absorption and emission spectra, and cyclic voltammetry. The actual incorporation of the catalyst was estimated by ^{1}H-NMR and UV-vis spectroscopy, using a calibration curve based on the absorption of a PCMA.

Table S1. Calculated incorporation of PC-MA in the statistical copolymers.

Copolymer	^{1}H-NMR incorporation[a]	UV-vis incorporation[b]
P(PC1MA-*co*-OEGMA)	4.5 mol%	4.2 mol%
P(PC4MA-*co*-OEGMA)	3.2 mol%	1.9 mol%
P(PC6MA-*co*-OEGMA)	4.8 mol%	4.0 mol%

[a] ^{1}H-NMR incorporations were estimated considering the ratio between the sum of the integrals of the peaks of aromatic protons and the broad singlet at 4.15 ppm corresponding to a diagnostic methylene (Figure S2, Figure S4 and Figure S6); [b]UV-Vis incorporation was calculated by building a calibration line of the corresponding PC (Figure S42, Figure S44 and Figure S45).

P(PC1MA-*co*-OEGMA)

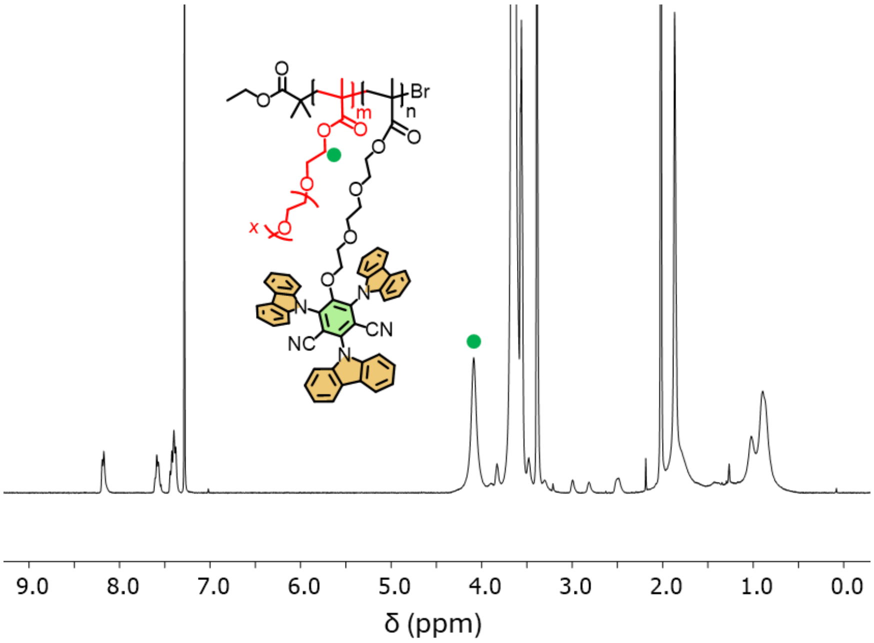

Figure S2. ^{1}H-NMR spectrum (400 MHz, $CDCl_3$) of P(PC1MA-*co*-OEGMA).

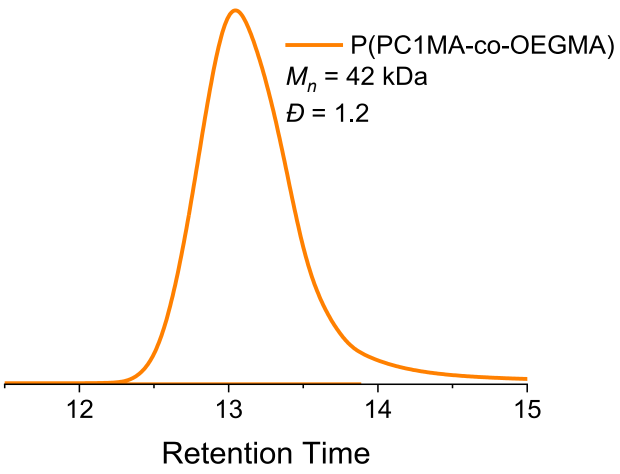

Figure S3. Size exclusion chromatography trace of poly(PC1MA-*co*-OEGMA).

P(PC4MA-*co*-OEGMA)

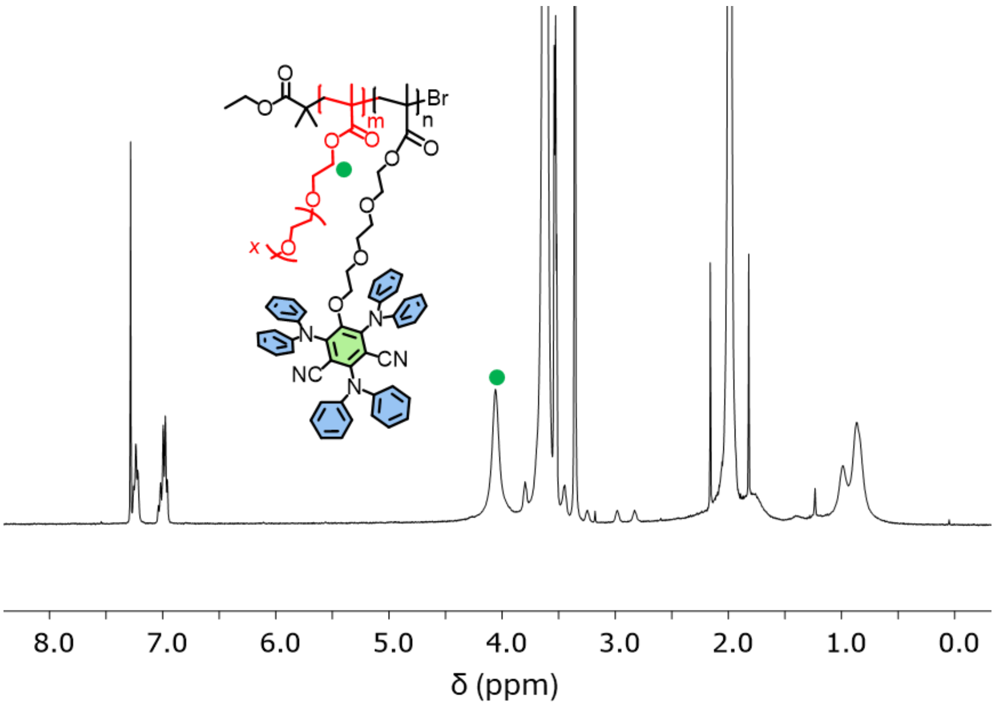

Figure S4. ^{1}H-NMR spectrum (400 MHz, $CDCl_3$) of P(PC4MA-*co*-OEGMA).

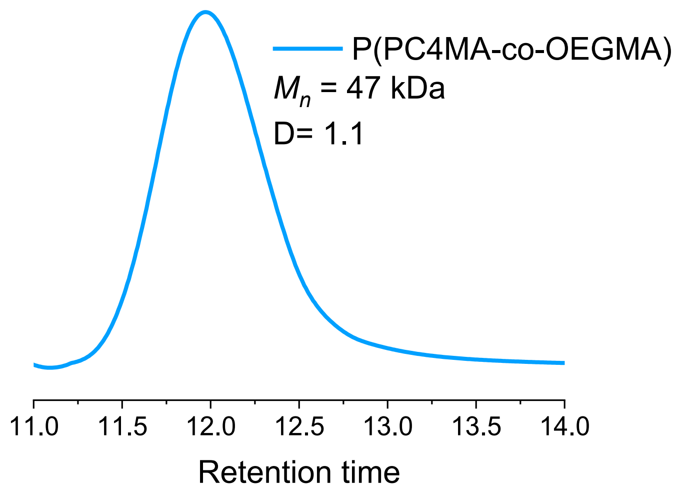

Figure S5. Size exclusion chromatography trace of P(PC4MA-*co*-OEGMA).

P(PC6MA-*co*-OEGMA)

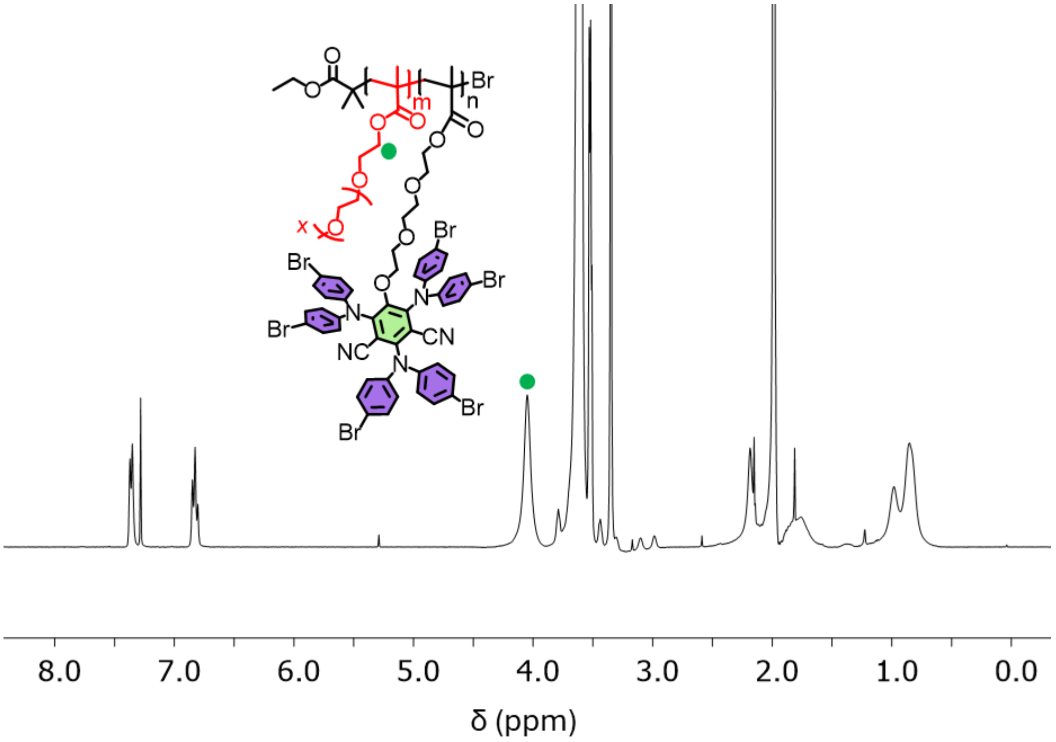

Figure S6. ^{1}H-NMR spectrum (400 MHz, $CDCl_3$) of P(PC6MA-*co*-OEGMA).

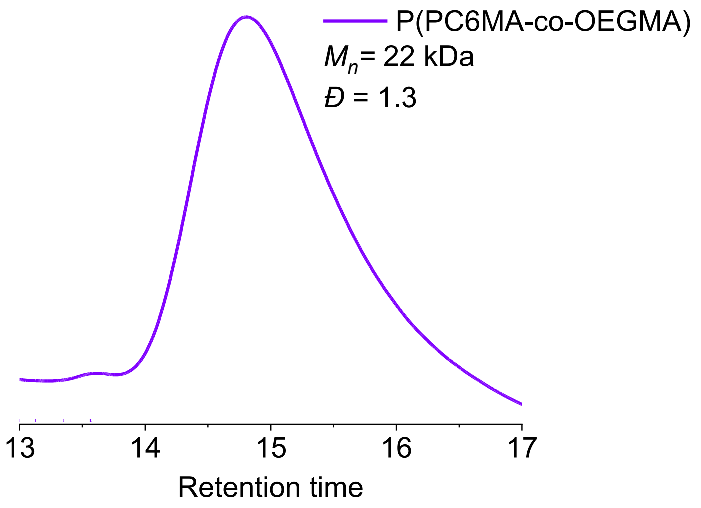

Figure S7. Size exclusion chromatography trace of P(PC6MA-*co*-OEGMA).

From SEC data and UV-Vis analysis, which allows us to estimate PC incorporation, it was possible to calculate the average content of PCMA moiety per copolymer chain:

- **P(PC1MA-*co*-OEGMA)** 42 kDa with 4.2 mol% of catalyst incorporation (from UV-Vis)
- **P(PC4MA-*co*-OEGMA)** 47 kDa with 1.9 mol% of catalyst incorporation (from UV-Vis)
- **P(PC6MA-*co*-OEGMA)** 22 kDa with 4.0 mol% of catalyst incorporation (from UV-Vis)

For P(PC1MA-*co*-OEGMA) we can consider 95.8% of $OEGMA_{500}$ and 4.2% of PC1MA (MW= 839.95 g/mol). It can be calculated that the average amount of PC1MA per chain is 3.4 units. Repeating the same calculation, we can estimate 1.7 catalytic units per chain for PC4MA and 1.7 for PC6MA.

Synthesis of SiO_2-P(PC1MA-*co*-OEGMA)

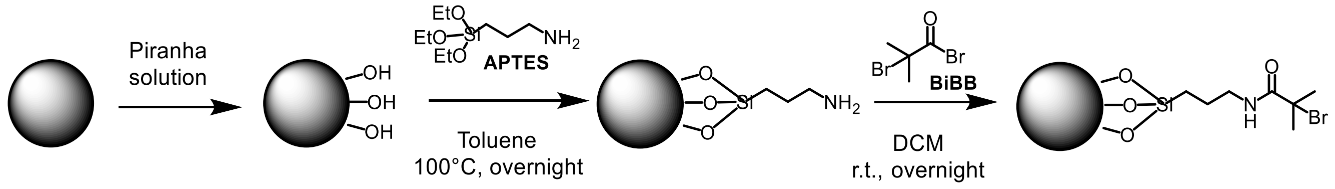

Scheme S3. Procedure for the immobilization of the ATRP initiator on silica particles.

200 nm particles were added in a beaker containing a piranha solution (H_2SO_4 and H_2O_2, 3:1 v/v). After 30 minutes the piranha solution was diluted with MilliQ water and immersed in an ice bath letting the particles to sediment. After 2h the diluted piranha solution was removed using a Pasteur pipette and added again with MilliQ water, repeating the same procedure until the solution was no more acidic (checked with a litmus paper). The particles

were then transferred in a 15 mL falcon and washed one more time with water and 2 times with EtOH (~10 mL). The three centrifuges were performed at 6000 rpm for 5 minutes. After this procedure the activated particles were let under vacuum overnight to dry.

The activated SiO_2 particles (100 mg) were suspended in a vial with dry toluene (5 mL) under magnetic stirring. Under stirring, APTES (100 µL, 427 mmol) was added. The sealed vial was put under vigorous stirring for 14h at 100 °C. The suspension was transferred into a 15 mL vial and toluene was added. The mixture was centrifuged for 5 minutes at 6000 rpm and the supernatant gently removed. This operation was repeated one more time with toluene and 3 times with EtOH. The particles where then let to dry overnight under vacuum.

The APTES-functionalized particles obtained from the previous step were put in a clean vial with 5 mL of dry DCM. The solution was degassed for 30 min and, under stirring, TEA (119 µL, 854 µmol) and then BBiB (106 µL, 854 µmol) were added. The reaction was stirred under inert atmosphere at room temperature for 14h. Following the same procedure, the suspension was transferred to a 15 mL falcon and washed 2 times with DCM and 3 times with EtOH (about 10 mL each wash) and then let to dry overnight.

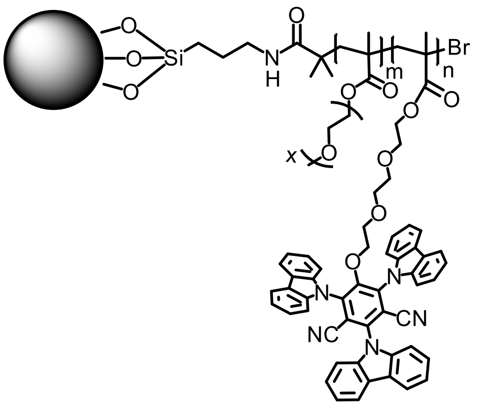

The polymerization was performed using 30 mg of initiator-bearing particles and following the same polymerization procedure reported above (but no EBiB in solution).

The brushes functionalized NPs were transferred in a 15 mL falcon and purified with several washes before use. Namely, 2 washes with DMSO, 2 with EtOH, 1 with MilliQ water and EDTA and 2 more with EtOH. Finally, they were dried under vacuum overnight. The amount of photocatalyst per mg of particles was estimated using UV-vis emission spectroscopy by means of a calibration curve.

Grafting density

The grafting density of polymer brushes grafted from NPs was estimated using a representative batch of NPs functionalized with POEGMA homopolymer brushes, obtained through SI-ARGET ATRP processes analogous to those employed for the synthesis of SiO_x-P(PC1MA-*co*-OEGMA).

For the calculation of the grafting density (σ) the following equation was used:

$$\sigma = \frac{\left(1 - f_{SiO_x}\right) \times N_{Av} \times \rho_{SiO_x} \times V}{A \times f_{SiO_x} \times M_n}$$

Where f_{SiOx} is the inorganic (SiO_x) fraction measured by TGA, N_{Av} is the Avogadro number, ρ_{SiOx} is the density of SiO_x nanoparticles (2.2 g/cm^3), V is the average volume of SiO_x nanoparticles (200 nm), A the superficial area and

M_n is the overall number-average MW of the cleaved polymer brushes. TGA characterization provided 65% of weight loss for the organic (polymeric) mass. Whereas SEC on "detached" brushes gave a M_n=170 kDa. From these data we could estimate a σ = 0.47 chain nm^{-2}.

Having estimated σ, and considering (i) the amount of PC per mg of NPs (2 x 10^{-5} mmol of PC1MA in 1 mg of functionalized NPs), and (ii) the average diameter of the NPs (200 nm) we could calculate an average of 0.9 PC units per nm^2.

Photoreactions

Povarov-type photoreaction

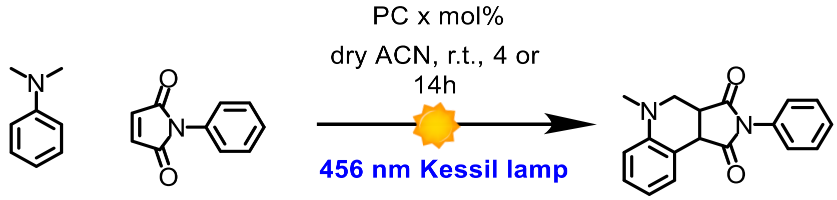

Scheme S4. Povarov-type photoreaction: [4+2] cycloaddition.

Following an existent procedure,[1] Povarov-type reactions were performed at 0.1 mmol scale with a variable loading of the catalyst. *N*-phenylmaleimide (17.3 mg, 1 eq, 0.1 mmol) was dissolved in 0.9 mL of dry ACN in a 4 mL vial. *N,N*-dimethylaniline (25 µL, 2 equivalents, 0.2 mmol) was then added at the solution dropwise under vigorous stirring at room temperature. The solution was irradiated with a 456 nm Kessil lamp at 50% intensity for 4h or 14h depending on catalyst loading (**Table 3**). The solvent was removed by pressure evaporation and the crude checked with ^{1}H-NMR to determine the ^{1}H-NMR yield using 1,3,5-trimethoxy benzene as internal standard.

When the photoactive copolymers were used for the recyclability test, after the evaporation of the ACN, Et_2O was added in order to precipitate the copolymer and recover the product of the reaction.

Reported mechanism of Povarov-type photoreaction

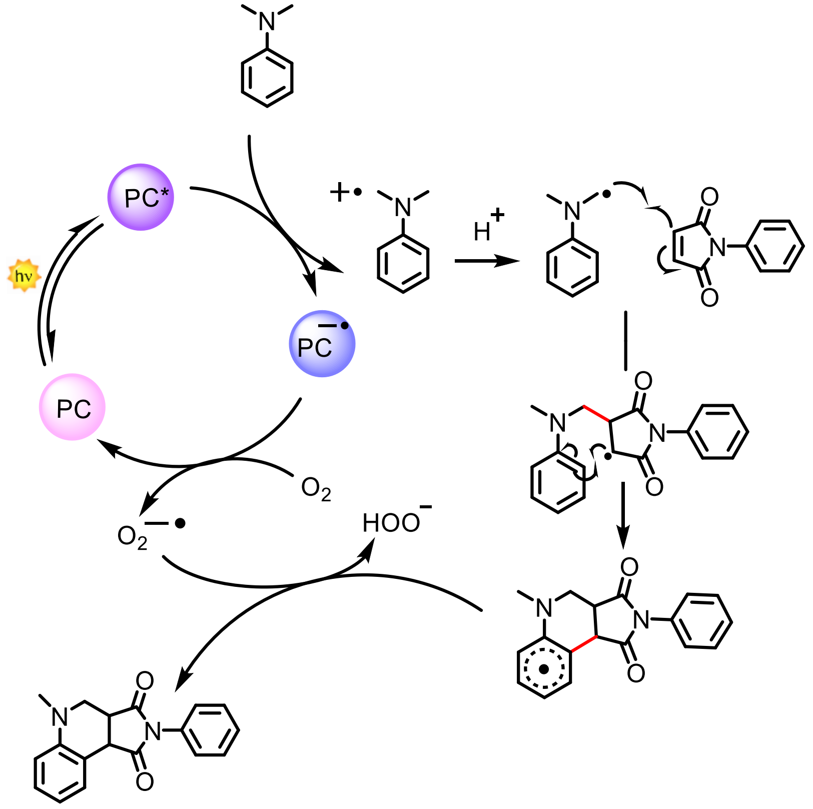

Scheme S5. Reported mechanism of the Povarov-type photoreaction, following a reductive quenching cylce. [2]

Photohydroxylation in water

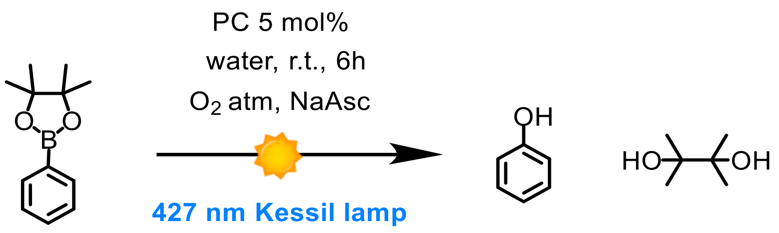

Scheme S6. Photohydroxylation of the pinacol ester of boronic acid in water.

Phenylboronic acid pinacol ester (11.4 mg, 1 eq, 0.056 mmol) and sodium ascorbate (39.6 mg, 4 eq, 0.24 mmol) were added to 4 ml of DI water. The reaction mixture was supplied with oxygen by balloons before being subjected to irradiation with a 427 nm Kessil lamp at 25% intensity for 6h. The solution was extracted with Et_2O (20 ml x 3), the organic solution was dried with Na_2SO_4 and the solvent removed by pressure evaporation. The crude was checked with ^{1}H-NMR to determine the ^{1}H-NMR yield using 1,3,5-trimethoxy benzene as internal standard.

Reported mechanism of the photohydroxylation

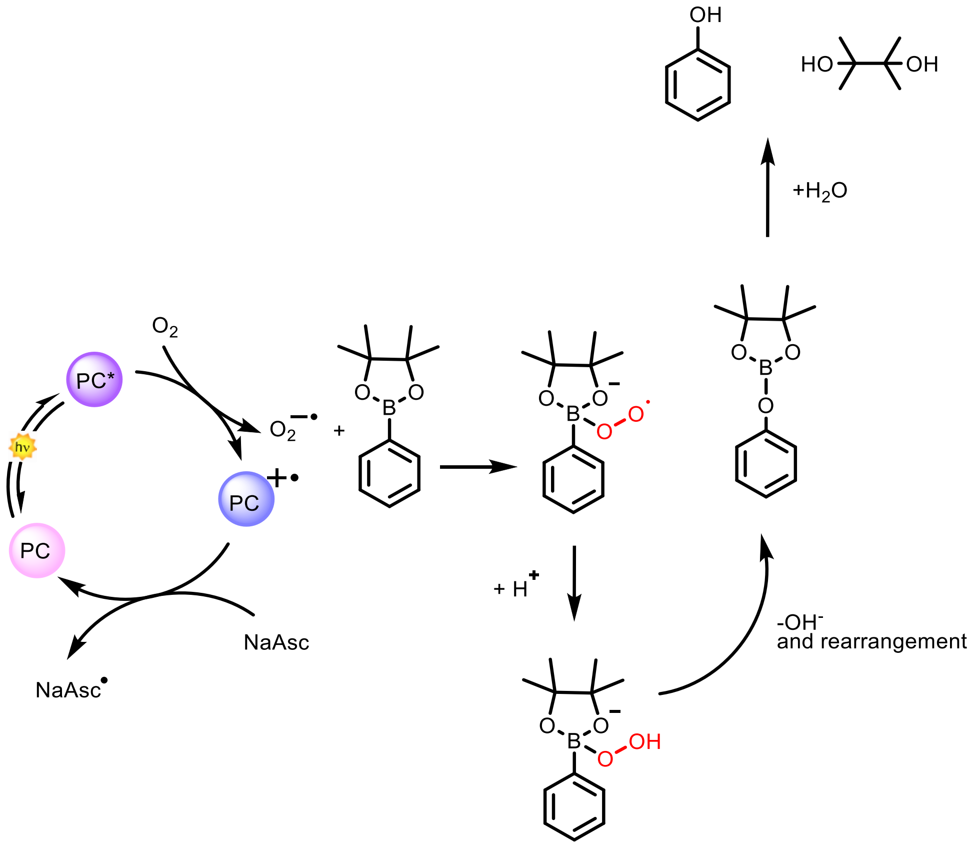

Scheme S7. Reported mechanism of the photohydroxylation of pinacol ester of boronic acid, following an oxidative quenching path.

Photocatalyzed [2+2] cycloaddition in water

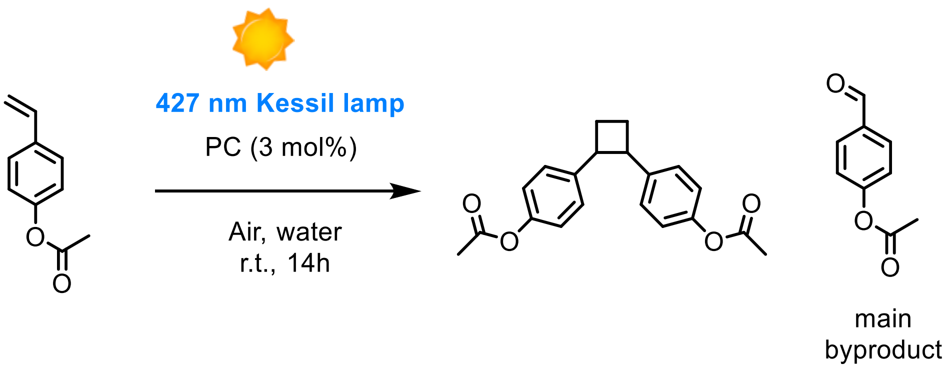

Scheme S8. Photocatalyzed [2+2] cycloaddition of 4-acetoxystyrene in water.

In a 8 mL vial, the water soluble P(PCMA-*co*-OEGMA) was dissolved in 2 mL of DI water corresponding to 3 mol% loading of the photocatalytic moiety obtaining a bright yellow solution. Under vigorous stirring 4-

acetoxystyrene (0.12 mmol, 16 μL) was added generating a turbid solution. The vial was let open to air at room temperature and put under blue light irradiation (427 nm Kessil lamp, 50% intensity) for 14h. At the end of the 14h water was removed by pressure evaporation and added with 1 mL of ACN to dissolve all the components. The solution was then transferred in a 15 mL falcon were the polymer was selectively precipitated by the addition of 12 mL of Et_2O. The falcon was centrifuged for 10 minutes at 5000 rpm and the surnatant transferred in a 25 mL round bottom flask to remove the solvent. The crude was checked with ^{1}H-NMR to determine the ^{1}H-NMR yield using 1,3,5-trimethoxy benzene as internal standard.

Photophysical studies

Absorption and emission measurement

Absorption spectra were recorded in a 0.1 mM solution of the catalyst in ACN. Emission spectra were recorded 0.01 mM solution of the catalyst in ACN. PC1, PC3, PC4 and PC6 were excited at 400 nm while PC2 and PC5 were excited at 425 nm.

Redox potentials measurement

Redox potentials were determined by the calculation of the half-wave potentials $E_{1/2}(PC/PC^{\bullet-})$, and the anodic peak potentials $E_{p,a}(PC^{\bullet+}/PC)$ in a solution 0.1 mM of PC in ACN using tetrabutylammonium hexafluorophosphate ($TBAPF_6$) 0.1 M as supporting electrolyte and a scan rate of 1 V/s.

Finally, since a quasi-reference electrode was used, ferrocene (Fc) was added at the end of each CV experiment as an internal standard, to refer all potentials to the saturated calomel electrode (SCE), considering that $E°(Fc^+/Fc)$ = 0.391 V *vs* SCE in ACN.

For the estimation of the potentials of the excited state, the Rehm-Weller formalism was applied: [3]

$$E(\mathrm{PC}^*/\mathrm{PC}^{\bullet-}) = E(\mathrm{PC}/\mathrm{PC}^{\bullet-}) + E_{0,0}$$

$$E(\mathrm{PC}^{\bullet+}/\mathrm{PC}^*) = E(\mathrm{PC}^{\bullet+}/\mathrm{PC}) - E_{0,0}$$

where:

- $E(\mathrm{PC}^*/\mathrm{PC}^{\bullet-})$ and $E(\mathrm{PC}^{\bullet+}/\mathrm{PC}^*)$ are the reduction potential of the photocatalyst in the exited state
- $E(\mathrm{PC}/\mathrm{PC}^{\bullet-})$ and $E(\mathrm{PC}^{\bullet+}/\mathrm{PC})$ are the reduction potential of the photocatalyst in the ground state
- $E_{0,0}$ is the energy of the singlet

Where $E_{0,0}$ was estimated from the intersection point between absorption and emission spectra converted in eV.

PC1 (2,4,6, Tris(3,6-carbazol-9-yl) 5-triethylenglycol isophthalonitrile)

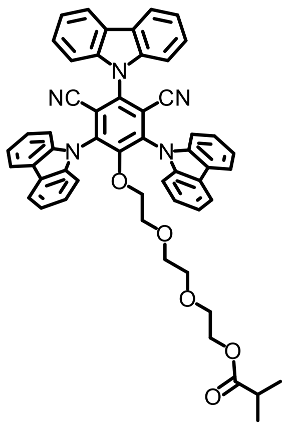

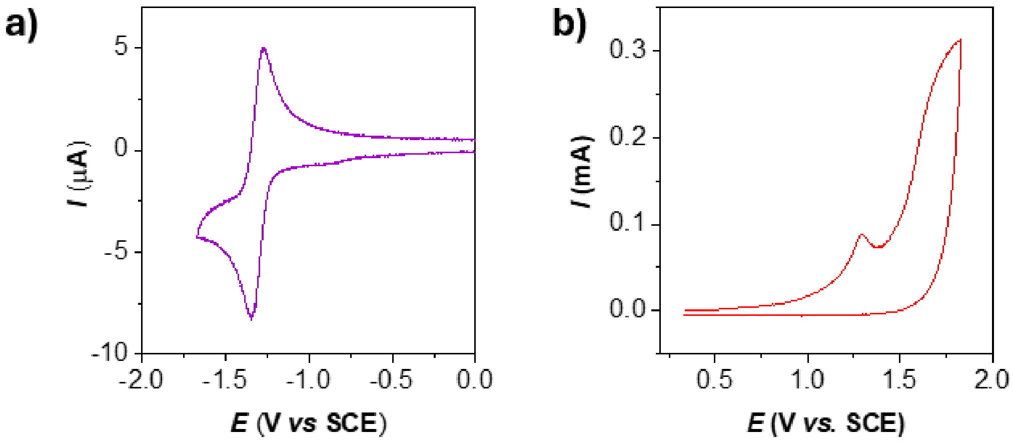

Figure S8. CV in the reduction a), and in the oxidation b) direction of a solution of 0.1 mM of PC1 in ACN using TEATFB 0.1 M as supporting electrolyte and a scan rate of 1 V/s.

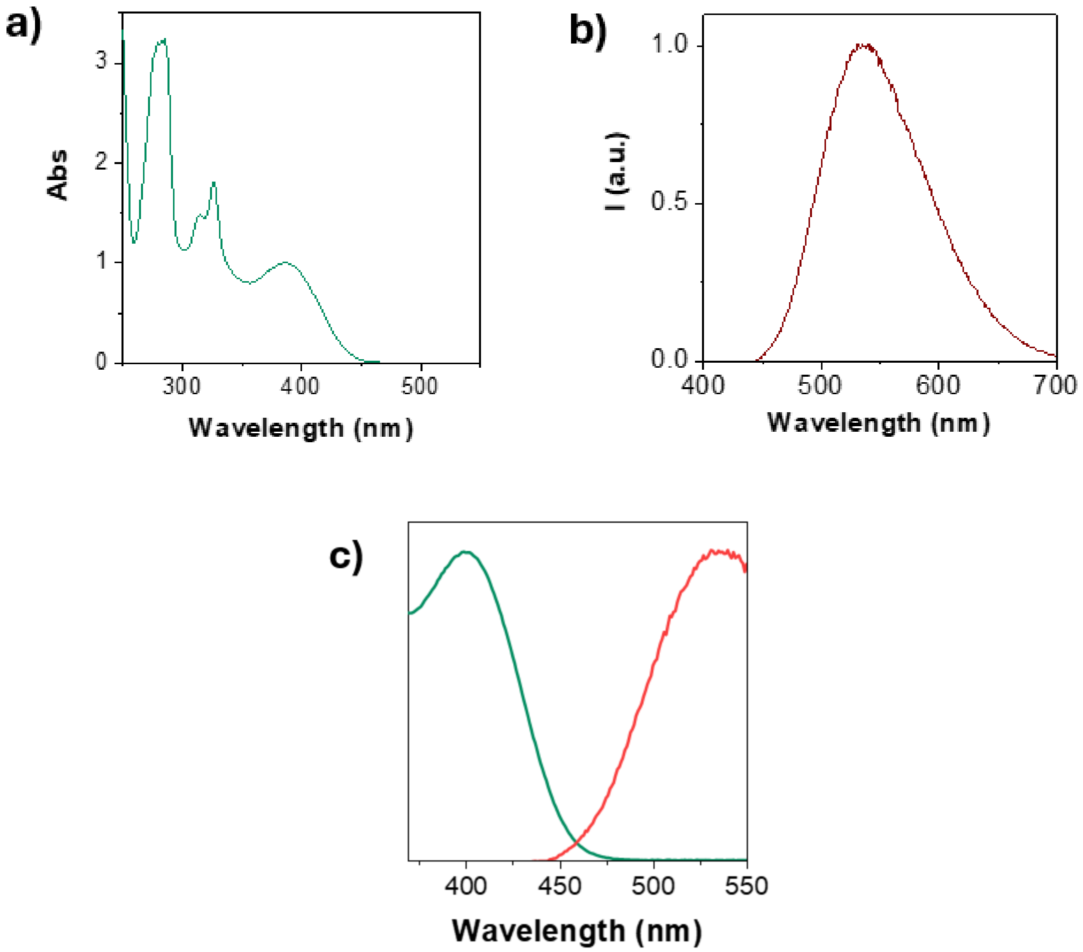

Figure S9. a) absorption and b) emission spectrum of PC1; c) intersection point of the spectra resulting in the crossing point at 458 nm. Converting results in $E_{0,0}$= 2.70 V.

PC2 (2,4,6 Tris(3,6-dimethoxy-carbazol-9-yl)-5-tryethylenglicolisobutyrate isophthalonitrile)

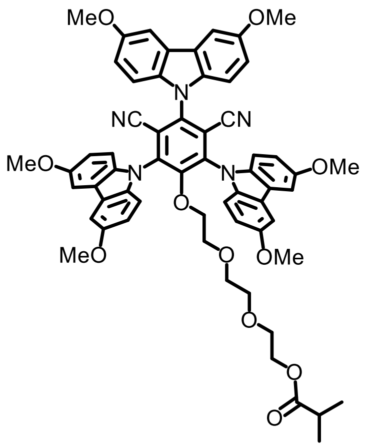

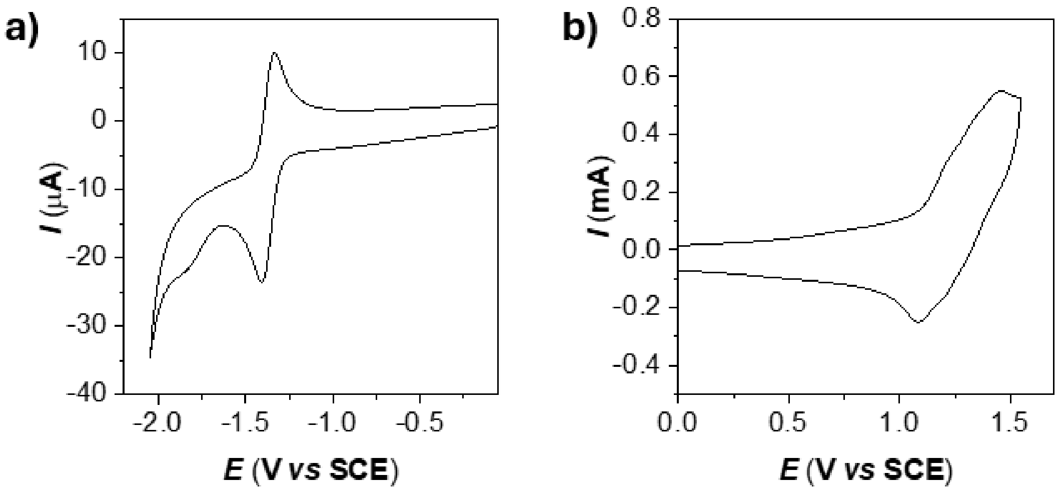

Figure S10. CV in the reduction a), and in the oxidation b) direction of a solution of 0.1 mM of PC2 in ACN using TEATFB 0.1 M as supporting electrolyte and a scan rate of 1 V/s.

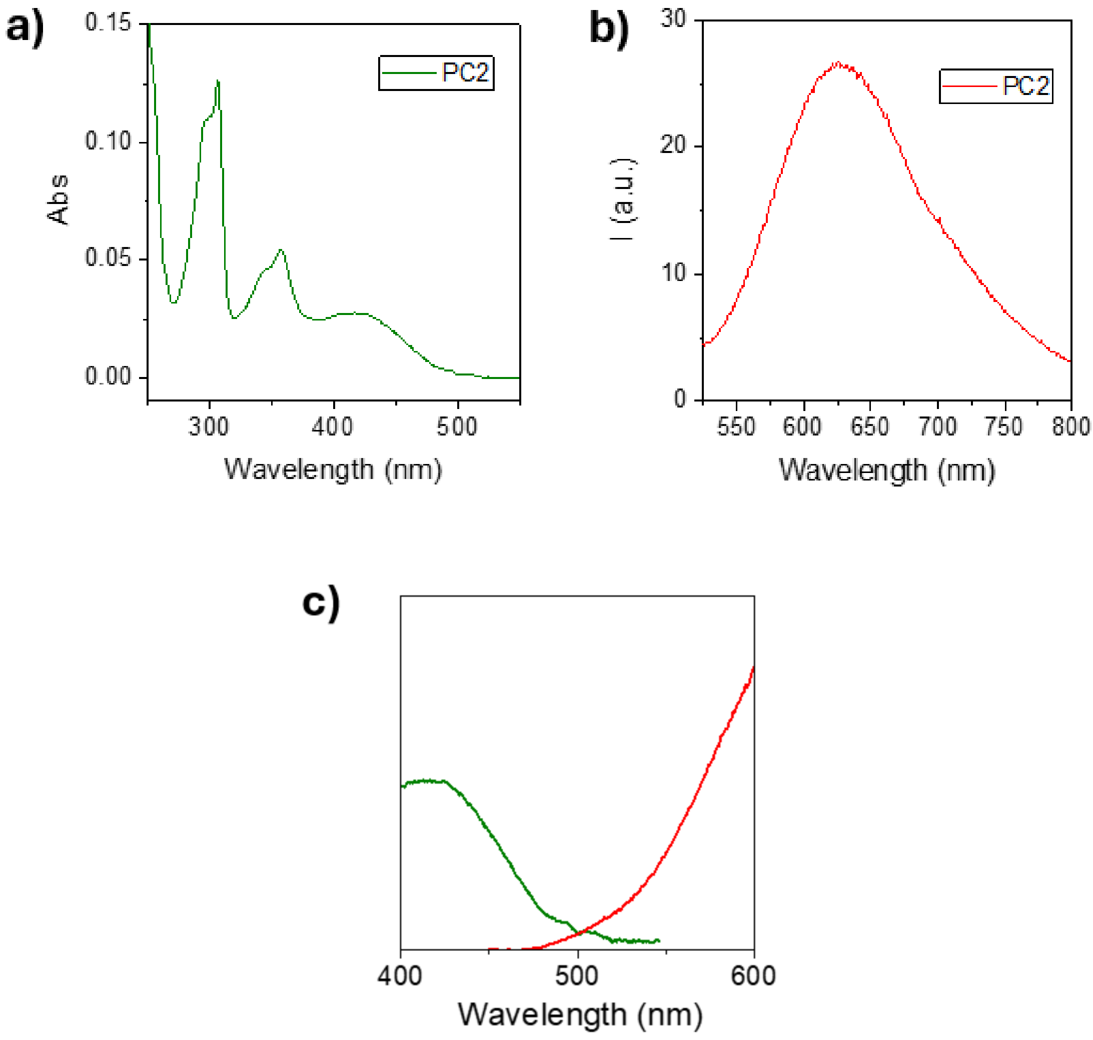

Figure S11. a) absorption and b) emission spectrum of PC2. Intersection point of the spectra resulting in the crossing point at 502 nm. Converting results in $E_{0,0}$= 2.47 eV

PC3 (2,4,6, Tris(3,6-dibromo-carbazol-9-yl) 5-triethylenglycol isophthalonitrile)

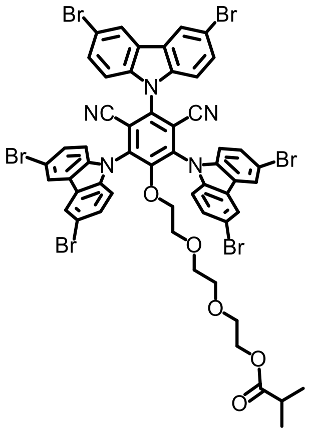

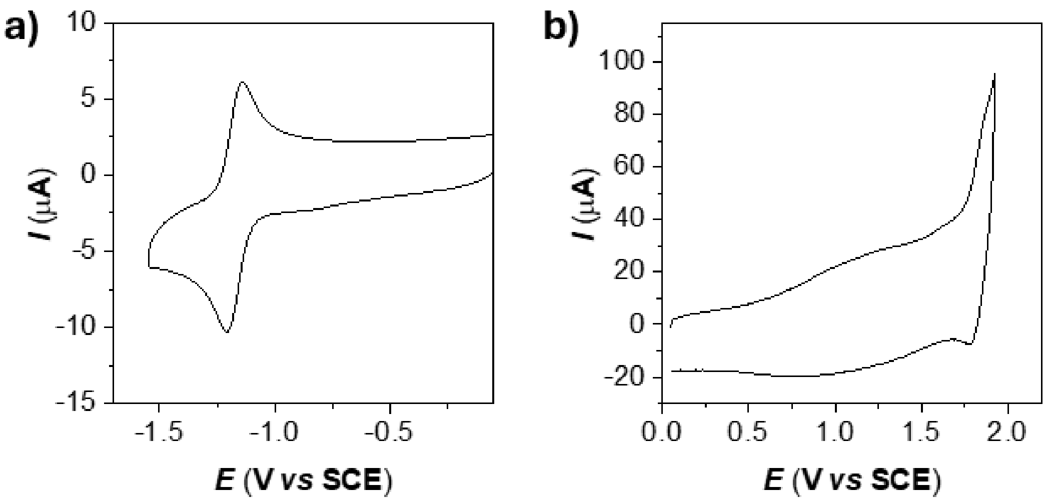

Figure S12. CV in the reduction a), and in the oxidation b) direction of a solution of 0.1 mM of PC3 in ACN using TEATFB 0.1 M as supporting electrolyte and a scan rate of 1 V/s.

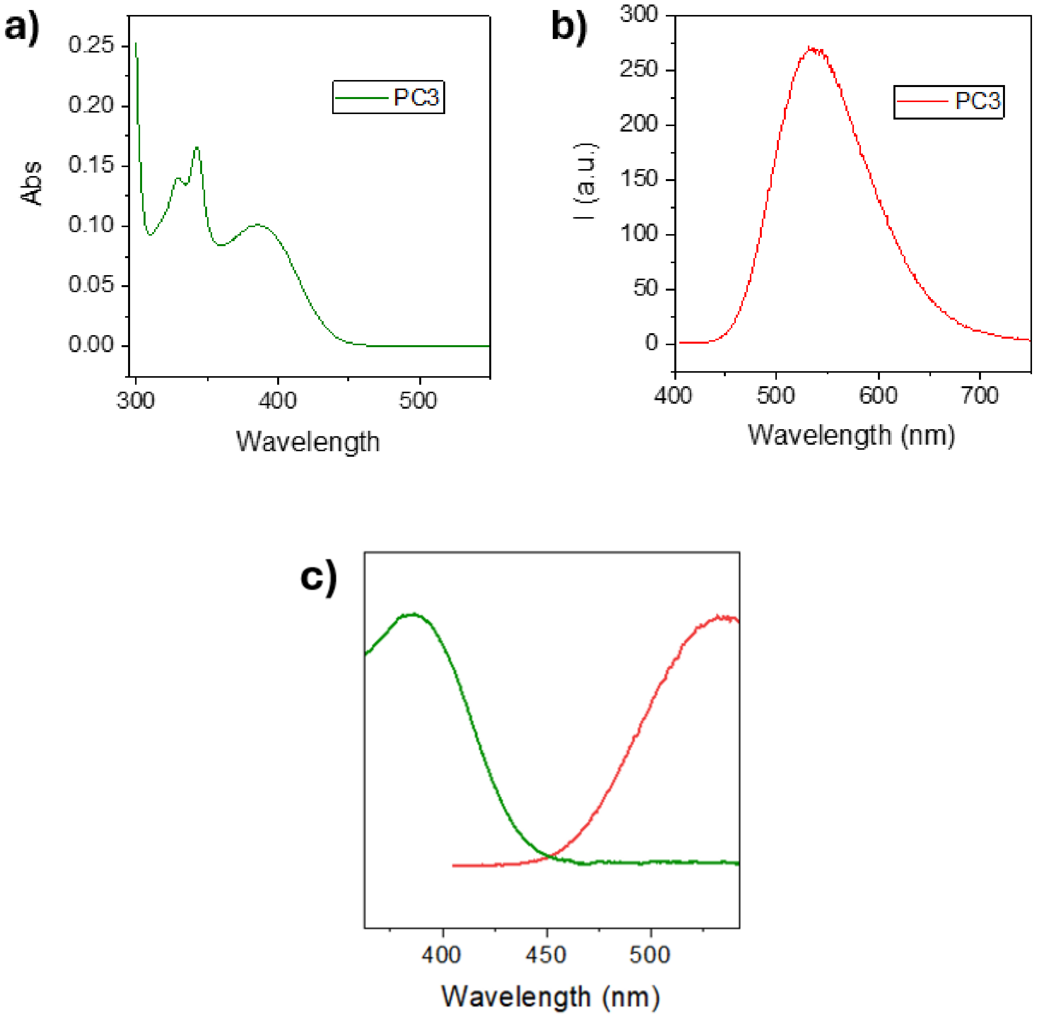

Figure S13. a) absorption and b) emission spectrum of PC3. c) Intersection point of the spectra resulting in the crossing point at 450 nm. Converting results in $E_{0,0}$= 2.75 V.

PC4 (2,4,6 Tris(diphenylamino)-5-tryethylenglicolisobutyrate isophthalonitrile)

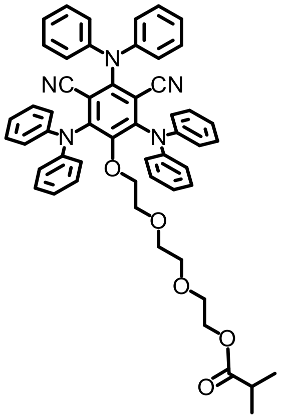

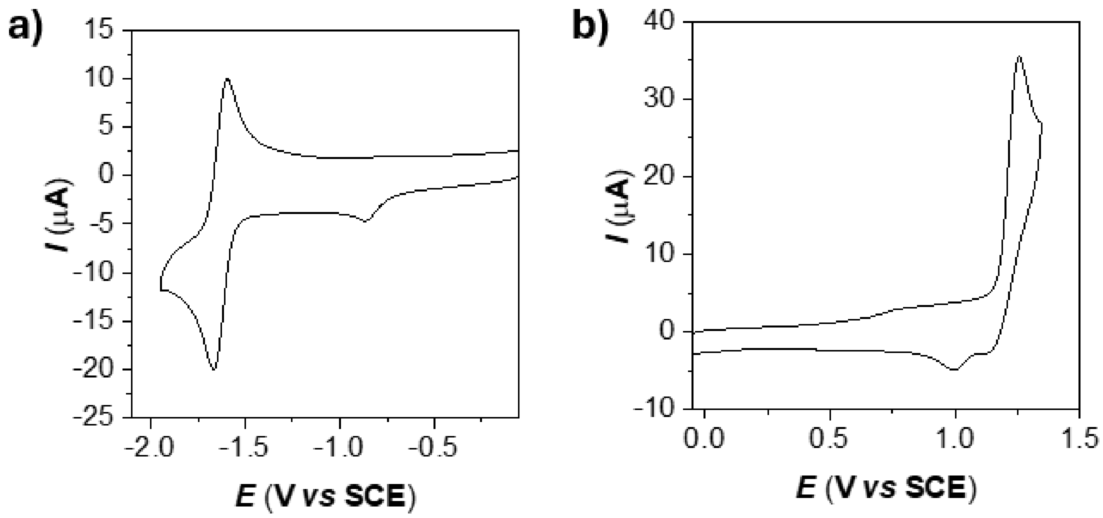

Figure S14. CV in the reduction a), and in the oxidation b) direction of a solution of 0.1 mM of PC4 in ACN using TEATFB 0.1 M as supporting electrolyte and a scan rate of 1 V/s.

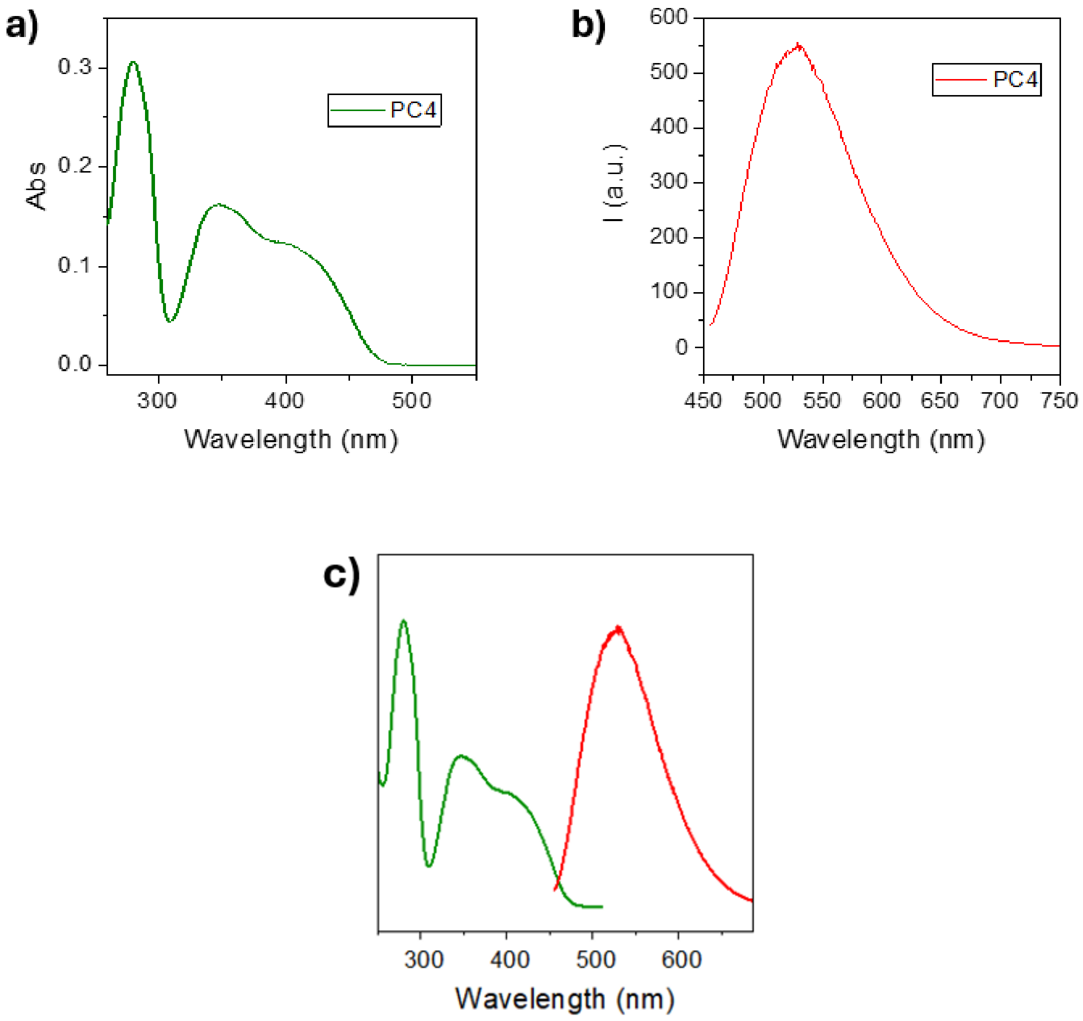

Figure S15. a) Absorption and b) emission spectrum of PC4, c) intersection point of the spectra resulting in the crossing point at 455 nm. Converting results in $E_{0,0}$= 2.72 V.

PC5 (2,4,6 Tris(4-4'dimethoxydiphenylamino)-5-tryethylenglicolisobutyrate isophthalonitrile)

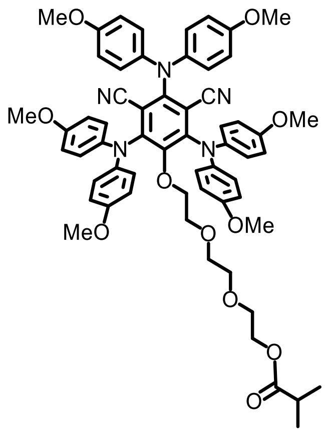

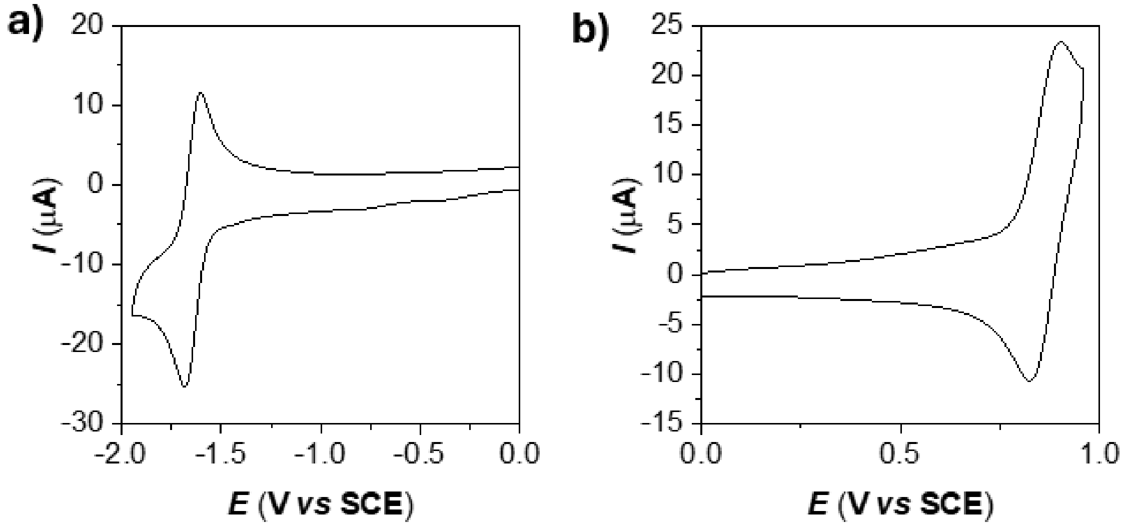

Figure S16. CV in the reduction a), and in the oxidation b) direction of a solution of 0.1 mM of PC5 in ACN using TEATFB 0.1 M as supporting electrolyte and a scan rate of 1 V/s.

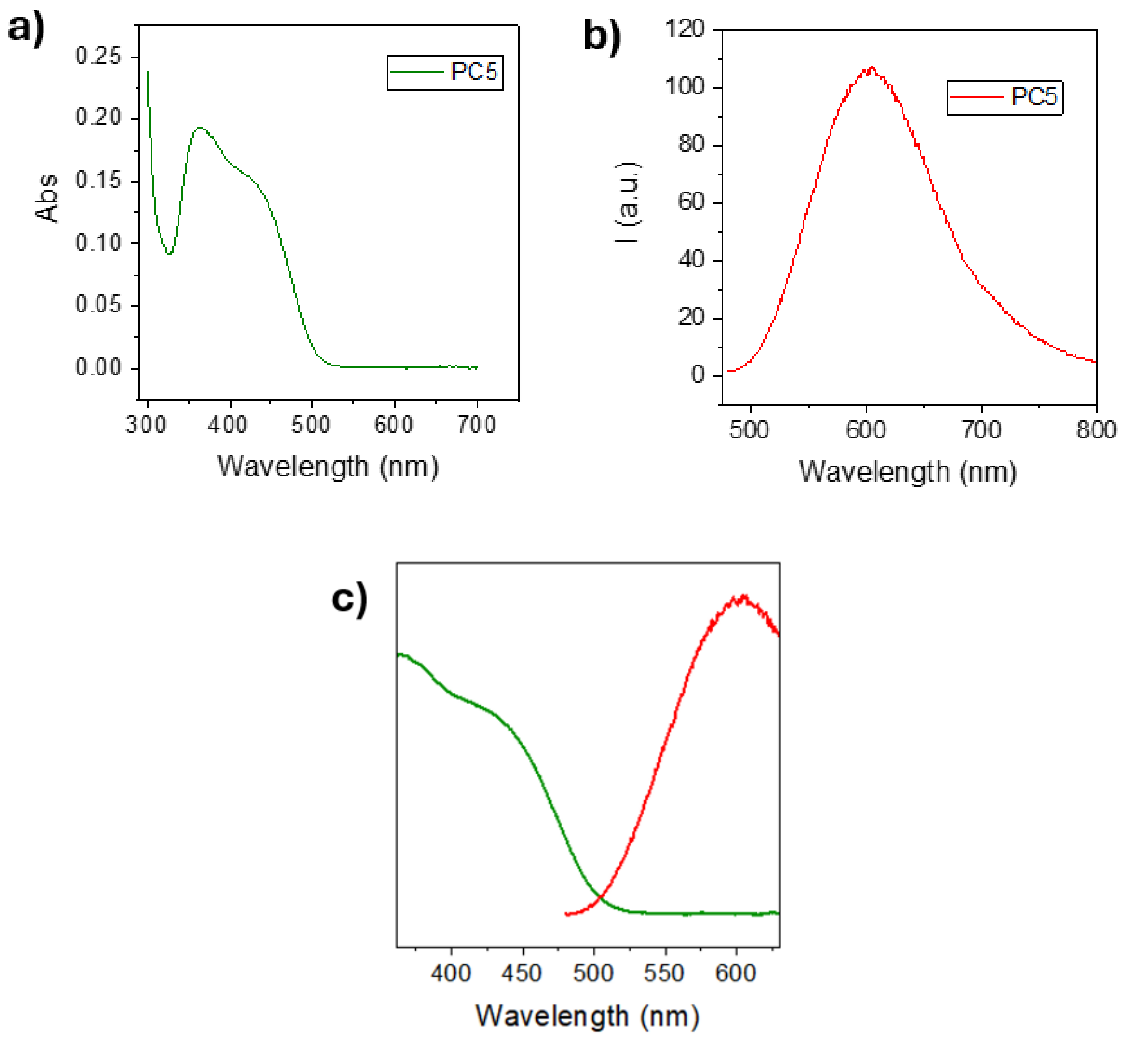

Figure S17. a) Absorption and b) emission spectrum of PC5, c) intersection point of the spectra resulting in the crossing point at 505 nm. Converting results in $E_{0,0}$= 2.45 V.

PC6 (2,4,6 Tris(4-4'dibromodiphenylamino)-5-tryethylenglicolisobutyrate isophthalonitrile)

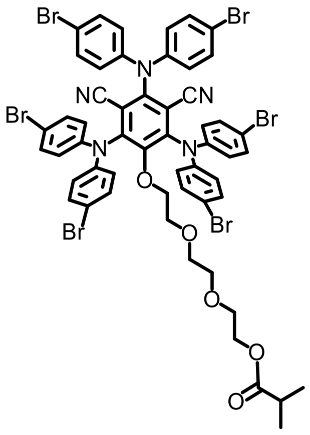

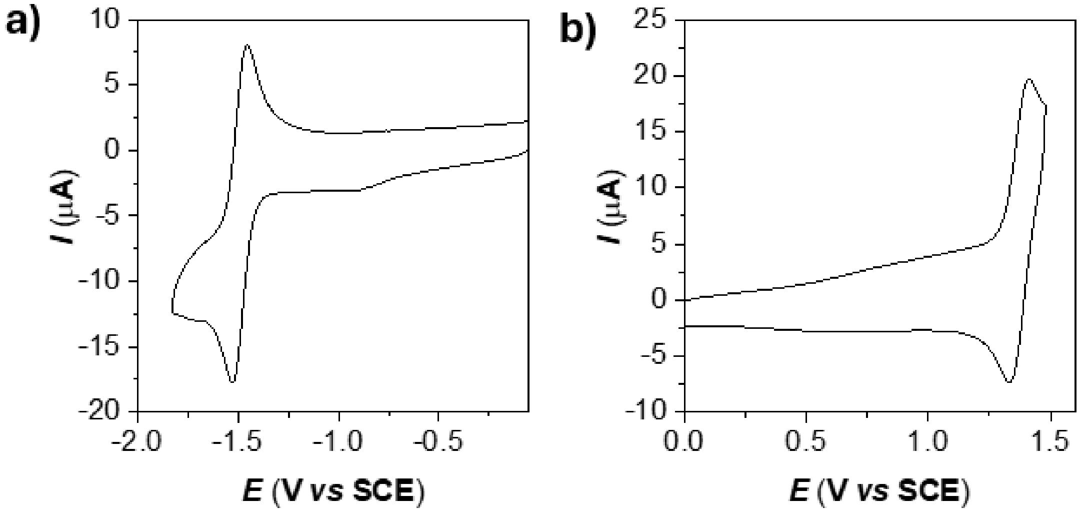

Figure S18. CV in the reduction a), and in the oxidation b) direction of a solution of 0.1 mM of PC6 in ACN using TEATFB 0.1 M as supporting electrolyte and a scan rate of 1 V/s.

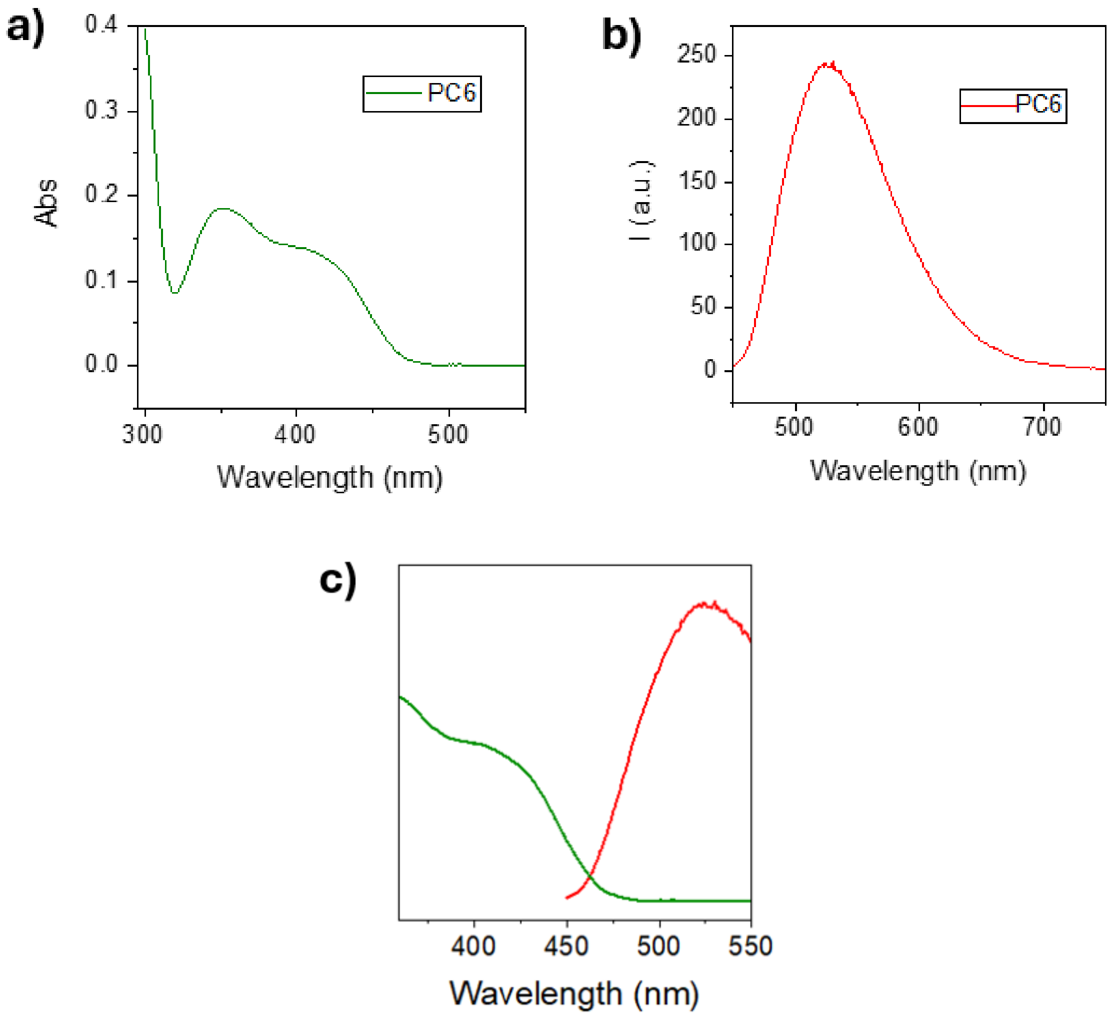

Figure S19. a) absorption and b) emission spectrum of PC6, c) intersection point of the spectra resulting in the crossing point at 464 nm. Converting results in $E_{0,0}$= 2.67 V.

Polymers redox potential summary

Table S2. Summary of redox properties of all PC-bearing copolymers. $E_{0,0}$ was calculated from emission and absorption spectra intersection, ground state potentials through cyclic voltammetry, and excited state potentials by exploiting the Rhem Weller formalism. Potentials are reported in V vs SCE.

PC	λ_{int} (nm)	$E_{0,0}$ (eV)	$E_{1/2}$(PC/PC$^{\bullet -}$) (V)	$E_{p,a}$(PC$^{\bullet +}$/PC) (V)	$E_{1/2}$(PC*/PC$^{\bullet -}$) (V)	$E_{p,a}$(PC$^{\bullet +}$/PC*) (V)
**P(PC1MA-*co*-OEGMA)**	445	2.74	-1.30	+1.21	+1.44	-1.53
**P(PC4MA-*co*-OEGMA)**	450	2.75	-1.6	+1.46	+1.15	-1.29

**P(PC6MA-*co*-OEGMA)**	463	2.67	-1.43	+1.42	+1.24	-1.25

P(PC1MA-*co*-OEGMA)

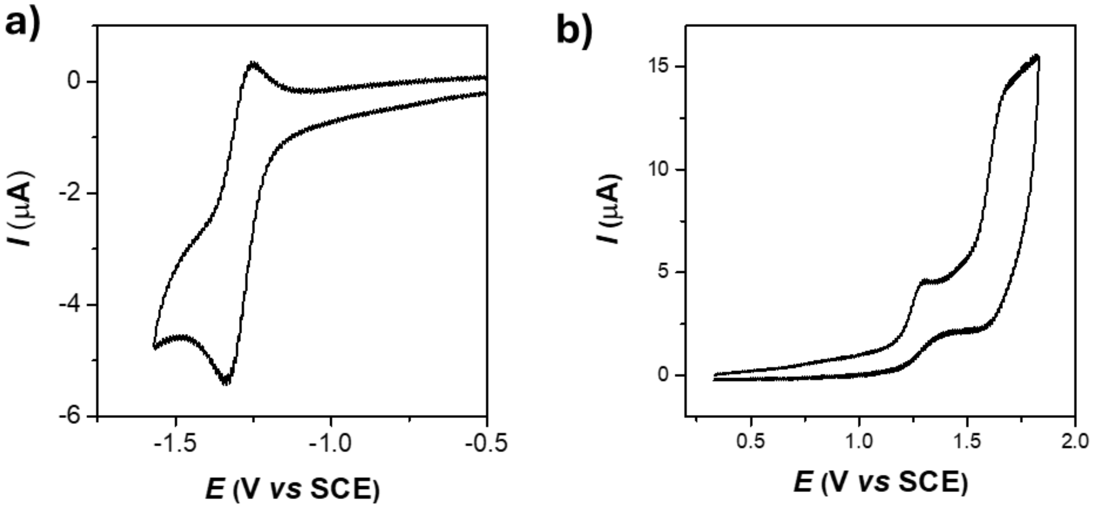

Figure S20. CV in the reduction a)and in the oxidation b) direction of a solution of 0.5 mM of poly(PC1-MA-*co*-OEGMA) in ACN using TEATFB 0.1 M as supporting electrolyte and a scan rate of 1 V/s. The concentration of the solution was calculated with respect to the estimated incorporation of photocatalyst.

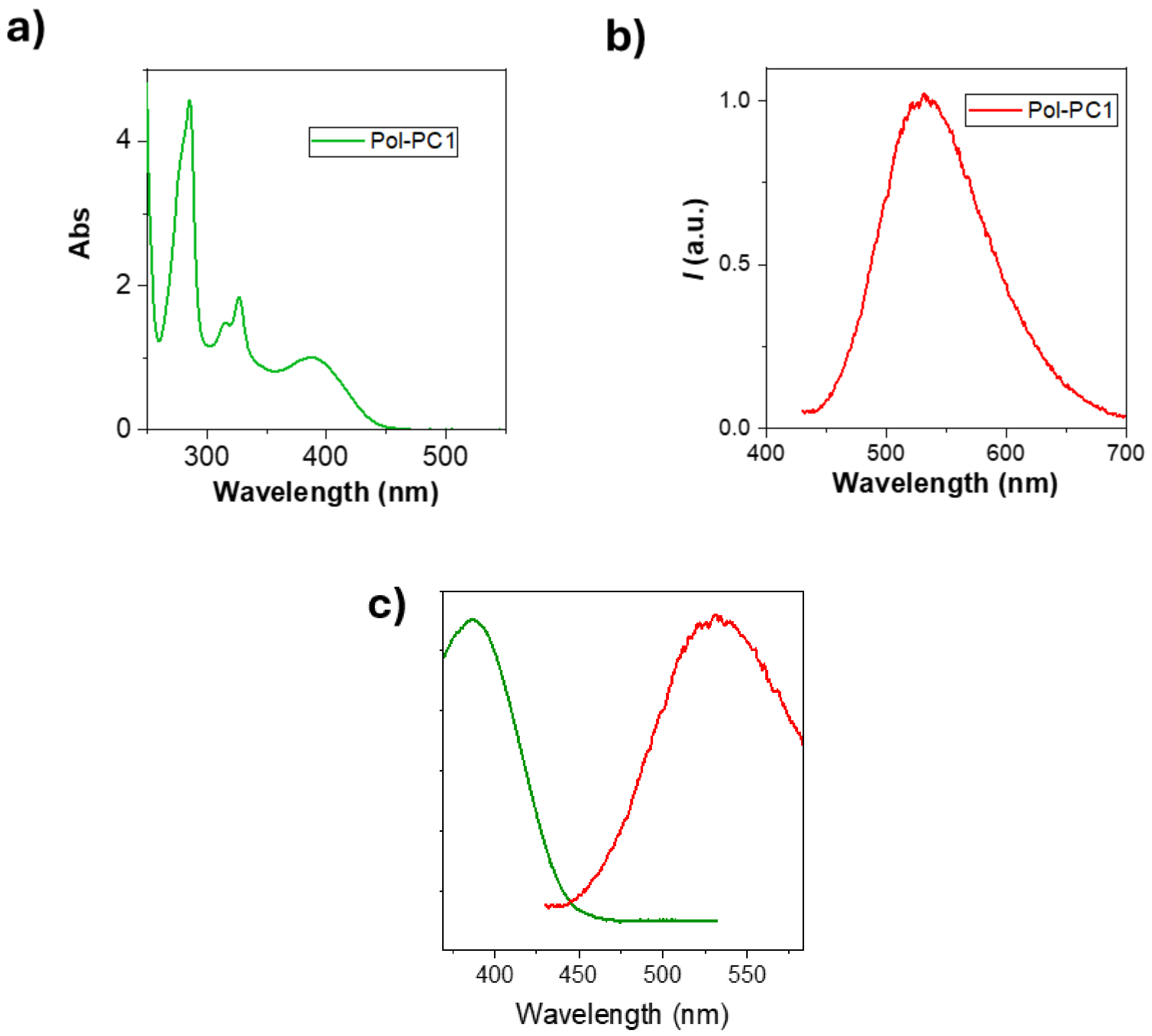

Figure S21. a) absorption and b) emission spectrum of P(PC1MA-*co*-OEGMA), c) intersection point of the spectra resulting in the crossing point at 445 nm. Converting results in $E_{0,0}$= 2.78 V.

P(PC4MA-*co*-OEGMA)

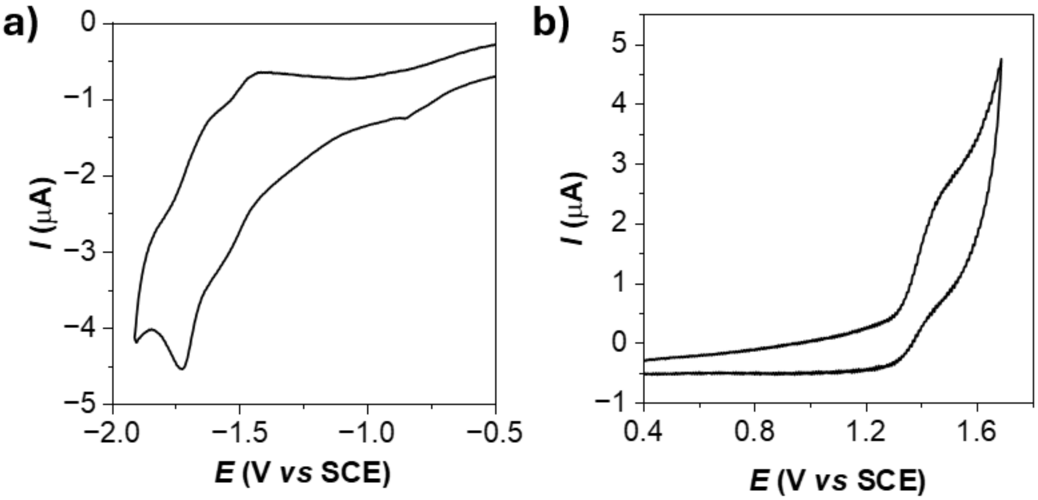

Figure S22. CV in the reduction a)and in the oxidation b) direction of a solution of 0.5 mM of P(PC4MA-*co*-OEGMA) in ACN using TEATFB 0.1 M as supporting electrolyte and a scan rate of 1 V/s. The concentration of the solution was calculated with respect to the estimated incorporation of photocatalyst.

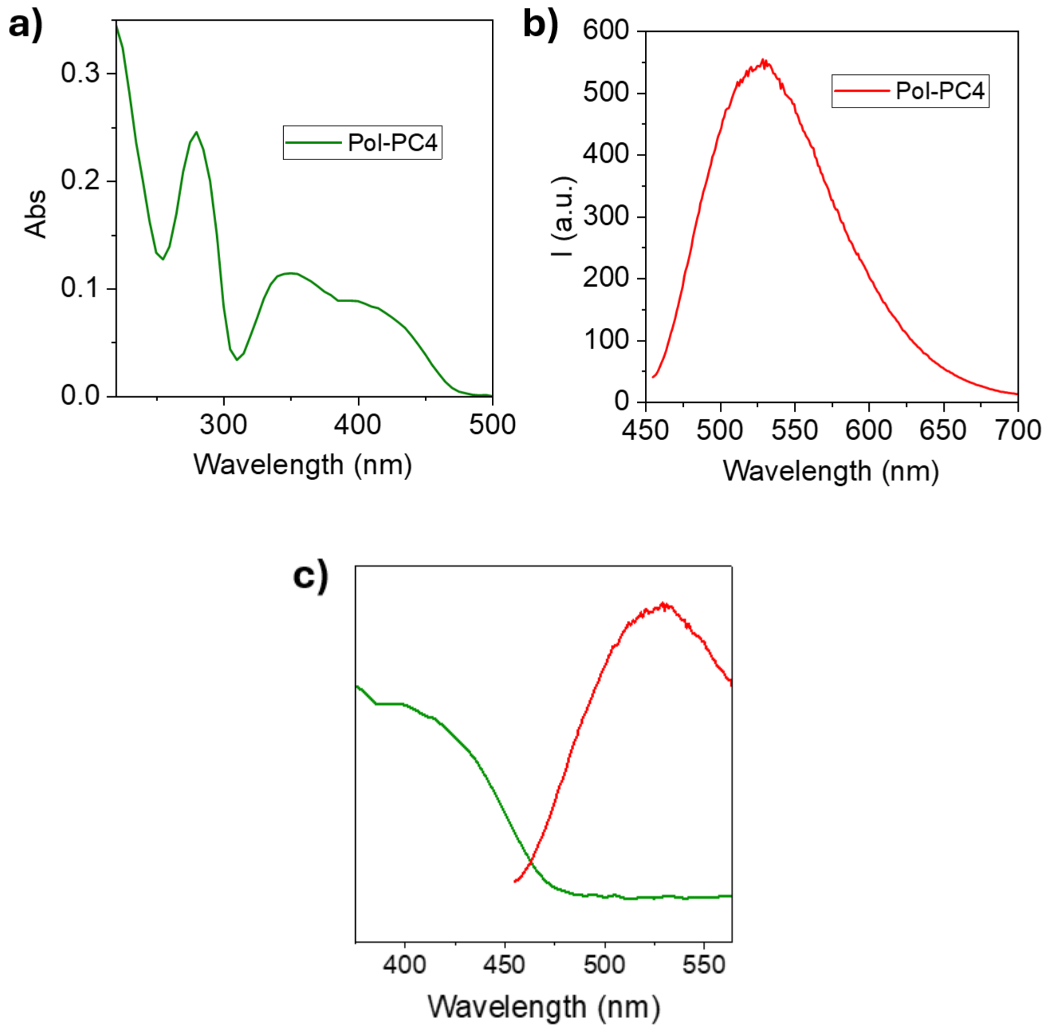

Figure S23. a) absorption and b) emission spectrum of P(PC4MA-*co*-OEGMA), c) intersection point of the spectra resulting in the crossing point at 460 nm. Converting results in $E_{0,0}$= 2.70 V.

P(PC6MA-*co*-OEGMA)

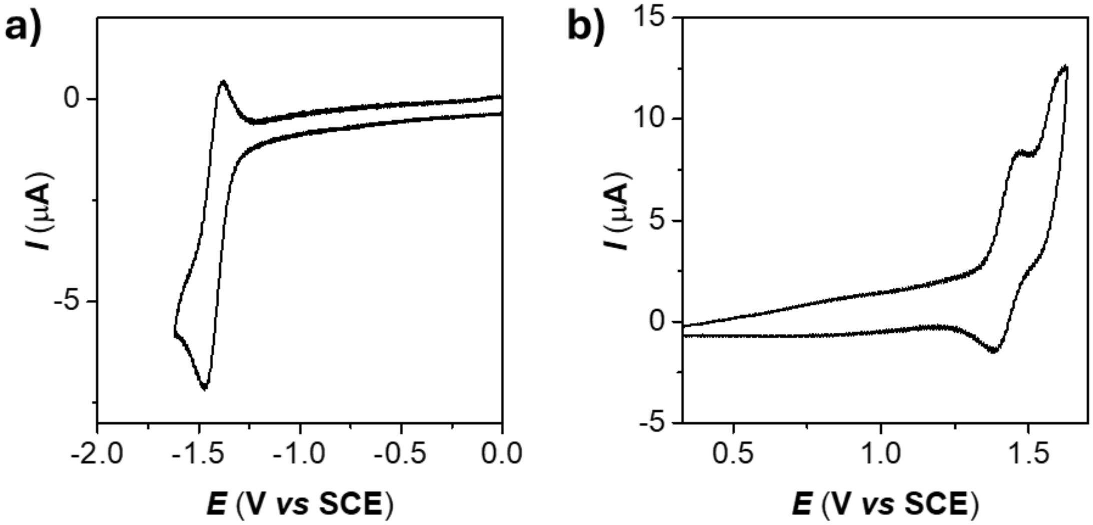

Figure S24. CV in the reduction a) and in the oxidation b) direction of a solution of 0.5 mM of P(PC6MA-*co*-OEGMA) in ACN using TEATFB 0.1 M as supporting electrolyte and a scan rate of 1 V/s. The concentration of the solution was calculated with respect to the estimated incorporation of photocatalyst.

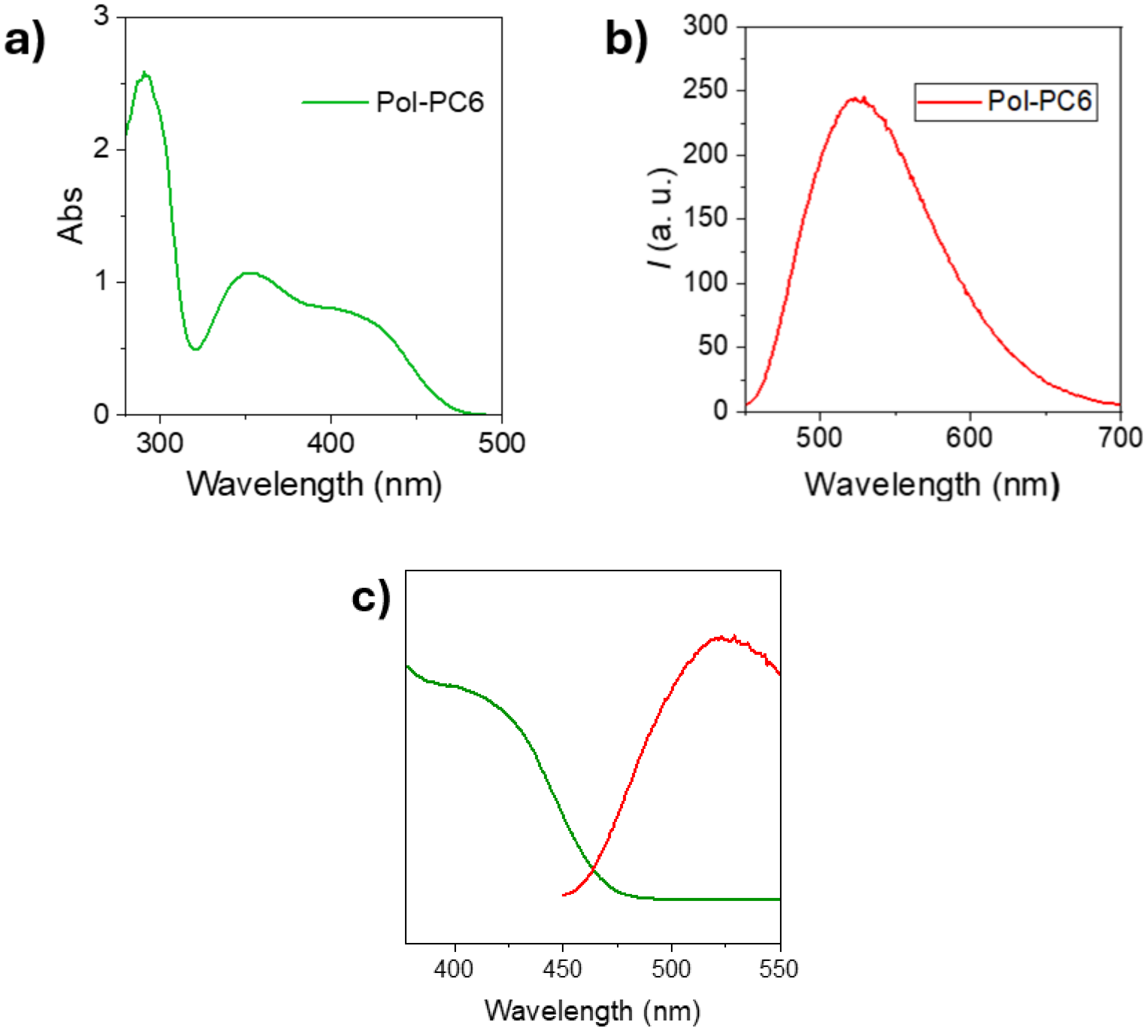

Figure S25. a) absorption and b) emission spectrum of P(PC6MA-*co*-OEGMA), c) intersection point of the spectra resulting in the crossing point at 464 nm. Converting results in $E_{0,0}$= 2.67 V.

Lifetimes measurements

Two different techniques were used for this purpose. Direct fluorescence (τ_p) was measured using time-correlated single-photon counting (TCSPC), a technique typically employed for short lifetimes (a few nanoseconds at most). In this method, a detector converts a single photon into an electronic pulse, and the TCSPC electronics accurately measures its arrival time relative to the excitation pulse. Thousands of arrival times are recorded showing their statistical intensity at a given time, from which the decay fitting provides the lifetime. [4]

For long delayed fluorescence (τ_d), a more suitable method is Multi-Channel Scaling (MCS), a photon counting technique generally used for measuring longer lifetimes. In MCS, multiple photons are collected in each sweep of

the time window, quickly accumulating decays across timescales ranging from several hundred nanoseconds to seconds.[5]

All fluorescence decays were analyzed with the Fluoracle Software using the IRF convolution fitting procedure.

Lifetimes were calculated for all PCs and PC-bearing copolymers and polymer brushes at room temperature using ACN as solvent (same solvent of the benchmark reaction). Moreover, for PCs lifetimes were calculated also at 77 K using MeTHF as solvent (suitable solvent for measurements at very low temperature).

Lifetimes summary at room temperature in ACN

Table S3. Summary of lifetime measurements at room temperature in acetonitrile.

PC	τ_p (ns)	τ_d (ns)
PC1	14.4	1465
PC2	3.2	nd
PC3	2.3	817
PC4	7.2	$86 \cdot 10^3$
PC5	1.5	nd
PC6	2.3	$66 \cdot 10^3$
P(PC1MA-*co*-OEGMA)	14.2	1465
P(PC4MA-*co*-OEGMA)	7.2	$84 \cdot 10^3$
P(PC6MA-*co*-OEGMA)	2.3	$70 \cdot 10^3$
SiO_x- P(PC1MA-*co*-OEGMA)	14.5	1490

PC1 lifetimes in ACN

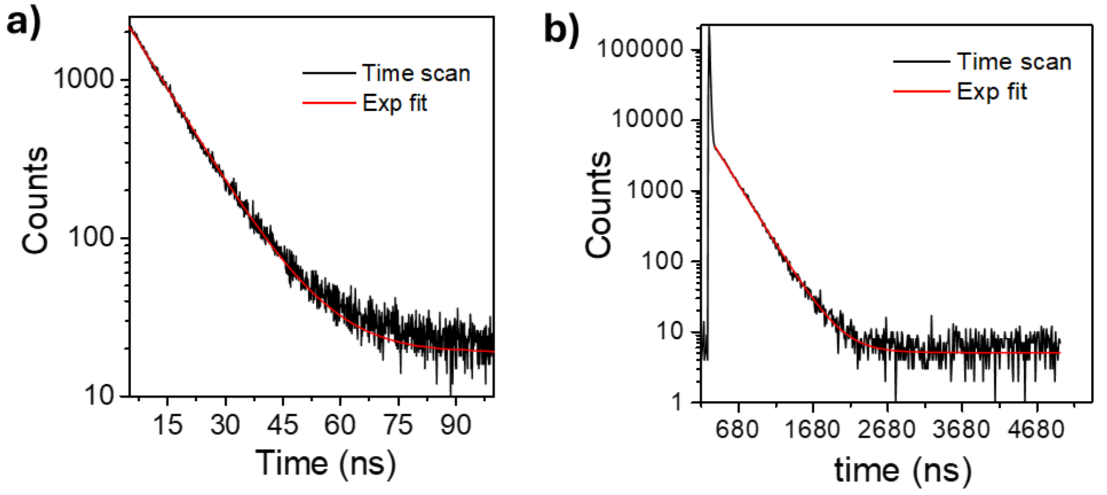

Figure S26. a) τ_p measurement of PC1 at room temperature in acetonitrile with TCSPC technique resulting in 14.4 ns. b) τ_d measurement of PC1 at room temperature in acetonitrile with MCS tenique resulting in 1465 ns.

PC2 lifetimes in ACN

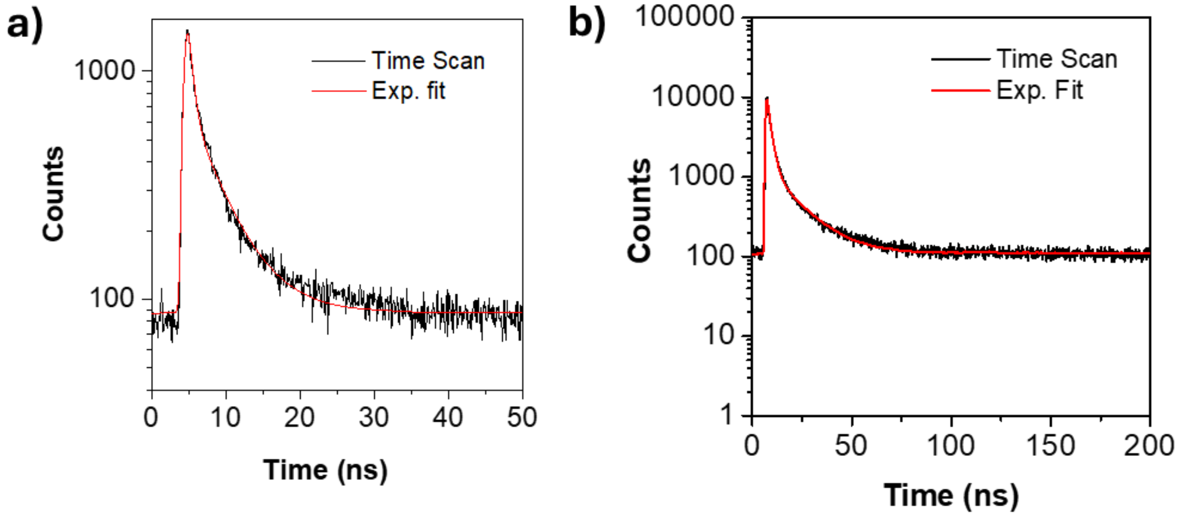

Figure S27. a) τ_p measurement of PC2 at room temperature in acetonitrile with TCSPC technique resulting in 3.2 ns. b) τ_d measurement of PC2 at room temperature in acetonitrile with MCS technique showed no presence of a second lifetime.

PC3 lifetimes in ACN

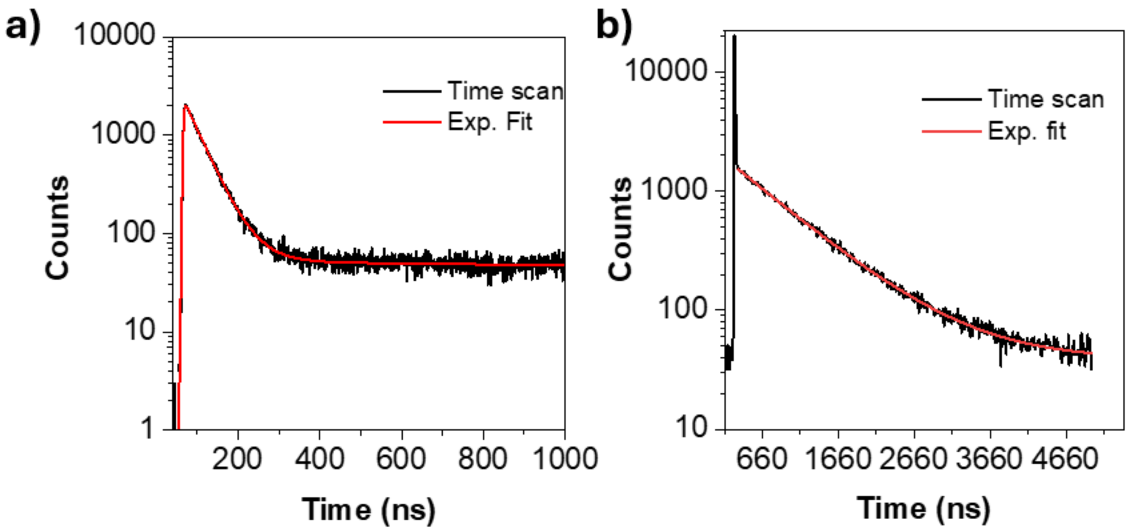

Figure S28. a) τ_p measurement of PC3 at room temperature in acetonitrile with TCSPC technique resulting in 2.3 ns. b) τ_d measurement of PC3 at room temperature in acetonitrile with MCS technique resulting in 817 ns.

PC4 lifetimes in ACN

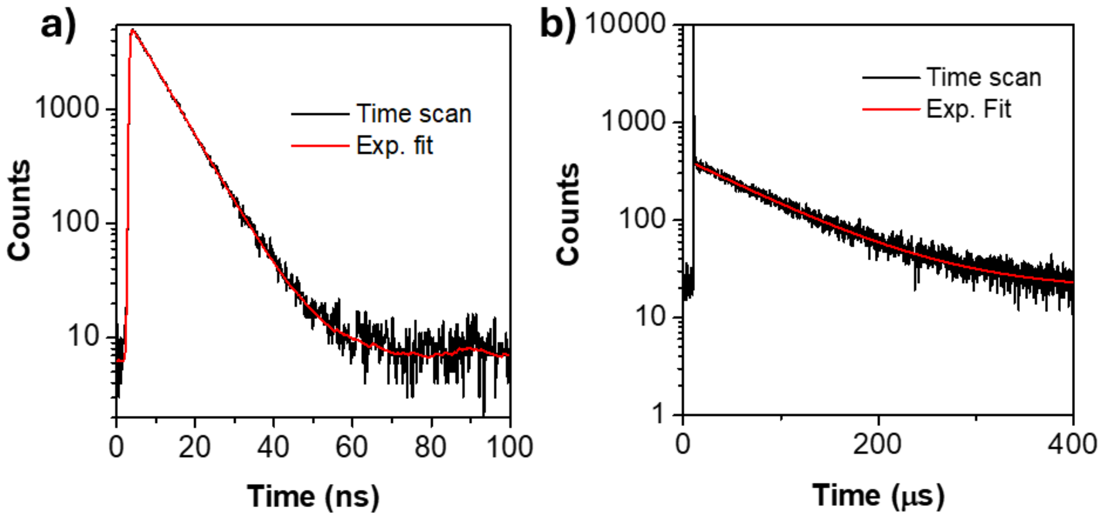

Figure S29. a) τ_p measurement of PC4 at room temperature in acetonitrile with TCSPC technique resulting in 7.2 ns. b) τ_d measurement of PC4 at room temperature in acetonitrile with MCS technique resulting in 86 μs.

PC5 lifetimes in ACN

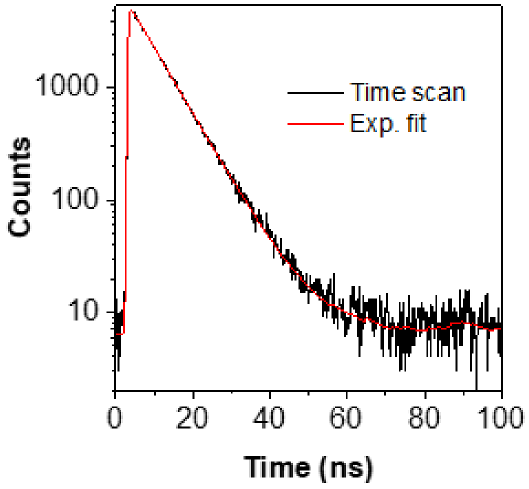

Figure S30. a) τ_p measurement of PC5 at room temperature in acetonitrile with TCSPC technique resulting in 1.5 ns. In this case τ_d lifetime was not detected.

PC6 lifetimes in ACN

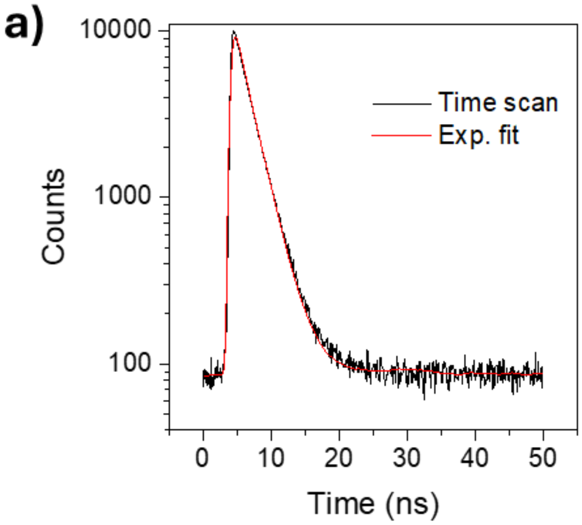

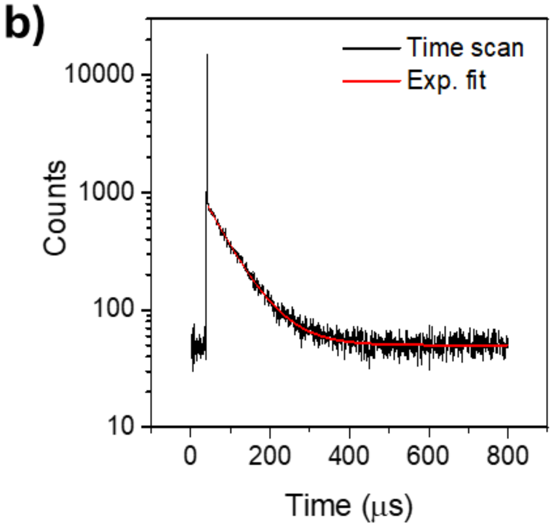

Figure S31. a) τ_p measurement of PC6 at room temperature in acetonitrile with TCSPC technique resulting in 2.3 ns. b) τ_d measurement of PC6 at room temperature in acetonitrile with MCS technique resulting in 66 μs.

P(PC1MA-*co*-OEGMA) lifetimes in ACN

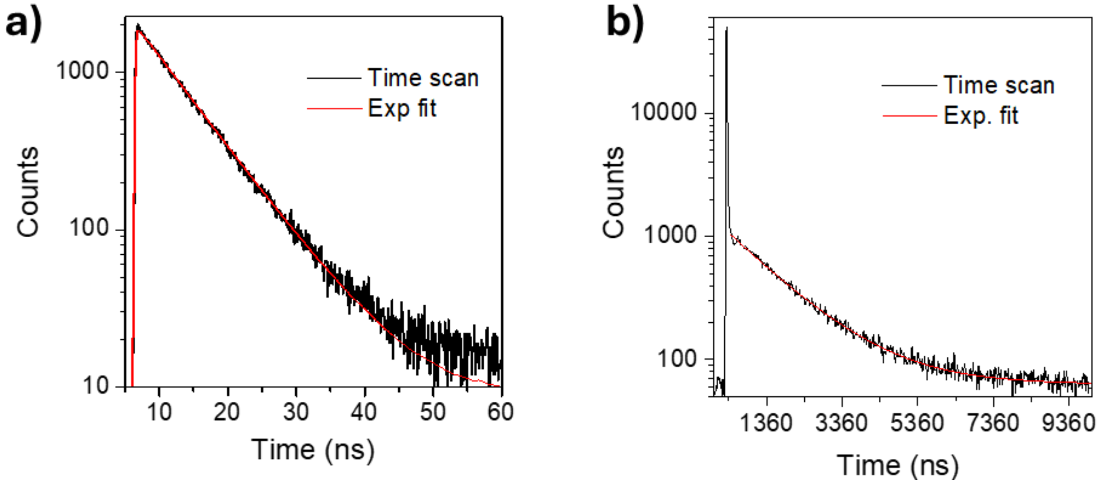

Figure S32. a) $\boldsymbol{\tau_p}$ measurement of P(PC1MA-*co*-OEGMA) at room temperature in acetonitrile with TCSPC technique resulting in 14.2 ns. b) $\boldsymbol{\tau_d}$ measurement of P(PC1MA-*co*-OEGMA) at room temperature in acetonitrile with MCS technique resulting in 1.45 µs.

P(PC4MA-*co*-OEGMA) lifetimes in ACN

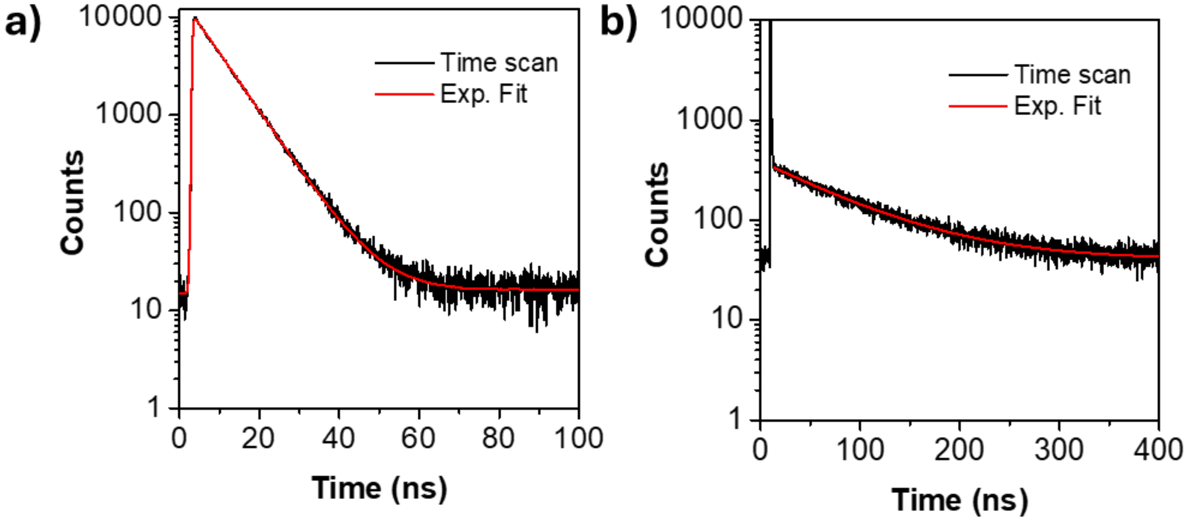

Figure S33. a) $\boldsymbol{\tau_p}$ measurement of P(PC4MA-*co*-OEGMA) at room temperature in acetonitrile with TCSPC technique resulting in 7.2 ns. b) $\boldsymbol{\tau_d}$ measurement of P(PC4MA-*co*-OEGMA) at room temperature in acetonitrile with MCS technique resulting in 84 µs.

P(PC6MA-*co*-OEGMA) lifetimes in ACN

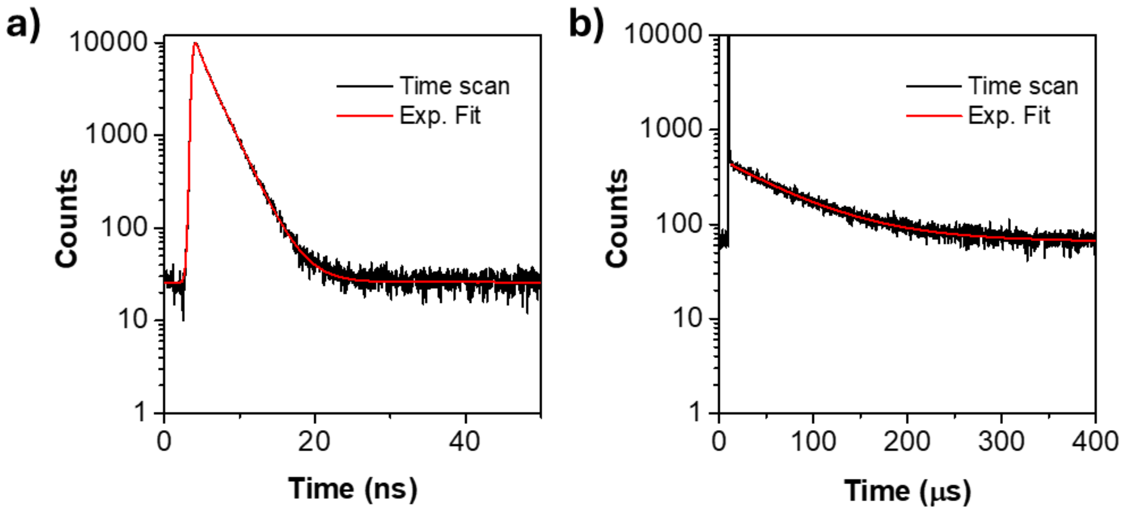

Figure S34. a) τ_p measurement of P(PC6MA-*co*-OEGMA) at room temperature in acetonitrile with TCSPC technique resulting in 7.2 ns. b) τ_d measurement of P(PC6MA-*co*-OEGMA) at room temperature in acetonitrile with MCS technique resulting in 84 μs.

SiO_x-P(PC1MA-*co*-OEGMA) lifetimes in ACN

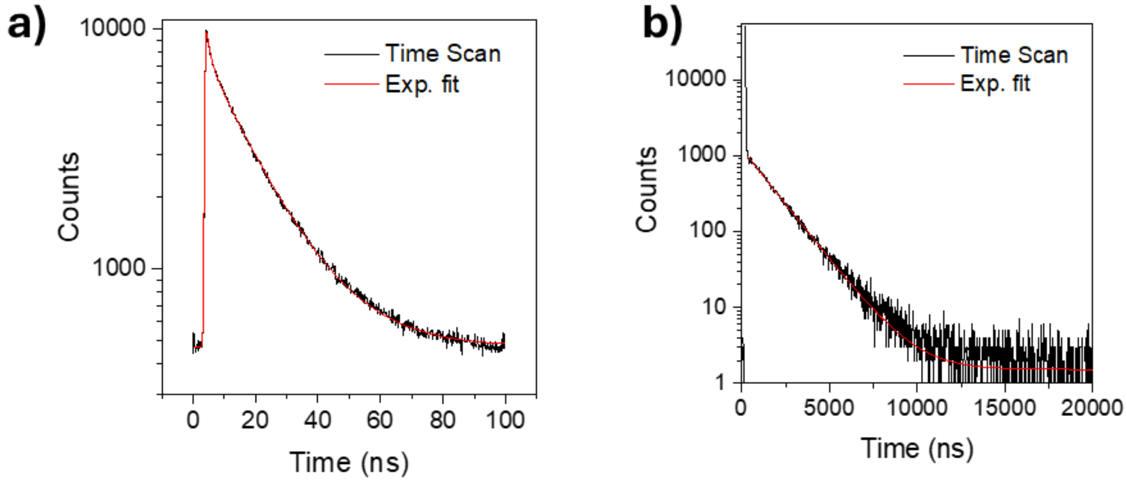

Figure S35. a) τ_p measurement of SiO_2-P(PC1MA-*co*-OEGMA) at room temperature in acetonitrile with TCSPC technique resulting in 14.5 ns. b) τ_d measurement of SiO_2-P(PC1MA-*co*-OEGMA) at room temperature in acetonitrile with MCS technique resulting in 1.5 μs.

Lifetimes summary in MeTHF

Table S4. Prompt and delayed fluorescence lifetime in MeTHF at room temperature and at 77K in liquid nitrogen.

PC	τ_p at room T (ns)	τ_d at room T (ns)	τ_p at 77K (ns)
PC1	21.8	6990	10.2
PC2	4.7	nd	-[a]
PC3	4.0	4046	1.7
PC4	3.9	92000	2.1
PC5	4.8	nd	-[a]
PC6	2.0	58000	0.6

[a] Measurement not performed since delayed fluorescence was not detected.

PC1 lifetimes in MeTHF

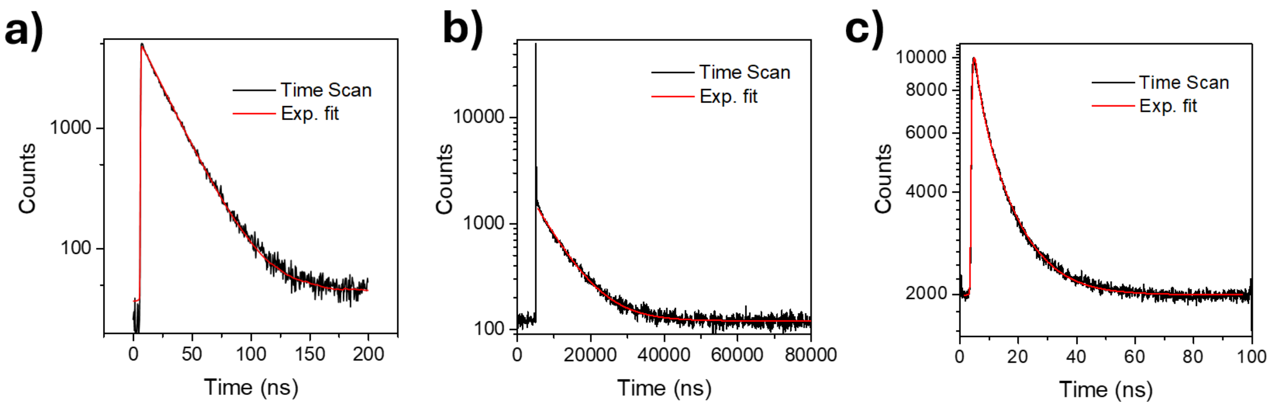

Figure S36. a) τ_p measurement of PC1 at room temperature in MeTHF with TCSPC technique resulting in 21.8 ns; b) τ_d measurement of PC1 at room temperature in MeTHF with MCS technique resulting in 6.99 μs; c) τ_p measurement of PC1 in MeTHF at 77 K yielded a lifetime of 10.2 ns, with no indication of a secondary decay component, supporting its assignment as delayed fluorescence rather than phosphorescence.

PC2 lifetimes in MeTHF

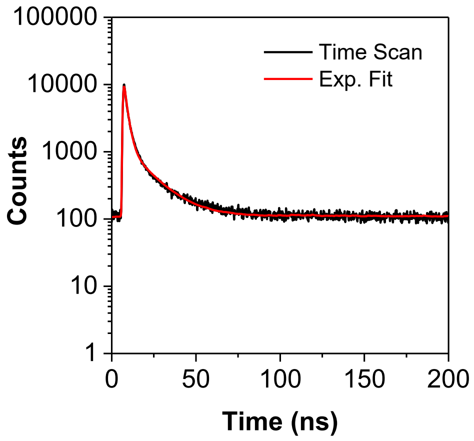

Figure S37. τ_p measurement of PC2 at room temperature in MeTHF with TCSPC technique resulting in 4.7 ns. The clear absence of delayed emission at room temperature made lifetime measurements at 77 K unnecessary

PC3 lifetimes in MeTHF

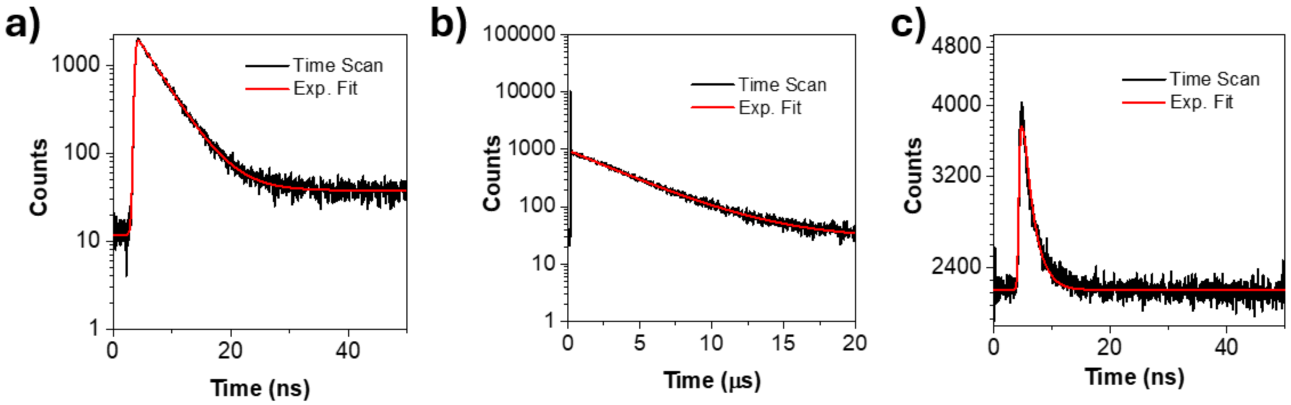

Figure S38. a) τ_p measurement of PC3 at room temperature in MeTHF with TCSPC techni resulting in 4.0 ns. b) τ_d measurement of PC3 at room temperature in MeTHF with MCS technique resulting in 4.05 µs. c) τ_p measurement of PC3 in MeTHF at 77 K yielded a lifetime of 1.7 ns, with no indication of a secondary decay component, supporting its assignment as delayed fluorescence rather than phosphorescence.

PC4 lifetimes in MeTHF

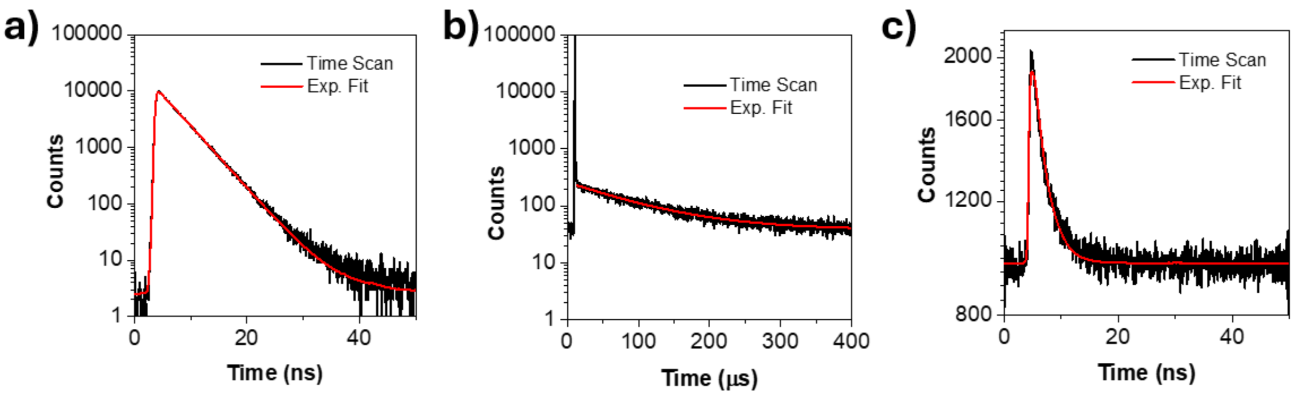

Figure S39. a) τ_p measurement of PC3 at room temperature in MeTHF with TCSPC technique resulting in 3.9 ns. b) τ_d measurement of PC3 at room temperature in MeTHF with MCS technique resulting in 92 µs. c) τ_p measurement of PC3 in MeTHF at 77 K yielded a lifetime of 2.1 ns, with no indication of a secondary decay component, supporting its assignment as delayed fluorescence rather than phosphorescence.

PC5 lifetimes in MeTHF

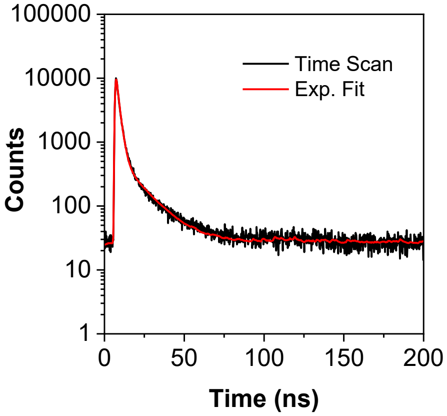

Figure S40. τ_p measurement of PC5 at room temperature in MeTHF with TCSPC technique resulting in 4.8 ns. The clear absence of delayed emission at room temperature made lifetime measurements at 77 K unnecessary.

PC6 lifetimes in MeTHF

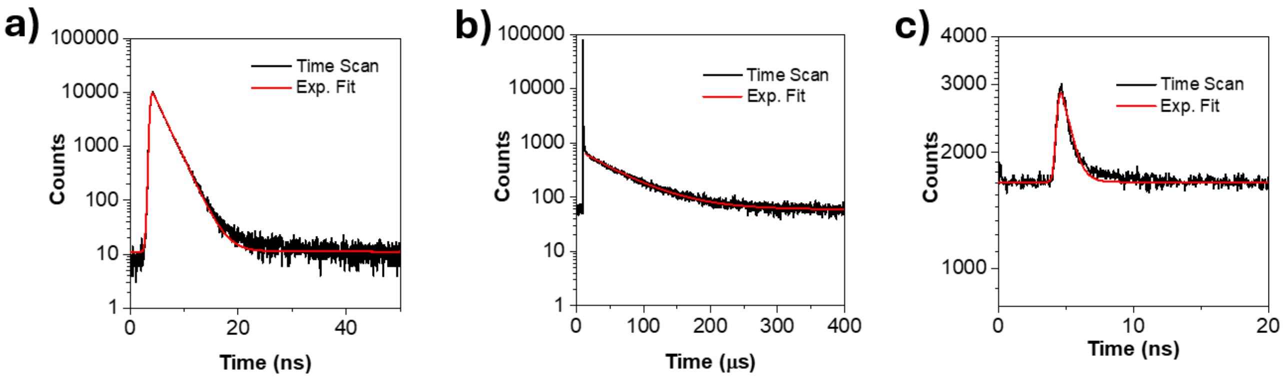

Figure S41. a) τ_p measurement of PC6 at room temperature in MeTHF with TCSPC technique resulting in 2 ns. b)) τ_d measurement of PC3 at room temperature in MeTHF with MCS technique resulting in 58 μs. c) τ_p measurement of PC3 in MeTHF at 77 K yielded a lifetime of 0.6 ns, with no indication of a secondary decay component, supporting its assignment as delayed fluorescence rather than phosphorescence.

Absorption and emission calibrations

PC1 absorption calibration

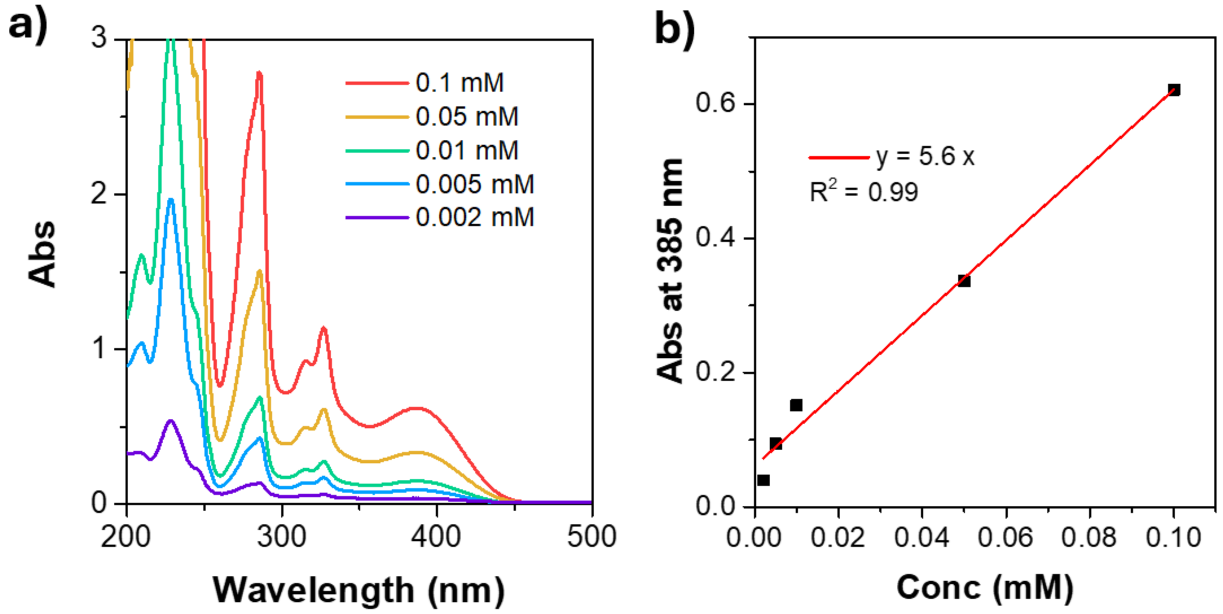

Figure S42. a) Absorption spectra of PC1 at different concentrations; b) Calibration line built using the absorption values at 385 nm.

P(PC1MA-*co*-OEGMA) emission calibration

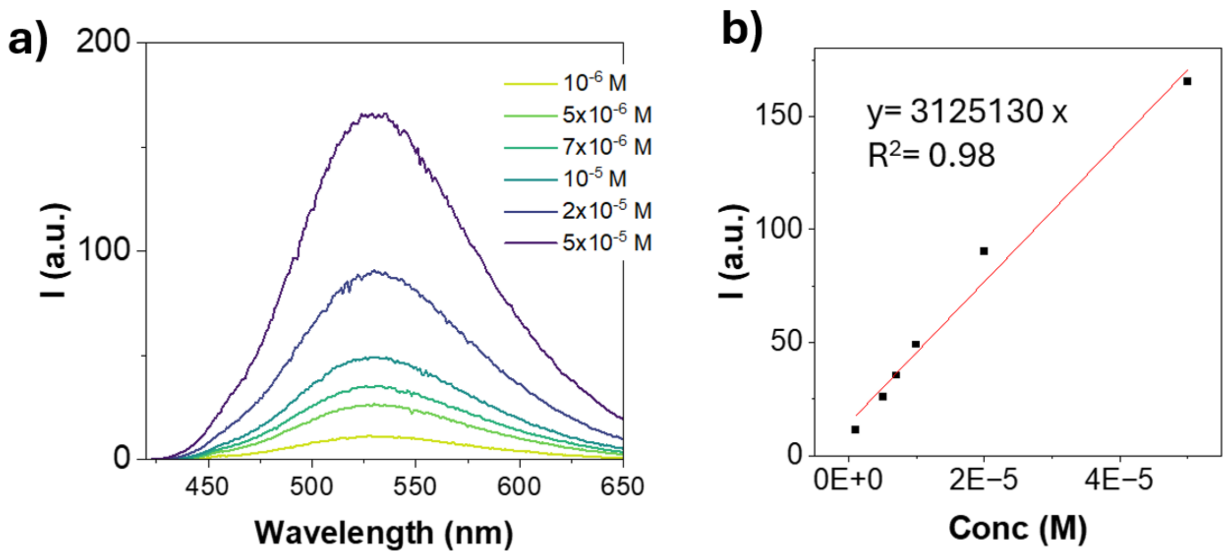

Figure S43. a) Emission spectra of P(PC1MA-*co*-OEGMA) at different concentrations. b) Calibration line built using the emission values at 525 nm.

PC4 absorption calibration

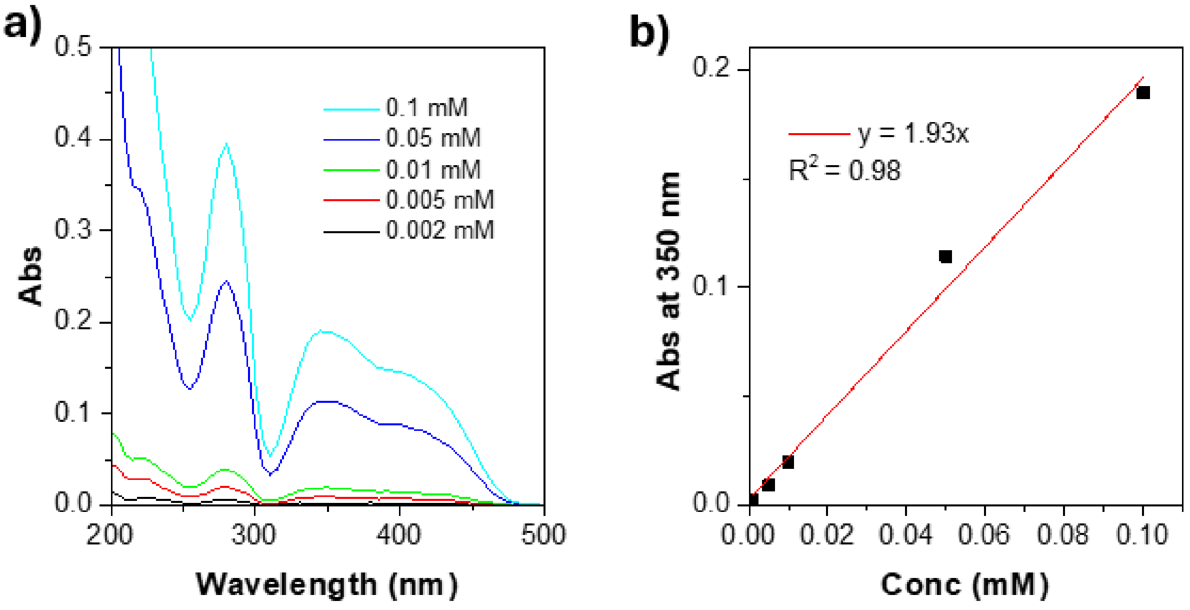

Figure S44. a) Absorption spectra of PC4 at different concentrations. b) Calibration line built using the absorption values at 350 nm.

PC6 absorption calibration

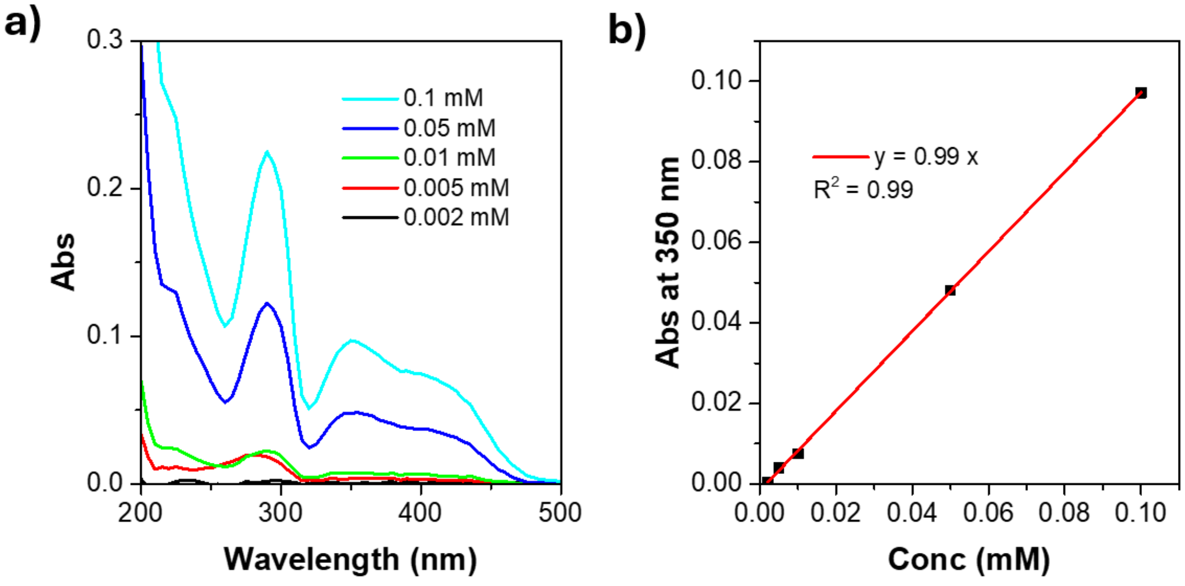

Figure S45. a) Absorption spectra of PC6 at different concentrations; b) calibration line built using the absorption values at 350 nm.

Transmission electron microscopy (TEM) pictures

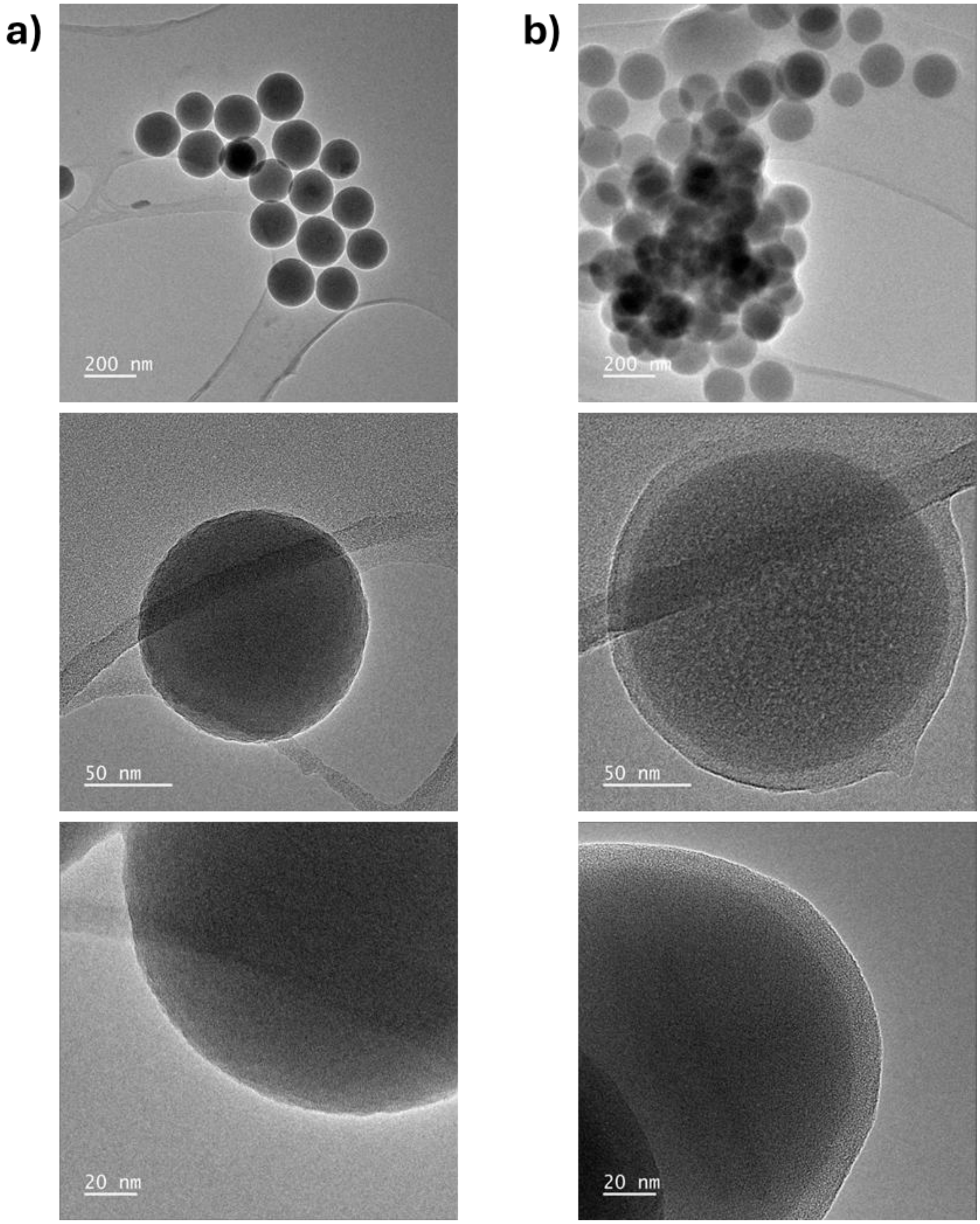

Figure S46. a) TEM pictures of pristine silica nanoparticles before surface functionalization; b) TEM images of the nanoparticles after functionalization via SI-ARGET ATRP clearly show a core–shell hybrid system with polymer brushes.

NMR of photoreaction’s crudes and purified products

Povarov-type photoreaction

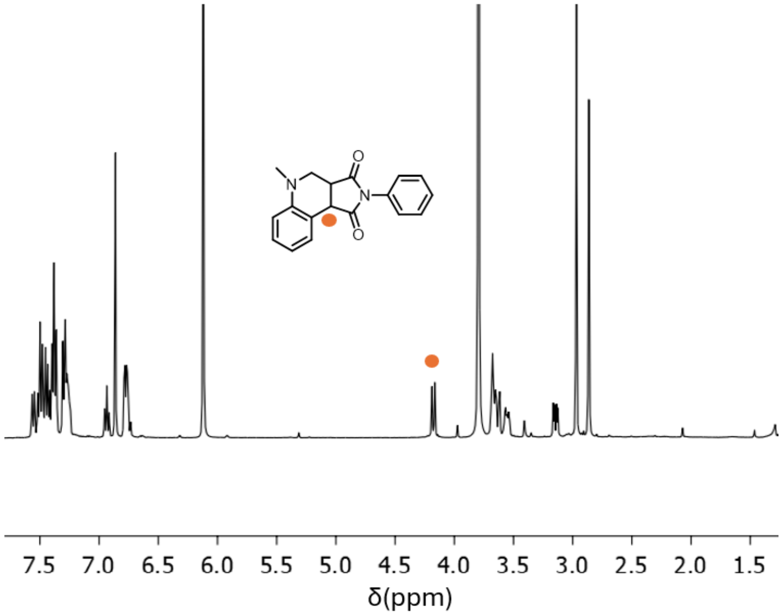

Figure S47. ^{1}H-NMR spectrum of the crude of Povarov photoreaction between N,N-dimethylaniline and N-phenyl maleimide added with 1,3,5-Trimethoxy benzene as internal standard (3.7 and 6.1 ppm). In orange is indicated the diagnostic peak used for the quantitative calculation of the reaction yield.

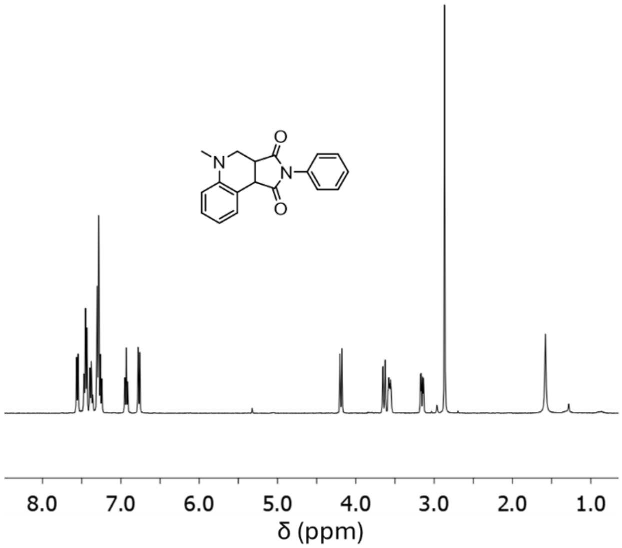

Figure S48. ^{1}H-NMR spectrum of the purified product. ^{1}H NMR (400 MHz, $CDCl_3$): δ (ppm) 7.56 (d, J = 7.6 Hz, 1H), 7.45 (t, J = 7.6 Hz, 2H), 7.38 (t, J = 7.3 Hz, 1H), 7.32 – 7.21 (m, 3H), 6.93 (t, J = 7.5 Hz, 1H), 6.77 (d, J = 8.2 Hz, 1H), 4.19 (d, J = 9.6 Hz, 1H), 3.64 (d, J = 11.5 Hz, 1H), 3.57 (m,1H), 3.15 (m,1H), 1.58 (s, 3H).

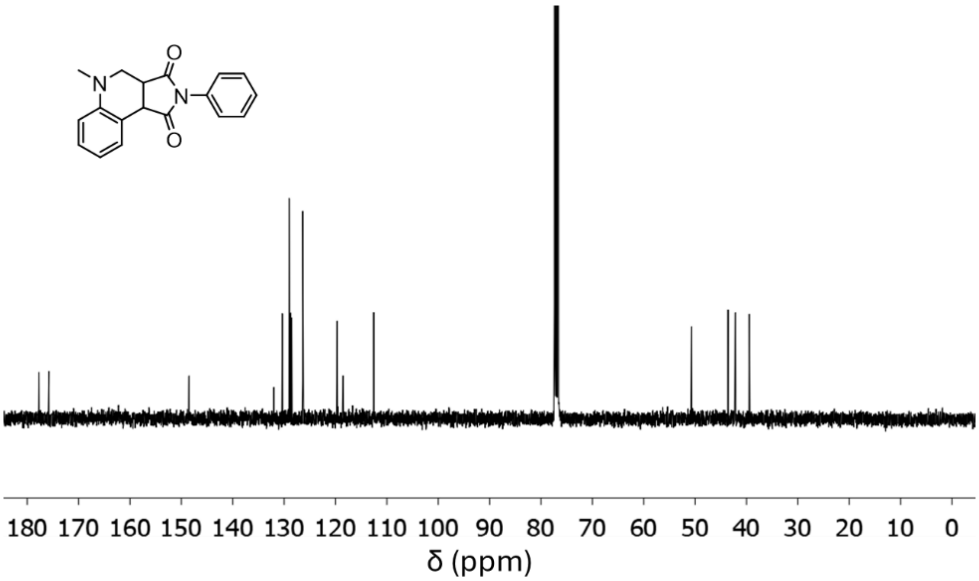

Figure S49. ^{13}C NMR of the purified product ^{13}C NMR (100 MHz, $CDCl_3$): δ (ppm) 177.7, 175.8, 148.5, 132.0, 130.3, 129.0, 128.7, 128.5, 126.4, 119.7, 118.5, 112.5, 50.7, 43.6, 42.2, 39.4.

Photo hydroxylation in water of phenyl boronic acid pinacol ester

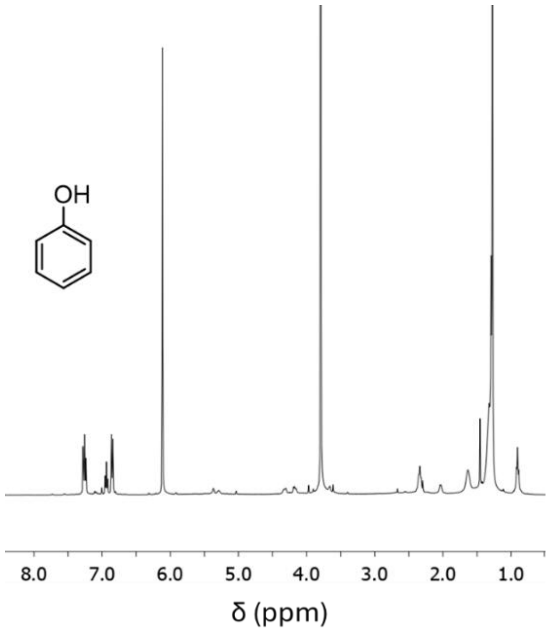

Figure S50. ^{1}H-NMR spectrum of the extracted crude of photohydroxylation reaction in water added with 1,3,5-Trimethoxy benzene as internal standard (3.7 and 6.1 ppm). The aromatic peaks were used for the quantitative calculation the yield.

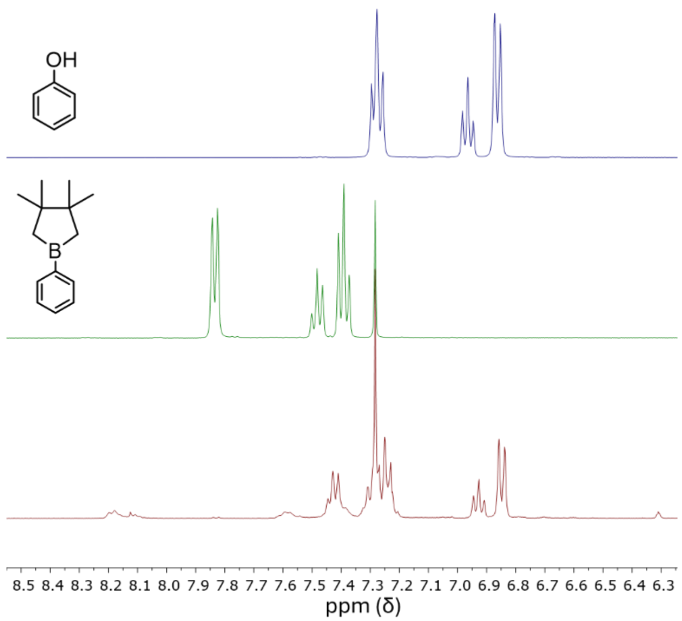

Figure S51. ^{1}H-NMR spectra of phenol (in blue) that is the product of the photohydroxylation reaction in water. Phenylboronic acid pinacol ester (in green) corresponding to the starting material of the reaction and finally, in red, a representative crude of the reaction. It is possible to observe full conversion of starting material.

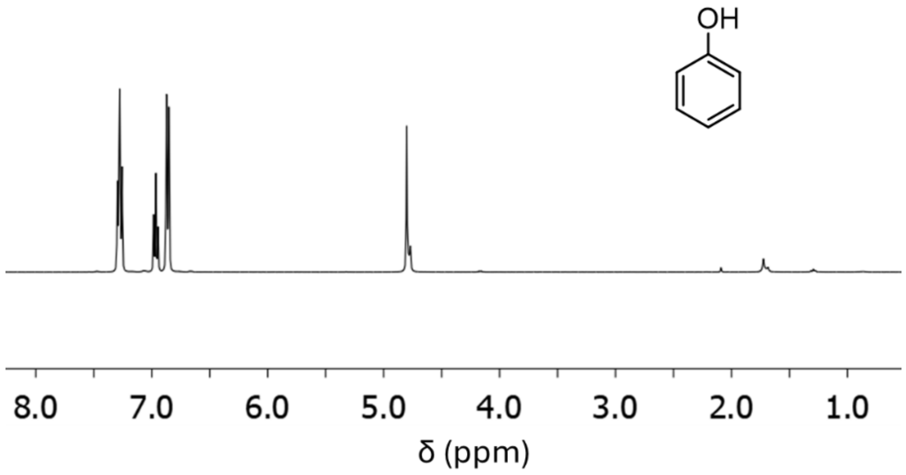

Figure S52. ^{1}H-NMR spectrum of the purified product (phenol). ^{1}H NMR (400 MHz, $CDCl_3$): δ (ppm) 7.27 (t, J = 7.4 Hz, 2H), 6.94 (t, J = 7.4 Hz, 1H), 6.85 (d, J = 8.0 Hz, 2H), 4.80 (s, 1H).

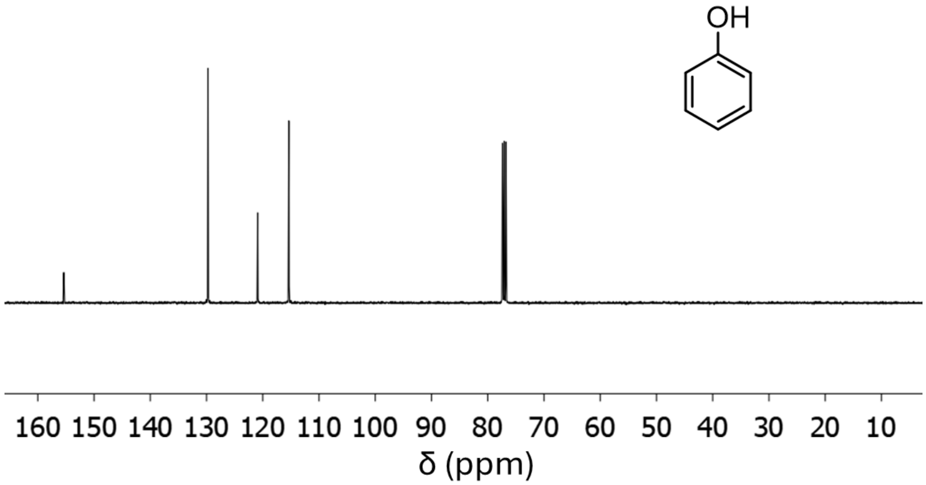

Figure S53. ^{13}C NMR of the purified product ^{13}C NMR (100 MHz, $CDCl_3$): δ (ppm) 115.3, 121.0, 129.8, 155.5.

[2+2] cycloaddition of 4-acetoxystyrene

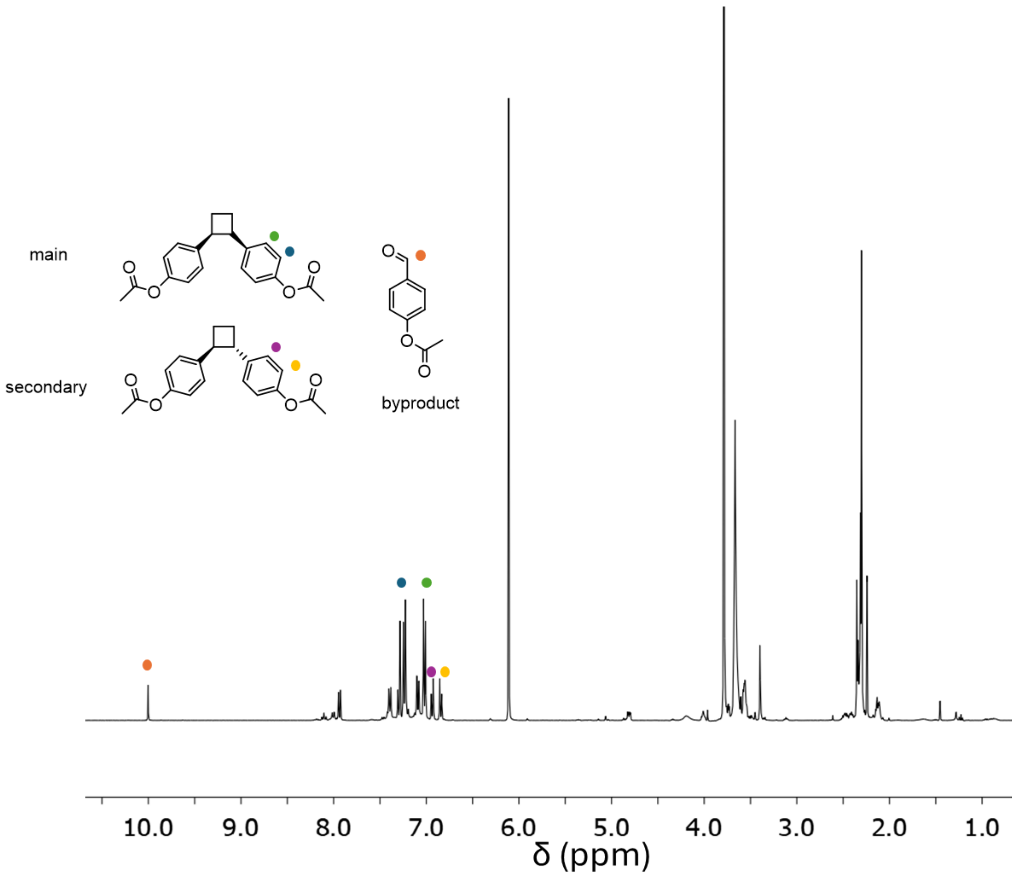

Figure S54. ^{1}H-NMR spectrum of the crude of [2+2] cycloaddition of 4-acetoxystyrene. in water added with 1,3,5-Trimethoxy benzene as internal standard (3.7 and 6.1 ppm). The crude shows the presence of trans and cis isomers (trans as primary product) and presence of 4-acetoxybenzalaldehide as byproduct.[6] The aromatic peaks were used for the quantitative calculation the yield.

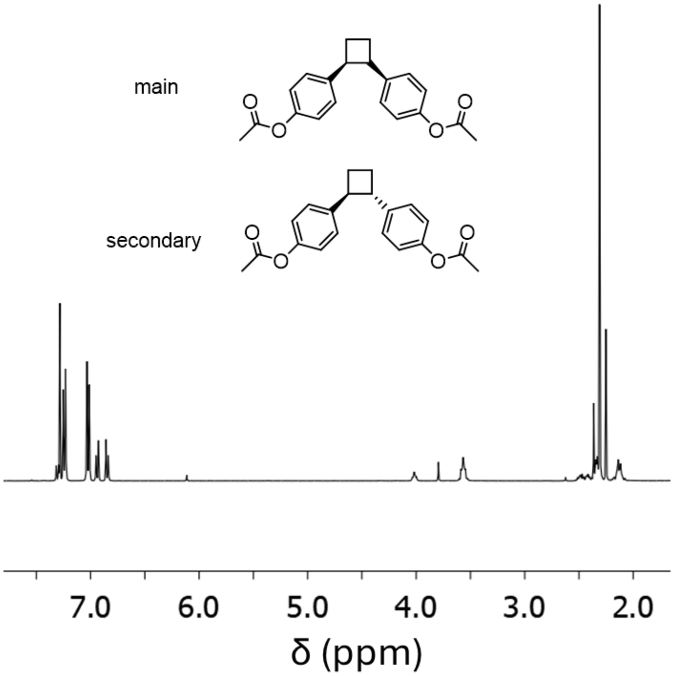

Figure S55. Trans/cis ratio = 1:0.3. ^{1}H NMR (400 MHz, $CDCl_3$): δ (ppm) 7.23-7.21 (d, 4H, CH2CHCCH Ar), 7.01-6.99 (d, 4H, OCCH Ar), 6.93-6.90 (d, 0.66H, CH2CHCCH Ar), 6.83-6.81 (d, 0.66H, OCCH Ar), 4.01-3.97 (m, 0.4H, CH2CHC), 3.56-3.52 (m, 2H, CH2CHC), 2.46-2.31 (m, 2.5H, CH2CH2), 2.29 (s, 6H, CH3), 2.23 (s, 1H, CH3), 2.11 (m, 2.2H, CH2CH2).

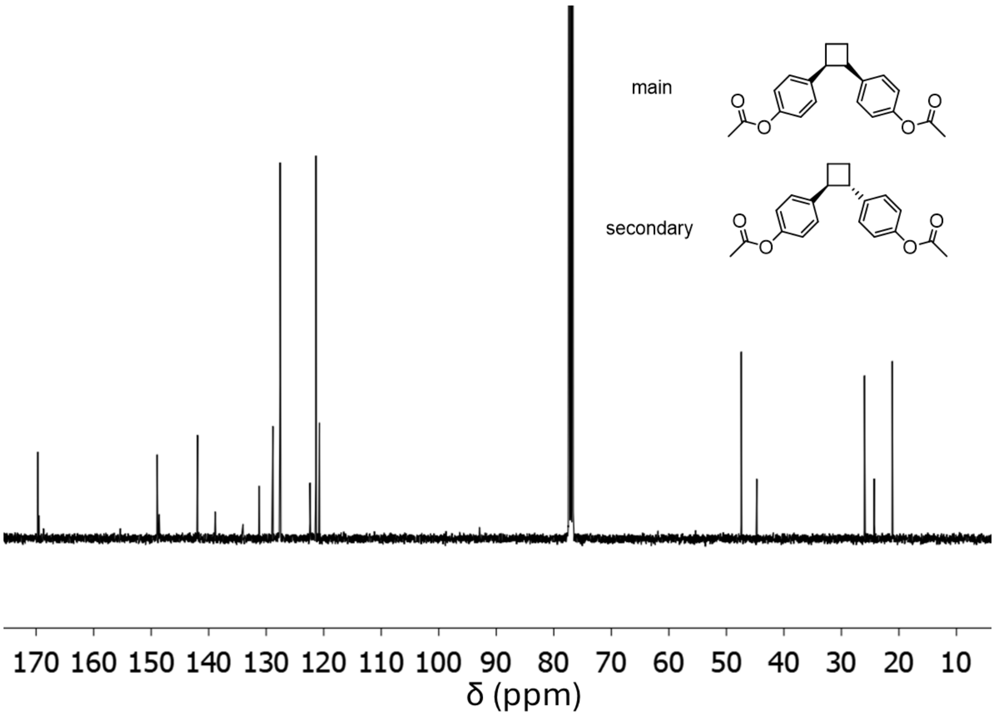

Figure S56. ^{13}C NMR (100 MHz, $CDCl_3$): δ (ppm) 21.3, 24.4, 26.1, 29.8, 44.8, 47.5, 120.9, 121.5, 129.0, 142.0, 149.1, 169.8.

Kinetic incorporation of PCMA

Statistical incorporation of PCs within copolymers was confirmed by following the copolymerization of 5 mol% of PC6MA with OEGMA through ^{1}H-NMR. Analysis of samples withdrawn from the crude polymerization mixture did not enable a clear evaluation of PC6MA incorporation because of the very small integral and small shift of the signals correlated to PC6MA vinyl protons (**Figure S57**). Hence, 1 mL-aliquots of reaction mixtures were removed from the reaction after 1, 2, 4 and 6 hours, purified by dialysis and later on analyzed by ^{1}H-NMR. For each purified aliquot the incorporation percentage was estimated considering the ratio between the integral of the aromatic peaks (correlated to the PC) and the polymer peak at 4.1 ppm diagnostic of the methylene of the PEG side chains in alpha position (circled in red in **Figure S54**). It was found that the incorporation was included between 4.2 and 4.7 mol% suggesting a statistical incorporation of PC6MA within the copolymers.

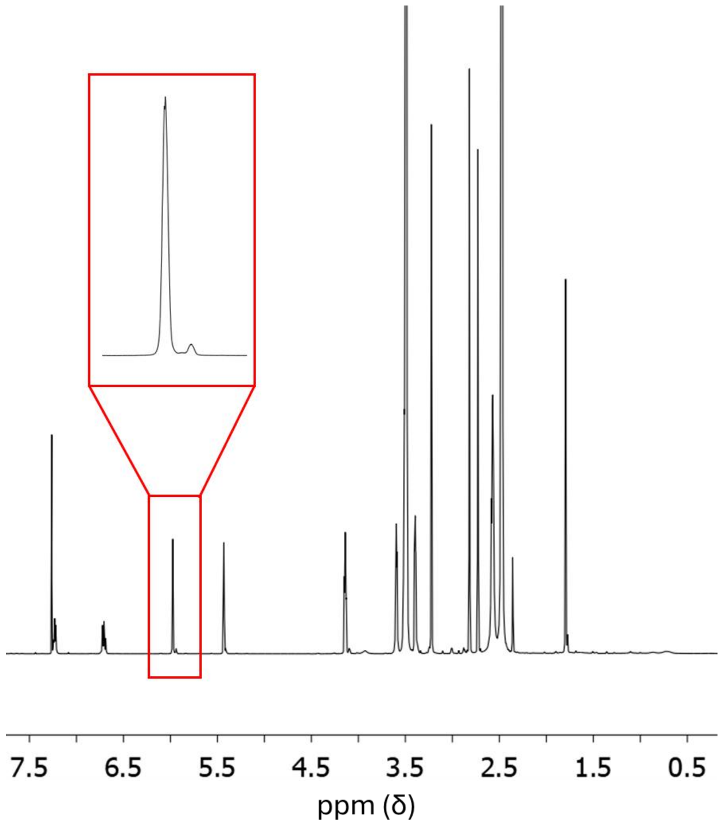

Figure S57. Crude of t= 0 of the ARGET ATRP copolymerization of OEGMA (95%) and PC6MA (5%).

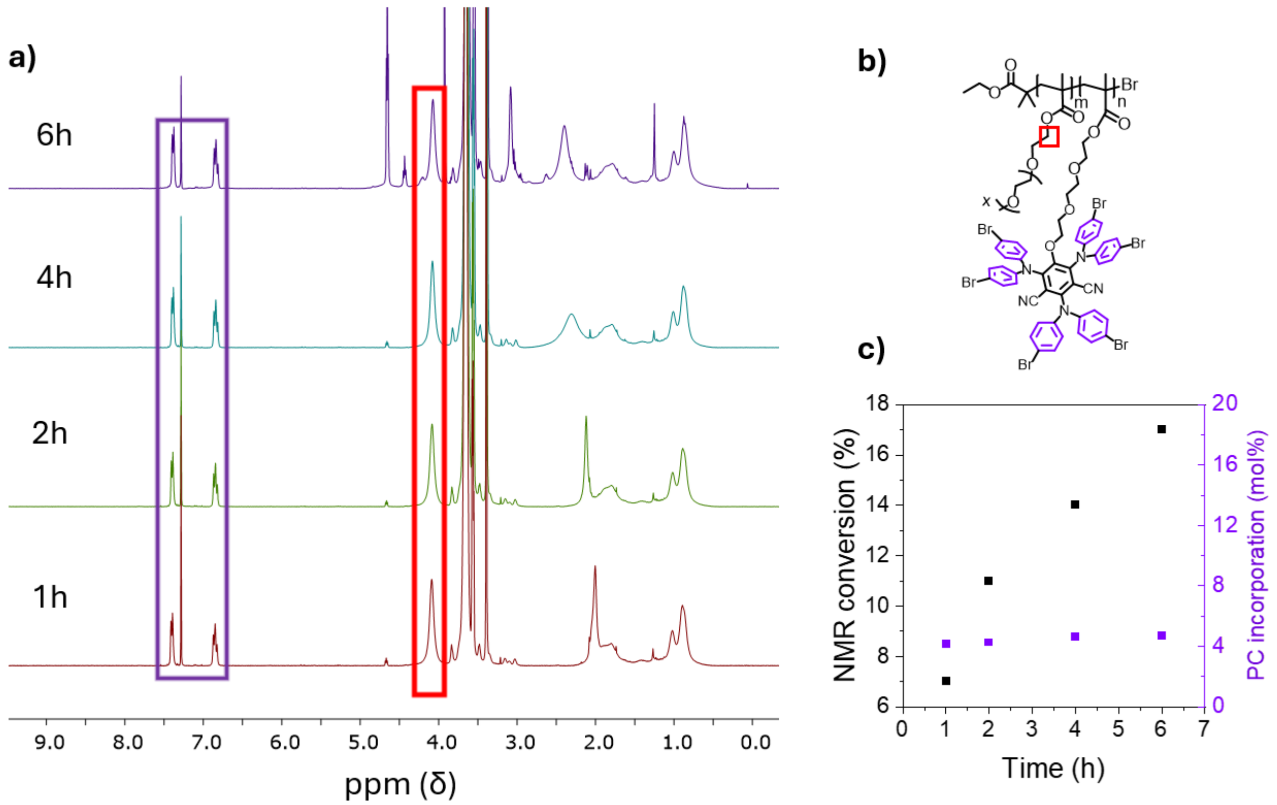

Figure S58. (a) ^{1}H-NMR spectra (400 MHz in $CDCl_3$) of the purified aliquots of P(PC6MA-*co*-OEGMA) after 1, 2, 4 and 6 hours of reaction. In purple the aromatic peaks corresponding to the catalytic moiety are highlighted, in red the diagnostic peak by the polymer. (b) Chemical structure of P(PC6MA-*co*-OEGMA). In purple the signals corresponding to the aromatic groups are highlighted, while in red the methylene groups responsible for the polymer diagnostic peak at 4.1 ppm are emphasized. (c) Total conversion of the monomer during copolymerization of PC6MA and OEGMA, in purple the percentage of photocatalyst for each kinetic point estimated by ^{1}H-NMR.

REFERENCES

(1) Bortolato, T.; Cuadros, S.; Simionato, G.; Dell'Amico, L. The advent and development of organophotoredox catalysis. *Chemical Communications* **2022**, *58* (9), 1263-1283, 10.1039/D1CC05850A. DOI: 10.1039/D1CC05850A.
(2) Liang, Z.; Xu, S.; Tian, W.; Zhang, R. Eosin Y-catalyzed visible-light-mediated aerobic oxidative cyclization of N,N-dimethylanilines with maleimides. *Beilstein Journal of Organic Chemistry* **2015**, *11*, 425-430. DOI: 10.3762/bjoc.11.48.
(3) Buzzetti, L.; Crisenza, G. E. M.; Melchiorre, P. Mechanistic Studies in Photocatalysis. *Angewandte Chemie International Edition* **2019**, *58* (12), 3730-3747. DOI: https://doi.org/10.1002/anie.201809984.
(4) Phillips, D.; Drake, R. C.; O'Connor, D. V.; Christensen, R. L. Time Correlated Single-Photon Counting (Tcspc) Using Laser Excitation. *Instrumentation Science & Technology* **1985**, *14* (3-4), 267-292. DOI: 10.1080/10739148508543581.
(5) Wang, X.; Tsuji, T.; Uchida, T.; Minami, S. *Multichannel photon-counting fluorimetric systems using optical fiber dynamic memory*; SPIE, 1992.

(6) Arena, D.; Verde-Sesto, E.; Rivilla, I.; Pomposo, J. A. Artificial Photosynthases: Single-Chain Nanoparticles with Manifold Visible-Light Photocatalytic Activity for Challenging “in Water” Organic Reactions. *Journal of the American Chemical Society* **2024**, *146* (21), 14397-14403. DOI: 10.1021/jacs.4c02718.
